# Identification of gamma-hydroxybutyrate (GHB)–β-ribose as a promising biomarker enabling prolonged detection of GHB intake

**DOI:** 10.1126/sciadv.aeh1261

**Published:** 2026-11-13

**Authors:** Kathrine Bohn Faldborg, Jørgen Bo Hasselstrøm, Lambert Kristiansen Sørensen, Camilla Bak Nielsen, Søren Lau Borcher Møller, Thomas B. Poulsen, Mogens Johannsen, Charlotte Uggerhøj Andersen, Kirstine Lykke Nielsen

**Affiliations:** ^1^ Department of Forensic Medicine, Aarhus University, Aarhus, Denmark.; ^2^ Department of Chemistry, Aarhus University, Aarhus, Denmark.; ^3^ Department of Clinical Pharmacology, Aarhus University Hospital, Aarhus, Denmark.

## Abstract

For several years, forensic toxicologists have sought biomarkers to extend the detection window of gamma-hydroxybutyric acid (GHB), a drug suspected to be involved in drug-facilitated sexual assaults yet difficult to detect due to its rapid elimination, endogenous presence, and postsampling formation. In this study, we used samples from 30 participants in a double-blind, controlled trial, randomized to placebo or a single oral dose (50 milligrams per kilogram) of sodium oxybate, a GHB salt. We collected whole blood and urine samples over 5 days and used untargeted metabolomics to search for biomarkers. The most promising feature, previously suggested as a GHB-pentose, was identified as GHB-β-ribose by chemical synthesis and liquid chromatography–tandem mass spectrometry. Quantification showed elevated levels of GHB-β-ribose for up to 48 in blood and 24 hours in urine after GHB intake compared with endogenous levels in the placebo group and authentic forensic cases. Thus, we present GHB-β-ribose as a very promising biomarker for GHB intake, with the potential to extend the detection window for GHB.

## INTRODUCTION

For decades, the detection of gamma-hydroxybutyric acid (GHB) in victims of drug-facilitated sexual assaults (DFSAs) has been a challenge in forensic toxicology (*1*, *2*). A DFSA involves drugs that incapacitate the victim, either taken voluntarily or administered covertly in proactive DFSAs. Although previous studies have suggested that the proactive DFSA occurs in 2 to 20% of sexual assaults, the prevalence remains largely unknown because victims are often examined after the involved drugs may have been eliminated (*3*). Sexual violence against women, both from nonintimate perpetrators (*4*) and intimate partners (*5*), is a global phenomenon (*4*, *5*). Thus, the DFSA likely affects thousands of people worldwide.

GHB, a former anesthetic, which emerged as a recreational drug in the 1980s (*1*), constitutes a particular challenge in the investigation of DFSAs. At lower doses, it induces euphoria and disinhibition, whereas increasing doses may result in sedation, memory loss, unconsciousness, and, ultimately, death (*1*). Furthermore, it comes as a colorless and odorless liquid easily mixed into drinks. These properties make it a critical substance to detect in DFSA victims. In addition, GHB is rapidly eliminated and endogenously present in low concentrations (*1*). Furthermore, it may be formed in the tube after blood or urine sampling, depending on the sampling and storage conditions (*6*). Thus, interpretation of GHB concentrations in forensic casework is currently based on practical cutoff values to prevent false positives, and the detection window of GHB ingestion is very short, ∼6 hours in blood and 10 to 12 hours in urine (*1*). For comparison, a previous study found that ∼75% of suspected DFSA victims had samples collected more than 12 hours after the assault (*7*), leaving a high risk of missing covert exposure to GHB in the victim and underestimating the prevalence of GHB intoxication in DFSA cases when relying solely on measured GHB concentrations.

Because of the challenges with detection of GHB, several studies have explored alternative biomarkers for GHB intake that persist longer in blood and urine than GHB itself. In this context, GHB-derived metabolites including succinic acid (*8*), glycolic acid, 2,4-dihydroxybutyric acid (DHB) and 3,4-DHB were proposed (*9*–*11*). Conjugated metabolites such as GHB-glucuronide and GHB-sulfate are particularly appealing as they are uniquely derived from GHB, and because they retain GHB in their chemical structure, they can be used as strong evidence of GHB intake (*12*, *13*). However, interindividual variability in endogenous levels and uncertain pharmacokinetics have limited the use of these initial conjugates (*14*, *15*). More recently, Steuer *et al.* (*16*) reported additional conjugated GHB metabolites, including GHB-glycine, GHB-glutamate, GHB-taurine, GHB-carnitine, and a feature they tentatively assigned as a GHB-derived pentose. These adducts were detected in urine but not serum. Subsequently, they found that the tentative GHB-pentose and GHB-glycine were promising biomarkers as they were up-regulated in urine 28 hours after GHB intake in a trial with sampling at 4.5, 8, 11, and 28 hours (*17*).

We recently conducted a double-blinded placebo controlled clinical trial, specifically designed to improve methods for detection of GHB (*6*, *18*). The trial is unique as it included regular sampling of both urine and blood from baseline to 120 hours after intake of GHB or placebo in 30 participants (Fig. 1A). Previous results from this trial showed that sampling blood in fluoride/oxalate tubes and cooling within 12 hours may permit lowering the GHB cutoff value in blood (*6*) and that this could prolong the detection window of GHB by at least 2 hours (*18*). This is, however, unlikely to increase the detection rate in DFSA cases considerably due to their late presentation. Furthermore, it was confirmed that the tentative GHB-pentose, but not GHB-glycine, was a promising biomarker in urine using a semiquantitative method (*19*). However, the specific structure of the GHB-pentose was unknown, which prevented quantitative concentration measurements and validation of the metabolite as a biomarker.

**Fig. 1.**
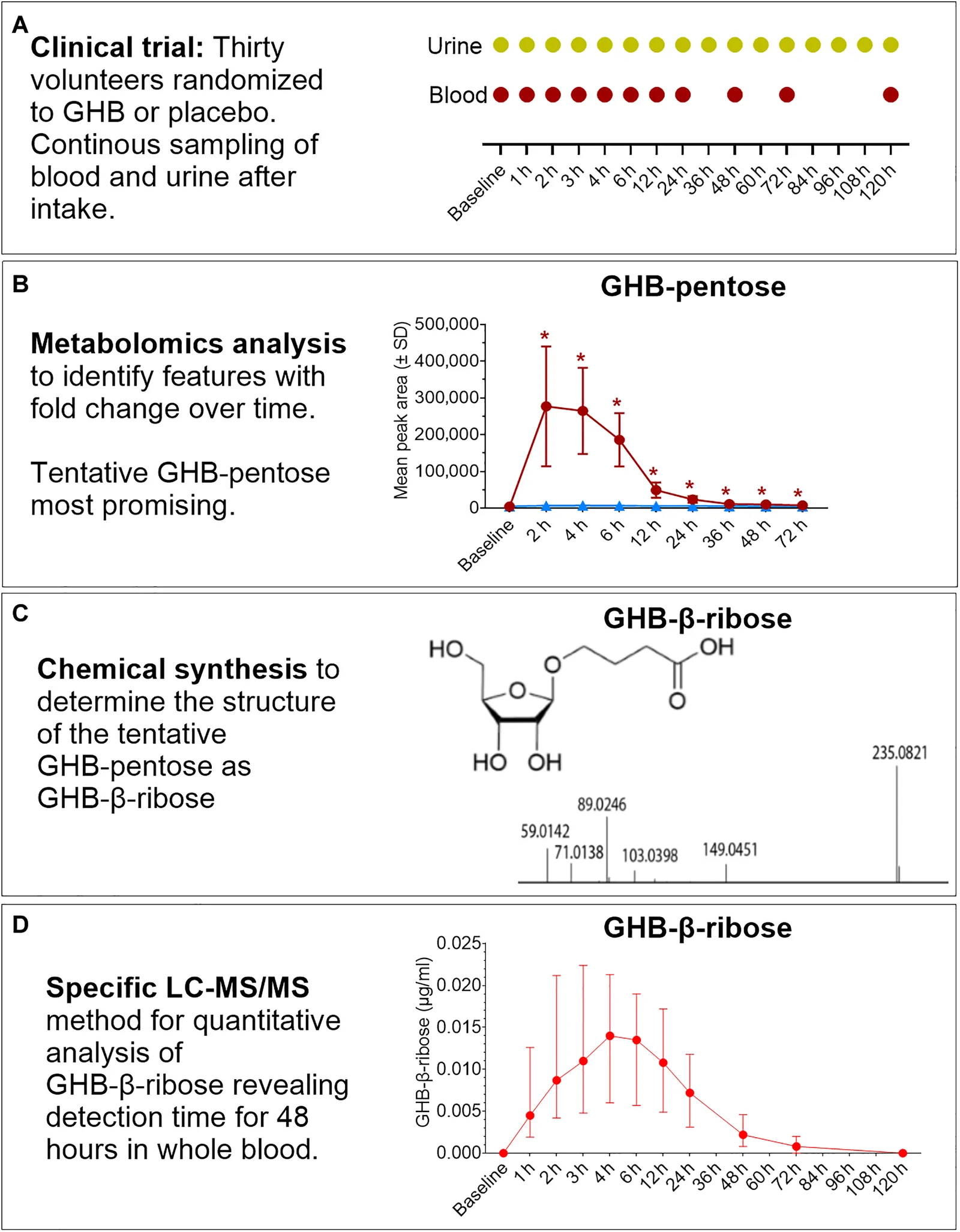
Overview of the study. (**A**) The clinical trial included 30 healthy volunteers randomized to either GHB in the form of sodium oxybate (50 mg/kg body weight capped at 4.5 g) or placebo. We collected urine and blood at times after intake as indicated by circles. At 36, 60, 84, 96, and 108 hours (h), the participant collected urine at home. (**B**) Metabolomics analysis was applied to blood samples (collected at baseline, 4, 6, 12, 24, and 48 hours) and urine samples (collected at baseline, 2, 4, 6, 12, 24, 36, 48, and 72 hours). Features significantly up-regulated or down-regulated at 2 to 6 hours were selected, and intensity over time was inspected to identify features persistently regulated. The tentatively annotated GHB-pentose was the most promising feature. (**C**) The precise structure of the tentative GHB-pentose was determined by chemical synthesis as GHB-β-ribose. (**D**) A sensitive LC-MS/MS analysis was developed, and GHB-β-ribose was detectable in blood for 48 hours after intake.

Here, we present results from the clinical trial (*6*, *18*, *19*) (Fig. 1A) where we used untargeted metabolomics to globally analyze metabolites consistently up-regulated in samples after GHB intake (Fig. 1B). The most promising feature corresponded to the previously suggested GHB-pentose, and through chemical synthesis, we managed to lastly establish that the suggested pentose is GHB-β-ribose, a β-anomeric ribose conjugate in which GHB is attached through its hydroxy group to the ribose anomeric carbon (Fig. 1C). This allowed us to develop an ultrahigh-performance liquid chromatography–tandem mass spectrometry (UHPLC-MS/MS) method for quantification of GHB-β-ribose and confirm that the biomarker could extend the detection window of GHB up to 48 hours postconsumption in blood (Fig. 1D) and more than 24 hours in urine.

## RESULTS

### The clinical trial

We included 30 healthy volunteers, 14 males, and 16 females with a median age of 26 [range: 19 to 64] years, mean height ± SD of 174 ± 10 cm, and mean weight ± SD of 76 ± 19 kg. Less than three smoked. They were randomized 1:1 to either sodium oxybate (50 mg/kg), the salt of GHB, or placebo. Samples were taken up to 5 days after intake of the study medication at times as illustrated in Fig. 1A. The participants took urine samples at home 36, 60, 84, 96, and 108 hours after intake. Five participants collected one of their samples too late at home (1.05 to 4.5 hours after the scheduled time). The study is described in detail earlier (*18*).

### Untargeted metabolomics: PCA reveals clustering patterns after GHB intake

Whole blood (collected at baseline, 4, 6, 12, 24, and 48 hours) and urine (collected at baseline, 2, 4, 6, 12, 24, 36, 48, and 72 hours) samples were subjected to liquid chromatography–high-resolution time-of-flight mass spectrometry (LC-HR-TOF-MS) for untargeted metabolomics analysis. We detected 1585 and 577 features in whole blood samples in positive and negative electrospray ionization (ESI) mode, respectively, and 4817 and 1711 features in urine in positive and negative ESI mode, respectively, following preprocessing. After normalization, no clustering by batch was observed in whole blood or urine samples in either ESI mode [see representative principal components analysis (PCA) scores plots in fig. S1].

Quality control (QC) samples in urine were clustered across several component combinations, although with a more linear distribution in later components (see representative PCA scores plots in fig. S2). In whole blood, some of the QC samples appeared distributed along a trajectory in PC1 versus PC2 and PC3 versus PC4 in both positive and negative ESI mode, indicating small drifts in the data (see representative PCA scores plots in fig. S3).

Individual PCA score plots of whole blood samples from the GHB group collected 4 and 6 hours postdose showed only minor separation from baseline at PC3 in positive ESI mode not in negative mode (figs. S4 and S5). In contrast, 2 and 6 hours postdose urine samples separated from baseline in both ESI modes (figs. S6 and S7) with separation persisting after exclusion of GHB and GHB fragments (fig. S6, B and D). The strongest separation occurred at 6 hours (separation at PC1 with explained variance > 20% in both ESI modes), exceeding that at 2 hours (separation at PC2 with explained variance < 15.0% in both ESI modes). No clustering was evident at 24 hours (figs. S6 and S7).

### Identification of GHB-pentose and potential other GHB biomarkers with extended detection window

We selected features with significant fold change (fc) at 2, 4, and 6 hours in urine and 4 and 6 hours in whole blood, along with features matching previously reported metabolites, including GHB and known GHB metabolites (e.g., GHB-glutamate, GHB-carnitine, GHB-glycine, GHB-pentose, GHB-taurine, DHB, 3,4-DHB, glycolic acid, succinic acid, and succinyl carnitine) (*19*) (table S1). The features were subjected to annotation and identification based on the Metabolomics Standards Initiative (*20*) levels. We inspected the intensity-time profiles of each feature to evaluate how long they were significantly regulated in the GHB group compared to placebo. Figures 2 and 3 shows the time curves for the most interesting, identified features.

**Fig. 2.**
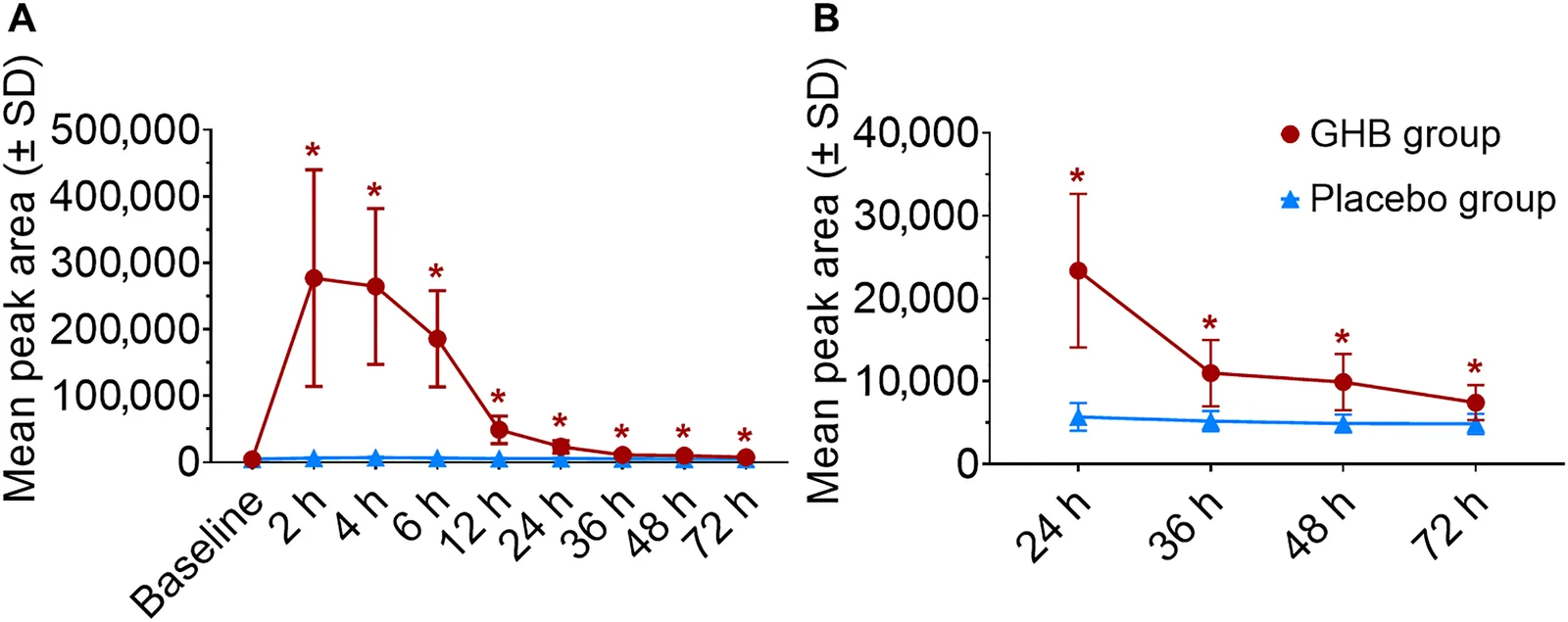
Time profile of a tentatively identified GHB-pentose conjugate in urine following GHB administration. (**A**) Mean peak area intensities (± SD) of GHB-pentose (later identified as GHB-β-ribose) in urine over a 72-hour period after administration in the GHB (*n* = 15) and placebo (*n* = 15) groups. (**B**) Zoom in on (A) from 24 hours after intake. Note the nonlinear *x* axis. Significant (*P* < 0.05, two-sample *t* tests) fc differences between treatment groups are marked with *.

**Fig. 3.**
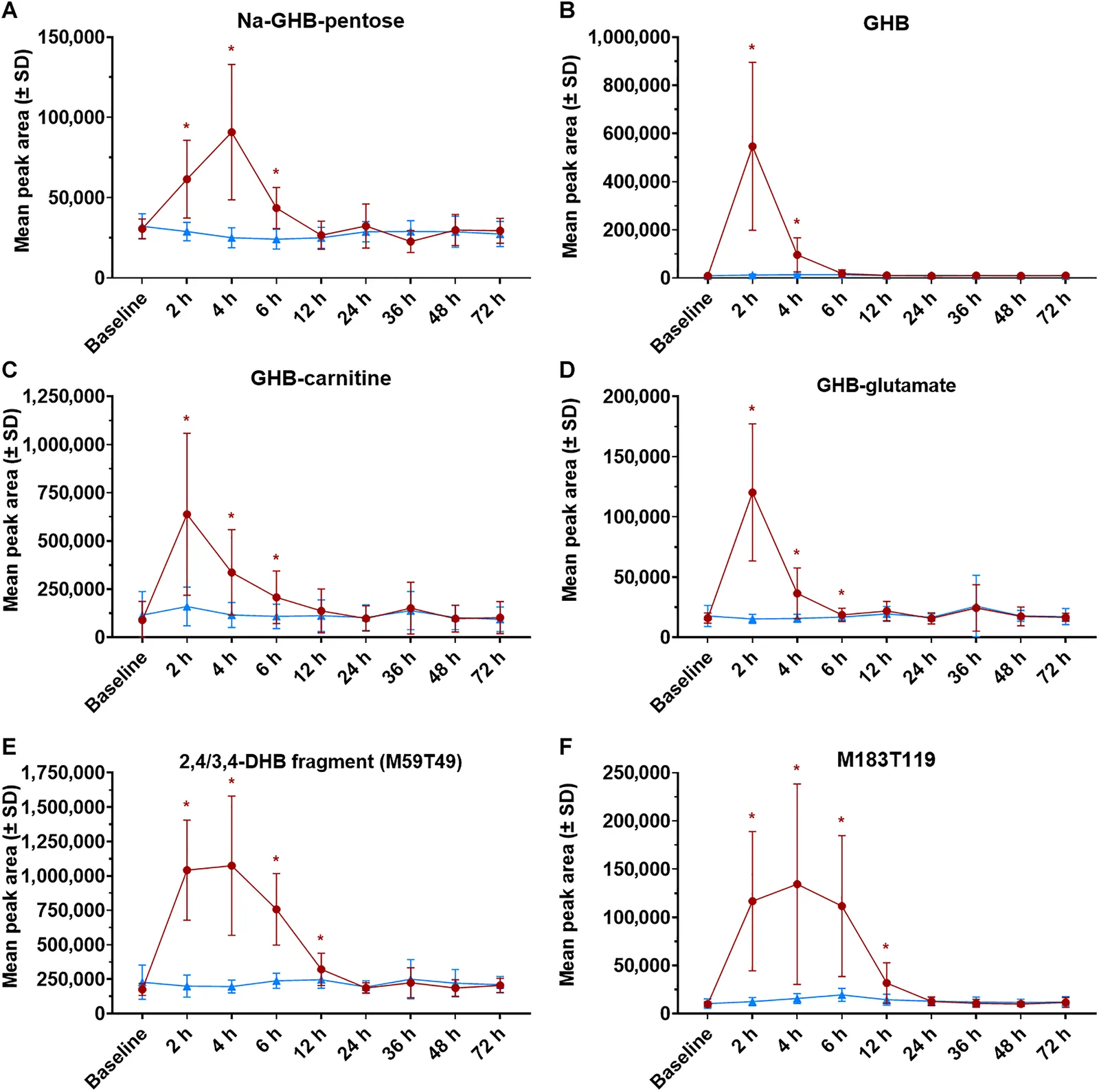
Time-dependent urinary profiles of metabolomics-derived features associated with GHB administration. (**A**) Na-GHB-pentose, (**B**) GHB, (**C**) GHB-carnitine, (**D**) GHB-glutamate, (**E**) 2,4/3,4-DHB-fragment (MT59T49), and (**F**) M183T119. Mean peak area intensities (± SD) in the GHB (red circles; *n* = 15) and placebo (blue triangles; *n* = 15) groups are shown over a 72-hour period after administration. Note the nonlinear *x* axis. Significant (*P* < 0.05, two-sample *t* tests) fc differences between treatment groups are marked with *.

The most promising feature, M235T207, was identified in urine in negative ESI mode and exhibited significant fc for at least 72 hours (fc = 1.53, *P* < 0.001; Fig. 2, A and B). The mass corresponded to the GHB-pentose previously described. A likely sodium adduct of this compound (M259T206) was also observed in urine in positive ESI mode with a significantly increased fc for 2 to 6 hours (fc at 6 hours = 1.83, *P* < 0.001; Fig. 3A). GHB itself was found up-regulated for up to 4 hours in urine (Fig. 3B).

Among GHB–amino acid conjugates, GHB-glycine was detected in urine but was not significantly up-regulated or down-regulated. GHB-carnitine was also detected in urine with a significant fc for up to 6 hours (fc = 1.93, *P* = 0.018; Fig. 3C). GHB-glutamate showed a significant fc for 2 to 4 hours in urine (fc at 4 hours = 2.32, *P* = 0.0011; Fig. 3D). Glycolic acid was not significant in urine or in the whole blood analysis at 4 hours or later. In urine, an in-source fragment of 2,4-DHB or 3,4-DHB, M59T49 (C_2_H_3_O_2_), was promising and was significantly elevated at 2 to 12 hours (Fig. 3E), whereas the precursor, however, did not show any significant fcs. The untargeted analysis yielded several unidentified features with potential relevance (table S1). Among these, M183T119 showed significantly fc at 2 to 12 hours (Fig. 3F).

### GHB-β-ribose is a human metabolite derived from GHB

On the basis of the promising intensity-time profile of the M235T207 (tentative GHB-pentose) feature, we decided to investigate whether it was directly derived from GHB. By observing main transitions in the MS/MS analysis to 103 and 149, we could confirm that the feature fragmented to masses that corresponded with GHB and a pentose (Fig. 4A). Furthermore, in this and a prior study (*21*), we observed a feature (M259T206) in positive ESI mode coeluting with M235T207, with a mass corresponding to the sodium adduct of M235T207, indicating that the structure is a highly hydroxylated compound as these are known to easily complex cations. Last, we observed that M235T207 was most easily detected in negative ESI mode as well as strongly retained on an amine-based solid-phase cartridge. Data that indicated that M235T207 have a free carboxylic group (Fig. 4A).

**Fig. 4.**
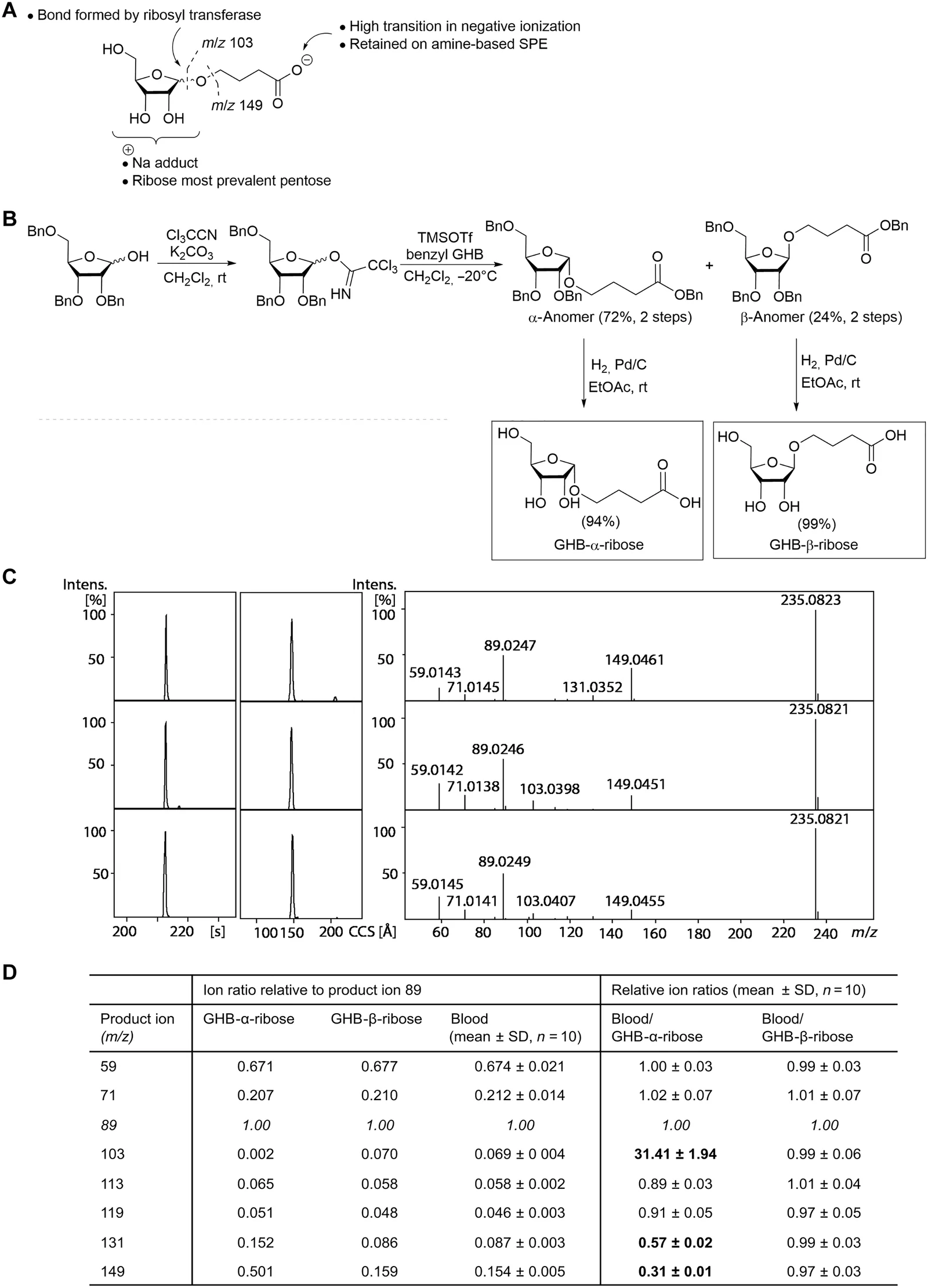
Identification of GHB-β-ribose. (**A**) Considerations for structure elucidation. (**B**) Chemical synthesis of anomeric adducts between GHB and ribose. rt, room temperature. (**C**) Extracted ion chromatograms (*m/z* 235.082; left), mobilograms (middle), and MS/MS-spectra (right) from LC-TIMS-TOF-MS analysis of GHB-α-ribose, GHB-β-ribose, and an authentic forensic blood sample positive for GHB. The collision cross section (CCS) values for GHB-α-ribose, GHB-β-ribose, and the feature in blood are 147.9, 147.3, and 148.5, respectively. The deviation between CCS values of GHB-α-ribose and blood is 0.4% versus 0.8% for GHB-β-ribose and blood, both of which are within normal instrument variation indicating both standard compounds fit the blood feature. (**D**) Relative ion ratios from a corresponding LC-MS/MS analysis of GHB-α-ribose, GHB-β-ribose, and 10 authentic forensic blood samples positive for GHB displaying greater similarity between GHB-β-ribose and blood than GHB-α-ribose (differences are marked in bold in the table).

Ribose is the predominant pentose in biological systems and a central component of nucleosides, nucleotides, and key metabolic cofactors such as ATP and NAD(H). This argued that the unknown pentose likely would be a ribose. ADP-ribosylations are well known biochemical transformations in nature catalyzed by ADP-ribosyl transferases (ARTDs), and although the targets usually are protein side chains as serine (hydroxyl group modification) and aspartate (carboxylic acid modification), it has also been observed that ARTDs may accept small molecules as substrates (*22*, *23*).

If catalyzed by ARTDs, ribosylation of GHB could then principally occur at both the free hydroxyl as well as the carboxylic acid end of GHB. However, on the basis of the observation that the adduct likely contained a free carboxylic acid, we concluded that the unknown metabolite most likely corresponds to GHB bound through the gamma-hydroxyl group (Fig. 4A). The last question was if the stereochemistry at the anomeric center of ribose would then be the α- or β-isomer. Ultimately, we designed a synthesis of both isomers that we termed GHB-α-ribose and GHB-β-ribose. Starting from commercially available benzyl-protected ribose, the two target compounds were subsequently obtained by initial trichloroacetimidate activation of the anomeric hydroxyl group, followed by a high-yielding coupling to benzylated GHB and equally efficient palladium catalyzed debenzylation [Fig. 4B; the detailed synthesis and characterization with nuclear magnetic resonance (NMR) spectroscopy spectra are in the Supplementary Materials]. Following LC-trapped ion mobility spectrometry (TIMS)–TOF-MS and LC-MS/MS analysis and comparison to authentic forensic GHB-positive whole blood samples, we lastly observed that, although the α- and β-anomer elute closely, only the β-anomer had exactly the same retention time. Furthermore, the distinct intensities of all the eight product ion transitions that we observed for feature M235T207 in our biological samples fitted the β-anomer, whereas the α-anomer showed marked different intensities for product ions 103, 131, and 149 (Fig. 4, C and D).

### GHB-β-ribose verified as a promising biomarker by specific quantitative analysis

Using the synthesized GHB-β-ribose, we established a quantitative LC-MS/MS method to evaluate the concentrations in whole blood and urine from our clinical study and from 100 anonymized authentic “GHB-blank” clinical forensic cases (mass spectrometric conditions in table S2). The validated limit of detection (LOD) was 0.001 μg/ml for both blood and urine. The lower limit of quantification (LLOQ) was correspondingly 0.001 μg/ml for both matrices. The upper limit of quantification (ULOQ) was 40 μg/ml. Figure S8 shows chromatograms of GHB-β-ribose in blood and urine from a blank authentic case and a positive case from the clinical study 24 hours after GHB intake.

The validated relative intralaboratory reproducibility SDs (RSD_R,intralab_ values) for whole blood were 8 and 7% at GHB-β-ribose concentrations of 0.005 and 0.1 μg/ml, respectively. The corresponding trueness expressed as the relative bias were 13 and 3%, respectively. Similarly, the RSD_R,intralab_ values for urine were 7, 9, and 12% whereas the biases were 7, 0, and −5% at concentrations of 0.1, 1, and 10 μg/ml, respectively. The relative repeatability SDs (RSD_r_ values) were, in all cases, less than 5%.

The extraction recoveries from whole blood samples were 83 ± 4% (mean ± SD) at 0.005 μg/ml and 82 ± 4% at 0.1 μg/ml. For urine samples, the extraction recoveries were 95 ± 8%, 90 ± 5%, and 85 ± 6% at concentrations of 0.1, 1, and 10 μg/ml, respectively. The matrix effects were 51 ± 4% and 51 ± 3% for GHB-β-ribose in whole blood at concentrations of 0.005 and 0.1 μg/ml, respectively. For urine, the matrix effects were 80 ± 2%, 77 ± 3%, and 65 ± 4% at concentrations of 0.1, 1, and 10 μg/ml, respectively. The matrix effects related to the internal standard (IS) were 35 ± 7% for whole blood and 61 ± 8% for urine.

GHB-β-ribose was not detected in any whole blood sample from the placebo group or 100 anonymized authentic “GHB-blank” cases (Table 1). The median concentration of GHB-β-ribose in urine was 0.0062 μg/ml (range: not detected to 0.089 μg/ml) in the placebo group and 0.0070 μg/ml (range: not detected to 0.10 μg/ml) in the authentic samples (Table 1). On the basis of these results, cutoff values of GHB-β-ribose [0.001 μg/ml (LOD) and 0.12 μg/ml] were determined in blood and urine, respectively.

**Table 1.** Endogenous levels and cutoff values of GHB-β-ribose in the placebo group and in authentic “GHB-blank” cases measured in blood and urine. n.d.: not detected.

	Blood		Urine	
	Placebo group	Authentic cases	Placebo group	Authentic cases
Number	165	100	240	100
GHB-β-ribose				
Median (μg/ml)	n.d.	n.d.	0.0062	0.0070
Min (μg/ml)	n.d.	n.d.	n.d.	n.d.
Max (μg/ml)	n.d.	n.d.	0.089	0.103
Cutoff (μg/ml)*	0.001 (LOD)	0.001 (LOD)	0.13	0.12

* Cutoff values are calculated as described in Materials and Methods. The GHB concentrations were maximally 0.20 and 1.7 μg/ml in blood and urine, respectively, in “GHB-blank” cases.

Using these cutoff values, GHB-β-ribose originating from GHB intake could be distinguished from endogenous concentrations in whole blood in all 15 participants (100%) in the GHB-treated group up to 24 hours after intake (Fig. 5A) and in 13 participants (87%) after 48 hours. In urine, GHB-β-ribose concentrations were higher than cutoff after GHB intake in all participants up to 24 hours after intake (Fig. 5B), although one value were below cutoff at 12 hours. Ten (67%) and 8 (53%) participants from the GHB group had GHB-β-ribose levels above the cutoff value in urine after 24 and 48 hours, respectively. By visual inspection, the range of GHB-β-ribose concentrations was not larger in samples taken at home than in those collected in the trial facility in the placebo group (Fig. 5B).

**Fig. 5.**
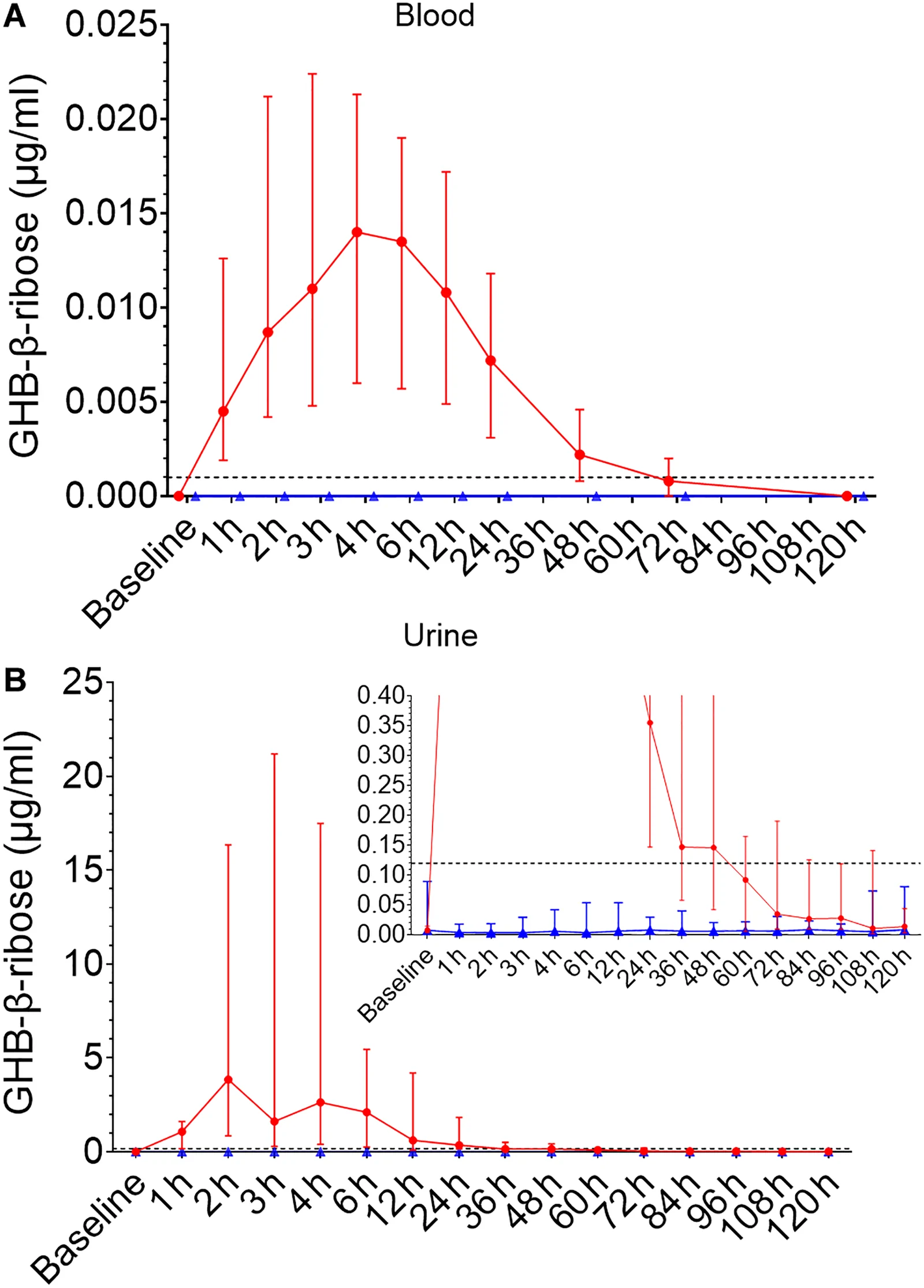
Concentrations of GHB-β-ribose in blood and urine support an extended detection window following GHB administration. Median and range of GHB-β-ribose concentrations over time in GHB-treated participants (*n* = 15) and the placebo group (*n* = 15) in the clinical trial in (**A**) blood and (**B**) urine. The inset in (B) zooms in on the low range of concentrations to illustrate cutoff (dotted line) and the concentration range in the placebo group. Note the nonlinear *x* axis. Dotted lines display suggested cutoffs based on the analysis of 100 authentic cases (LOD values of 0.001 μg/ml in blood and 0.12 μg/ml in urine). Values < LOD were assigned a value of 0 μg/ml.

In whole blood samples from 10 anonymized driving under the influence of drug cases with a median GHB concentration of 28 μg/ml (range: 6 to 167 μg/ml), the median GHB-β-ribose concentration was 0.22 μg/ml (range: 0.006 to 1.01 μg/ml).

## DISCUSSION

This study presents the structure of GHB-β-ribose, a biomarker for GHB intake with the potential to substantially reduce the number of false negatives in DFSA cases and provide an evidence-based understanding of GHB’s role in DFSAs.

GHB-β-ribose was up-regulated in blood and urine from our clinical trial specifically designed to investigate long-term biomarkers of GHB intake. The chemical structure was elucidated by synthesis of two possible isomers and verified by NMR, LC-TIMS-TOF-MS and LC-MS/MS analysis, revealing the structure of GHB-β-ribose previously tentatively identified as GHB-pentose (*16*, *17*, *19*). Furthermore, this study provides the quantification of GHB-β-ribose at endogenous levels and after controlled GHB intake, introducing GHB-β-ribose as a long-awaited biomarker, potentially extending the detection window of GHB intake to 48 hours after intake in blood and 24 hours in urine.

Typically, drugs and their metabolites display a longer detection window in urine than in blood due to accumulation. In accordance, the maximum GHB-β-ribose concentration in the GHB group in blood was several hundred times lower than the maximum GHB-β-ribose concentration in urine. This may explain why the metabolomics analysis did not detect GHB-β-ribose in blood as a relevant feature. Still, the quantitative analysis of GHB-β-ribose showed that blood was better suited to distinguish GHB intake from endogenous concentrations, mainly because of a lower intra- and interindividual variation. Thus, the practical cutoff value, which is mandatory for endogenous compounds, had to be set substantially higher in urine than in blood, reducing the detection window in urine. Hence, although LC-HR-TOF-MS analysis is a good screening method for potentially relevant features, the present study underlines that measurements in both blood and urine, structural characterization, and accurate quantitative methods are essential to establish the utility of potential biomarkers.

In agreement with previous findings (*16*, *17*), our metabolomics analysis identified a greater number of GHB-regulated metabolites in urine than in blood. GHB-glycine was not identified as significantly regulated by GHB intake in urine, although a targeted analysis of the same samples showed increased concentrations of GHB-glycine in urine up to 6 hours after intake (*19*). GHB-glutamate was significantly increased in the metabolomics analysis for 4 hours in urine, which corresponded with the results in the targeted analysis (*19*). In addition, we confirmed up-regulation of GHB-carnitine as in previous studies (*16*, *17*, *21*), but the use of this metabolite was previously abandoned due to instability in samples stored at −20°C (*24*). Furthermore, an in-source fragment of 2,4-DHB or 3,4-DHB (C_2_H_3_O_2_), was significantly increased up to 12 hours in urine, and the structurally uncharacterized metabolites (unknowns) were not regulated significantly at 12 hours after GHB intake, except for M183T119, which was significantly elevated in urine in the GHB group for at least 12 hours. This feature corresponded in mass/charge ratio (*m/z*) to compound U8 reported by Steuer *et al.* (*16*), which exhibited a twofold change at 4.5 hours and was tentatively assigned C_5_H_2_N_4_O_2_S. Thus, none of the other metabolites identified in the metabolomics analysis were as promising as GHB-β-ribose. However, as GHB-β-ribose turned out to be more useful in blood than in urine using the targeted analysis, it could be interesting to investigate the concentration profile of, e.g., GHB-glycine and M183T119 after GHB intake with a targeted analysis in blood.

Key strengths of this study were the collaboration among staff with expertise in chemistry, analytical methods and clinical practice, respectively, and the placebo-controlled, randomized, clinical study. The trial was randomized to diminish individual confounding factors and planned to enable detection of metabolites early after GHB intake, where we expected them to separate most significantly from the placebo group. The subsequent regular sampling intervals made it possible to monitor the temporal profiles of potential biomarkers and the duration of elevation above endogenous concentrations—a crucial tool to select biomarkers for quantification. Nonetheless, the study had several limitations. The sensitivity of the untargeted metabolomics approach is inherently lower than that of targeted methods applying extraction, as illustrated with GHB-β-ribose and GHB-glycine. Thus, the sensitivity of the metabolomics analysis may not be sufficient to detect all relevant metabolites. Furthermore, the study was limited to a single, weight-adjusted dose of sodium oxybate administered to 15 selectively recruited healthy volunteers. The dose corresponded to the maximum approved dose for sodium oxybate and was sufficient to induce pronounced sedative effects in all participants (*18*) but does not capture the variability of doses applied in DFSA cases. However, the potential of GHB-β-ribose as a biomarker is supported by the detection of GHB-β-ribose in GHB-positive authentic cases, which likely represent a broader range of doses and intake of illicit GHB formulations.

Ethanol is a frequent finding in DFSA cases (*7*) and simultaneous consumption of ethanol may reduce the metabolism and elimination of GHB. A clinical study (*25*) reported a modest increase in peak GHB concentration and half-life in volunteers ingesting both GHB and ethanol (0.6 g/kg). Although this would tend to increase the detection time of GHB and GHB-β-ribose, ethanol consumption could theoretically increase the endogenous concentrations of GHB and GHB-β-ribose, which could necessitate a higher cutoff value of GHB-β-ribose. Although ethanol could be present in some of the authentic samples in this study, we did not examine this. Moreover, in the clinical study we excluded use of drugs that could interact with the metabolism of GHB, and we did not control storage and transportation temperature in urine samples taken at home. The endogenous levels in the placebo group did not seem to differ between sampling in the facility and at home, but we cannot exclude that different storage conditions may lead to formation or degradation of GHB-β-ribose after sampling in this study and in general. Overall, the cutoff values and detection window suggested in the present study does not necessarily apply to populations with different demographic characteristics, individuals exposed to other doses of GHB, individuals with recent or regular ethanol use, or use of other drugs. In addition, they may not apply to samples collected under conditions different from those in the present study or to plasma or serum samples.

The cutoff values in whole blood corresponded to the LOD of the method suggesting a lower LOD is needed to determine the endogenous levels of GHB-β-ribose. However, several concentrations in the blood samples from the placebo group and the 100 authentic “blank” cases fulfilled the quantifier/qualifier ion ratio criteria but the concentration were just below the LOD and thus not reported. This indicates that the endogenous levels are most likely in the proximity to the LOD of the method, and a higher sensitivity might not increase the detection window. Last, the presented quantitative data were obtained using GHB-^13^C_4_ as IS, which eluted ∼0.14 min later than GHB-β-ribose. Although they elute closely, and the samples were cleaned up selectively by anion exchange solid-phase extraction (SPE), and the remaining mean matrix effect was removed by the applied data processing technique, the variation in matrix effects within each sample class may introduce uncertainties and may partly explain the higher variation in urine compared to blood. Application of a stable isotope labeled analog of GHB-β-ribose would probably reduce the impact of this variation on analytical results.

We encourage other forensic toxicologists to analyze for GHB-β-ribose in their casework to add knowledge about the concentrations of this compound in both GHB-positive and GHB-negative cases to increase the basis for determination of the cutoff values. Future clinical studies investigating different GHB doses, additional populations, as well as GHB combined with ethanol are warranted to improve understanding of the dose-response effects, demographics, and interactions with ethanol and other drugs on GHB-β-ribose concentrations. In addition, studies are needed to determine the potential for postsampling formation and degradation of the biomarker under different sampling and storage conditions.

In conclusion, on the basis of our clinical trial specifically designed for biomarker identification, metabolomics analysis, chemical synthesis, and the development of a targeted LC-MS/MS analysis, we present the identification of GHB-β-ribose as the hitherto most promising biomarker for GHB exposure detectable for 48 hours in blood and 24 hours in urine. This biomarker represents a long-awaited advancement that can strengthen the legal rights of DFSA victims and improve our ability to determine the prevalence of proactive DFSAs, as well as clarify the role of GHB in such cases.

## MATERIALS AND METHODS

### Clinical trial and authentic samples

The clinical trial is described in detail previously (*18*). Briefly, 30 healthy volunteers were randomized 1:1 to treatment with GHB [sodium oxybate (50 mg/kg), capped at 4.5 g] or placebo. Samples of whole blood [Vacuette tube (no. 454297) containing a mixture of sodium fluoride (NaF) and potassium oxalate (FX tube)] and urine (no additives) were collected at baseline and 1, 2, 3, 4, 6, 12, 24, 48, 72, and 120 hours after administration of the trial medication. The participants also collected urine at home after 36, 60, 84, 96, and 108 hours (Fig. 1A). They were asked to collect the sample as close to the given time as possible and note the time for collection. Up to 1 hour before or after the scheduled time was acceptable. Apart for fasting for 7 hours before intake of the study medication, no restrictions or rules for, e.g., eating, sleeping, or exercise were applied in relation to the time for sampling. Samples collected at the trial location were placed on ice immediately after collection and frozen at −70°C within 7 hours. Urine samples collected at home were refrigerated and transported to the trial location in cooling boxes within 38 hours, where they were frozen at −70°C. Samples from the clinical trial were stored at −70°C until analysis (up to 37 months). All study participants understood and were able to read Danish and provided written informed consent.

One hundred authentic “GHB-blank” clinical forensic cases sent for storage at Department of Forensic Medicine in Aarhus, Denmark, in which there were no suspicion of drug related crimes or GHB intake, were selected and anonymized before determination of endogenous levels of GHB and GHB-β-ribose in whole blood and urine. Both were sampled in FX tubes under routine conditions and stored at −20°C within 12 hours after sampling until analysis. Females constituted 62 cases with a median age of 26 years (range: 13 to 76 years) and males 38 cases with a median age of 30 years (range: 15 to 55 years). Forty-two of the 100 cases were analyzed after a median of 54 days (range: 22 to 103 days) and 58 cases were analyzed after a median of 317 days after sampling (range: 139 to 384 days). The GHB concentrations were maximally 0.20 and 1.7 μg/ml in blood and urine, respectively.

Ten authentic driving under the influence of drugs cases with a concentration of GHB > 5 μg/ml were selected and anonymized for verification of GHB-β-ribose in whole blood. The samples were stored for a median of 8 months (range: 4 to 68 months) at −20°C before analysis.

### Chemicals and reagents

Chemicals for LC-MS were obtained from Merck (Darmstadt, Germany): acetonitrile (ACN; LiChrosolv hypergrade, LC-MS), hydrochloric acid (37%), methanol (MeOH; LC-MS grade), and formic acid (98 to 100%, LC-MS grade). Water was purified using a Milli-Q IQ 7000 system (Millipore, Burlington, MA, USA). The IS mixture comprised amphetamine-d_5_, benzoylecgonine-d_3_, cocaine-d_3_, cotinine-d_3_, diazepam-d_5_ (all 0.1 mg/ml, Cerilliant), and paracetamol-d_4_ (1.0 mg/ml, Chiron, Trondheim, Norway). Analytical reference standards included sodium γ-hydroxybutyrate (Cerilliant), *N*-hydroxybutanoyl-l-glycine and (4-hydroxybutanoyl)-l-phenylalanine (PharmaResources, Shanghai, China), GHB-carnitine (Toronto Research Chemicals, Toronto, Canada), glycolic acid, succinic acid, *O*-succinyl-l-carnitine, (*S*)-3,4-dihydroxybutyric acid, and (±)-DHB (Sigma-Aldrich, St. Louis, MO, USA). GHB-glutamate was synthesized in-house (*21*).

### Sample preparation for LC-HR-TOF-MS untargeted metabolomics analysis

Whole blood (baseline, 4, 6, 12, 24, and 48 hours) and urine (baseline, 2, 4, 6, 12, 24, 36, 48, and 72 hours) samples were thawed at room temperature and gently mixed before analysis. Distinct sample preparation protocols were applied for each matrix.

For whole blood, 300-μl aliquots were transferred to a 96-deep-well plate using a Tecan FREEDOM EVO 200 (Tecan, Männedorf, Switzerland). Each well received 150 μl of 0.1 M HCl and was mixed for 60 s at 1000 rpm (MixMate 5353, Eppendorf, Hamburg, Germany). Subsequently, 200 μl of IS solution in MeOH was added and mixed for 60 s at 100 rpm. Protein precipitation was achieved by adding ACN in three 100-μl portions with mixing, followed by an additional 300 μl of ACN and gentle mixing. After standing 15 min at room temperature, plates were centrifuged at 5000*g* for 5 min (Rotanta 460, Hettich, Kirchlengern, Germany). Supernatants (600 μl) were transferred to an AcroPrep Advance 96-well filtration plate (30-kDa Omega membrane, Pall Corporation, Port Washington, NY, USA) and centrifuged at 2000*g* for 15 min, and 500 μl of the filtrate was collected into 96-well plates with Chromacol glass inserts (Thermo Fisher Scientific, Waltham, MA, USA). Samples were evaporated to dryness at 40°C for 45 to 60 min (MiniVap, Porvair Sciences, King’s Lynn, UK), stored at −70°C, and reconstituted before analysis in 200 μl of 15% MeOH/0.1% formaldehyde (FA) in water, mixed at 1650 rpm for 30 s, and centrifuged at 5000*g* for 5 min.

For urine, 200-μl aliquots were transferred to a 96-deep-well plate, and 100 μl of a solution containing 5% ACN/0.1% FA in water was added. The plate was centrifuged at 10,000*g* for 30 min at room temperature. The supernatant (50 μl) was then diluted with 250 μl of the same solution, followed by centrifugation at 10,000*g* for 15 min. No IS was added to the urine samples.

### LC-HR-TOF-MS untargeted metabolomics analysis

Chromatographic separation was performed using an ACQUITY I-Class UPLC system (Waters Corporation, Milford, MA, USA) equipped with an ACQUITY HSS T3 C18 column (100 mm by 2.1 mm, 1.8 μm, Waters). Mobile phase A consisted of 0.1% FA (v/v) in water, and mobile phase B consisted of 0.1% FA (v/v) in a 1:1 (v/v) mixture of MeOH and ACN. Chromatographic separation was performed at a flow rate of 0.4 ml/min with the column maintained at 50°C. The gradient elution program was as follows: 0% B (0 to 2 min), 0 to 40% B (2 to 6 min), 40 to 60% B (6 to 6.5 min), 60 to 80% B (6.5 to 11 min), 88 to 100% B (11 to 11.5 min), 100% B (11.5 to 18 min), 100 to 0% B (18 to 19.5 min), and 0% B (19.5 to 21 min). Samples were stored in the autosampler at 6°C, and the injection volume was 6 μl for urine in positive ESI mode and 5 μl for all other analyses.

Mass spectrometric detection was carried out on a Bruker maXis Impact quadrupole time-of-flight (QTOF) mass spectrometer (Bruker Daltonics, Bremen, Germany) operated in positive and negative ESI mode. The capillary voltage was set to 4000 V for positive ESI and 2500 V for negative ESI. The nebulizer pressure was 1.2 bar, with a drying gas flow of 11.0 liters/min at 220°C. Data were acquired over a mass range of *m/z* 50 to 1000 at a sampling rate of 4 Hz. Auto-MS/MS (data-dependent acquisition) was used for fragmentation of a few selected samples, using collision energies of 10, 20, and 30 eV and a sampling rate of 10 Hz to facilitate molecular feature annotation and identification.

Internal calibration was performed at the end of each run using sodium formate in both ionization modes. Four QC samples, prepared by pooling equal volumes of all whole blood or urine samples, were injected at the beginning of each batch. During sample analysis, injections were randomized, and a QC sample was included after every 10th sample to monitor instrument performance throughout the batch. The same pooled QC sample was used across all batches within each matrix. In addition, four samples within a batch were injected twice and four samples from a former batch were injected with a new batch.

### LC-HR-TOF-MS untargeted metabolomics data processing and normalization

Raw data from positive and negative ESI modes were converted to mzML using MSConvertGUI (version 3, ProteoWizard, Palo Alto, CA, USA) with vendor-specific MS1 peak picking. Scans outside 20 to 1000 s were excluded to remove early and late artifacts. Data processing was performed in RStudio (version 2024.09.1, PBC, Boston, MA, USA) with XCMS (*26*). Peaks were detected using centWave (*27*) with a mass accuracy of 12 parts per million (ppm) and signal-to-noise (S/N) ratio ≥ 10, aligned by loess regression, and grouped by density-based clustering (bandwidth of 2.5 s). Features present in ≥90% of samples from each treatment group at a given time point (e.g., all 1-hour samples in the GHB group) were retained for urine data, whereas a lower threshold (≥50%) was applied for blood to increase sensitivity for feature detection in this matrix, where fewer consistently detected features were observed.

Replicability was evaluated on the basis of the pooled QC samples, where features with coefficients of variation (CVs) below 30% were considered analytically stable. However, no strict filtering based on this CV threshold was applied, as to retain potentially relevant biomarkers. For features considered biologically relevant, replicability was however evaluated by inspection of their consistency across replicate injections and QC samples.

Feature annotation, including identification of isotopic patterns and adduct forms, was carried out using CAMERA (*28*). The final feature table, comprising aligned retention times and *m/z* values, was exported for further analysis. Data were subsequently batch-corrected feature-wise and normalized to total feature intensity in Microsoft Excel (version 2504, Microsoft Corporation, Redmond, WA, USA).

### Statistical metabolomics analysis and feature selection

PCA with Pareto scaling was performed in SIMCA (version 18, Umetrics, Umeå, Sweden). Fc were calculated in Microsoft Excel as mean intensity ratios between treatment groups at 2 (urine only), 4, 6, and 12 hours postdose. Variance equality was tested with an *F* test, followed by two-sample *t* tests (assuming equal or unequal variances as appropriate). Statistical significance was set at *P* < 0.05.

Intensity-time curves were generated using RStudio (version 2024.09.1, PBC, Boston, MA, USA) and GraphPad Prism (version 10, GraphPad Software, Boston, MA, USA) for features with significant *P* values at both 2 (urine only), 4, and 6 hours to exclude features not regulated by GHB intake for at least 6 hours. On the basis of visual inspection, features exhibiting a consistent increase or decrease in the GHB group relative to placebo across all relevant time points were selected for annotation. Features showing substantial baseline differences or inconsistent changes between groups were excluded. In addition, a manual search for GHB and known GHB metabolites (e.g., GHB-glutamate, GHB-carnitine, GHB-glycine, GHB-pentose, GHB-taurine, 2,4-DHB, 3,4-DHB, glycolic acid, succinic acid, and succinyl carnitine) was conducted.

### Metabolite identification

Selected features were attempted identified by exporting extracted ion chromatograms and compound spectra from Bruker Compass DataAnalysis (version 4.1, Bruker Daltonics, Bremen, Germany) and importing to SIRIUS (version 6, University of Jena, Germany), MetFrag (https://msbi.ipb-halle.de/MetFrag/), and the Human Metabolome Database (http://hmdb.ca).

Metabolite identification followed the Metabolomics Standards Initiative (*20*): Level 1, confirmed by matching *m/z*, retention time, and MS/MS to an authentic standard; however, structural isomers were not systematically investigated unless considered relevant. Level 2, putatively annotated via mass and fragment matching to databases. Level 3, putatively characterized at the class level. Level 4, unknown. When comparing features to reference standards or previously reported metabolites, an *m/z* deviation of no more than ±0.005 Da was accepted.

### Chemical synthesis of α- and β-anomer of GHB-ribose

The two anomers of GHB-ribose were synthesized in five steps starting from benzylated ribose and gamma-butyrolactone (GBL) (Fig. 4B). In brief, benzylated ribose was initially converted into the corresponding ribofurasonyl donor by trichloroacetimidate activation of the anomeric ribofurasonylic hydroxy group. This donor was in a second step coupled to the benzyl ester of gamma-butyric acid leading to a 3:1 mixture of the two epimeric and protected target compounds. Following chromatographic purification, the individual compounds were isolated in 72 and 24% yield based on the benzylated ribose. Last, the epimers were deprotected by palladium catalyzed hydrogenolysis to give the α- and β-anomers in 94 and 99% yield, respectively. The full experimental details and characterization is described in the Supplementary Materials.

### Quantitative analysis of GHB-β-ribose in blood and urine samples

Quantification of GHB-β-ribose in whole blood and urine was performed using the GHB method published by Sørensen *et al.* (*6*) only modified by addition of ion transitions and calibration functions for GHB-β-ribose analysis. Briefly, whole blood and urine samples were extracted in MeOH/ACN and processed by anion exchange SPE using a NH_2_ sorbent. After evaporation of the solvent, the residue was reconstituted and analyzed by LC-MS/MS using a reversed-phase LC column with anion properties. The instrument consisted of an Exion UHPLC system (Sciex, Ontario, Canada) and a Sciex QTRAP 6500+ mass spectrometer with a TurboIonSpray probe for ESI. The conditions used for multiple reaction monitoring are listed in table S2.

The quantification was performed using GHB-^13^C_4_ as IS due to the lack of an isotopic analog of GHB-β-ribose. The GHB-^13^C_4_ response was adjusted for concentration dependent ion suppression effects caused by GHB present in blood and urine matrices. Calibration was performed using pure solvent calibrants in 10% MeOH at concentrations corresponding to GHB-β-ribose (0.001, 0.005, 0.01, 0.1, 1, 10, 20, 30, and 40 μg/ml) for whole blood and urine. Measured GHB-β-ribose concentrations were lastly adjusted for relative mean differences in area ratio (GHB-β-ribose/IS) between calibrants and matrix samples to match concentration measurements in pure solvent, blood, and urine to each other.

The RSD_r_ also termed intraday precision, and the RSD_R,intralab_ also termed interday precision were determined using different whole blood and urine samples that were analyzed with and without spiking at different concentration levels. Whole blood samples were spiked with GHB-β-ribose (0.005 and 0.1 μg/ml), whereas urine samples were spiked with GHB-β-ribose (0.1, 1 and 10 μg/ml). Duplicate analyses were performed on 6 different days. The method trueness was determined from the results obtained for the spiked samples in the precision study. The trueness was expressed as the relative bias; relative bias = (mean test result of spiked sample − mean test result of unspiked sample − spiked concentration) × 100/spiked concentration. The acceptance criteria were a maximum RSD_R,intralab_ of 15% and a bias within ±15%.

The LLOQ was determined from a similar precision study using blank samples spiked with GHB-β-ribose to an S/N level of ∼10 for the quantifier ion. The acceptance criteria for the LLOQ were a maximum RSD_R,intralab_ of 20% and a bias within ±20%. The ULOQ was defined by the highest calibrant. The LOD was determined using blank baseline samples from the trial participants in the placebo group (*n* = 15) spiked at different trace concentrations resulting in S/N ratios of ∼3 to 10 for the individual product ions. The method LOD was defined as the lowest spiked concentration resulting in at least two significant product ions with a peak area ratio within ±40% of the calibrants.

Matrix effects (ion suppression and ion enhancement effects) were determined using final sample extracts from different whole blood and urine samples (*n* = 15 of each) that were spiked with GHB-β-ribose at concentrations equivalent to 0.005 and 0.1 μg/ml in blood and 0.1, 1, and 10 μg/ml in urine, respectively. The samples were analyzed along with pure standards at the same concentration and unspiked samples. The matrix effect for each sample was calculated from the peak areas (*A*) without IS correction: matrix effect (%) = [*A*_pure standard_ − (*A*_spiked sample_ − *A*_unspiked sample_)] × 100/*A*_pure standard_. Extraction recoveries were determined without IS correction from the same samples spiked before extraction at 0.005 and 0.1 μg/ml in blood and 0.1, 1, and 10 μg/ml in urine, respectively. Recovery was calculated as the percentage analyte response of a sample spiked before processing compared with the response of the same unspiked sample that was spiked after processing at a concentration corresponding to 100% recovery: recovery (%) = [(*A*_spiked sample_ − *A*_unspiked sample_)/(*A*_sample spiked after processing at 100% recovery_ − *A*_unspiked sample_)] × 100.

Detection of GHB-β-ribose required presence of at least one qualifier product ion with a maximum difference in the ion ratio (qualifier/quantifier) of ±40% between the sample and calibrants and a concentration above LOD. Furthermore, S/N ratios of at least 3 was required for the quantifier and primary qualifier ion.

The cutoff value to distinguish between GHB-β-ribose concentrations originating from GHB intake and endogenous concentrations was calculated in the 100 authentic samples by one-tailed statistics using a confidence level of 99.9% to ensure a false-positive rate of no more than 1 in 1000 samples. The calculation was performed on ln-transformed data to ensure a normal distribution of data points, which was tested by the Shapiro-Wilk test using RStudio.

Concentrations of GHB-β-ribose were displayed as median and range in μg/ml using GraphPad Prism 10.6.1 (GraphPad Software, Boston, USA). The concentrations in urine samples were not normalized to creatinine content.

All samples from the GHB and placebo group were originally quantified for GHB blinded for the analytical chemist (*18*). In this study, the same samples were reanalyzed with the method described above. To avoid false-positive results, the samples were separated into different analytical batches according to the treatment group to minimize the risk of carryover. The 100 authentic samples were analyzed in separate batches. GHB concentrations from the two separate determinations were compared as well as random urine samples were reanalyzed to verify the varying trends of GHB-β-ribose time profiles (10% of the GHB group and 5% of the placebo group). No differences were observed.

### Ethics

The clinical trial was approved by the Danish Medicines Agency and the Danish Medical Research Ethics Committees (EU CT 2022-501329-18-00) and performed in accordance with the Declaration of Helsinki. All trial participants provided written informed consent. Authentic samples were anonymized. Data were handled according to the General Data Protection Regulation and the Danish Data Protection Act.

## Supplementary Materials

### This PDF file includes:

Supplementary Text Figs. S1 to S31 Tables S1 and S2 References
