# Supplementary material for: Identification of gamma-hydroxybutyrate (GHB)–β-ribose as a promising biomarker enabling prolonged detection of GHB intake
Source: Sci Adv. 2026 Nov 13;12(46):eaeh1261. doi: 10.1126/sciadv.aeh1261 (PMC13763946; doi:10.1126/sciadv.aeh1261)
Supplement: Supplementary file 1 [file sciadv.aeh1261_sm.pdf]

Science Advances
AAAS

Supplementary Materials for

Identification of gamma-hydroxybutyrate (GHB)–β-ribose as a promising biomarker enabling prolonged detection of GHB intake

Kathrine Bohn Faldborg *et al.*

Corresponding author: Charlotte Uggerhøj Andersen, cua@forens.au.dk;
Kirstine Lykke Nielsen, klyn@forens.au.dk

Sci. Adv. **12**, eaeh1261 (2026)
DOI: 10.1126/sciadv.aeh1261

This PDF file includes:

Supplementary Text
Figs. S1 to S31
Tables S1 and S2
References

Chemical synthesis of α- and β-anomer of GHB-ribose

All reactions were conducted in flame-dried glassware under an atmosphere of argon unless otherwise stated. Reagents were used as received from commercial suppliers unless otherwise stated (Merck), 2,3,5-Tri-*O*-benzyl-D-ribofuranose was purchased from AstaTech. CH_2Cl_2, was dried over aluminium oxide via a MBraun SPS-800 solvent purification system and further dried over preactivated molecular sieves (4 Å). Molecular sieves were activated by drying in the oven at 120 °C for at least 24 hours, before they were heated in a microwave at maximum power for 1.5 minutes, followed by evaporation of the formed vapour on the high vacuum line. This was repeated for multiple cycles, until vapour formation seized (ca. 4 to 6). Concentration in vacuo was performed using a rotary evaporator with a water bath temperature of 35 °C, followed by further concentration using a high vacuum pump. Lyophilization was carried out on a Christ freeze drier beta 2-8 LSCplus. TLC analysis was carried out on silica gel coated aluminum foil plates (Merck; silica gel 60 matrix; 20 cm × 20 cm; organic polymer binder; fluorescent indicator F_{254}; 200 µm layer thickness and 10-12 µm particle size). The TLC plates were visualized by UV irradiation and/or by staining with either CAM stain ($(NH_4)_6Mo_7O_{24}\cdot 4H_2O$ (10 g), ammonium cerium(IV) sulfate (4 g), 10% H_2SO_4 (aq., 400 mL)), or $KMnO_4$ stain ($KMnO_4$ (5.0 g), 5 % NaOH (aq., 8.3 mL) and K_2CO_3 (33.3 g) in H_2O (500 mL)). Automated flash column chromatography (AFCC) was carried out with Interchim puriFlash® 5.050 using 30 µm prepacked columns, the method may be specified using column volumes (cv). Mass spectra (HRMS) were recorded on a Bruker Daltonics MicrOTOF time-of-flight spectrometer with positive electrospray ionization, or negative ionization when stated. Nuclear magnetic resonance (NMR) spectra were recorded, a Bruker BioSpin GmbH 400 MHz spectrometer, 400 and 101 MHz for ^{1}H and ^{13}C, respectively. ^{13}C NMR was acquired in broad band decoupled mode, {1H}. Chemical shifts (δ) are reported in ppm relative to the (residual) solvent signal ($CDCl_3$: 7.26 ppm ^{1}H NMR, 77.16 ppm ^{13}C NMR, D_2O: 4.79 ppm ^{1}H NMR). Multiplicities are indicated using the following abbreviations: s = singlet, d = doublet, t = triplet, q = quartet, p = pentet , m = multiplet, br = broad. Full characterizations were conducted by employing 2D NMR experiments (COSY, NOESY, multiplicity-edited HSQC, and HMBC, etc.) as appropriate.

Synthesis:

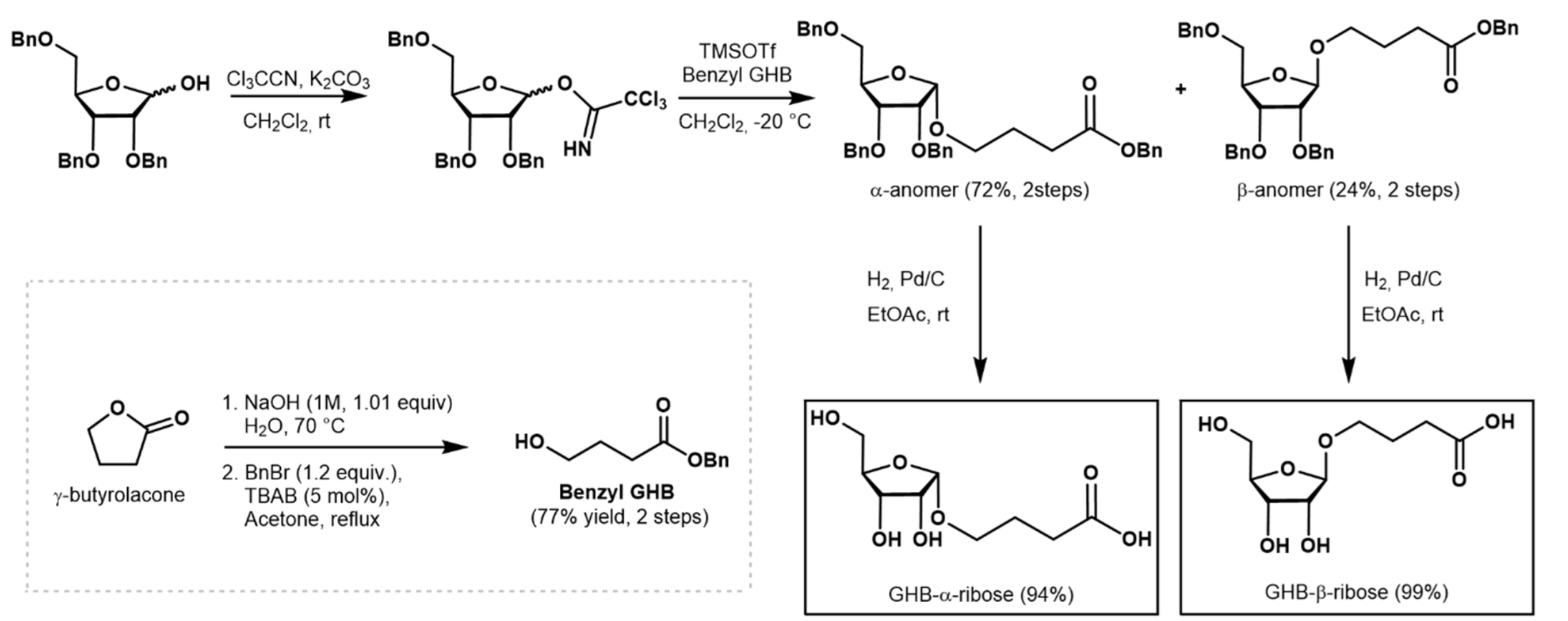

Benzyl 4-hydroxybutanoate

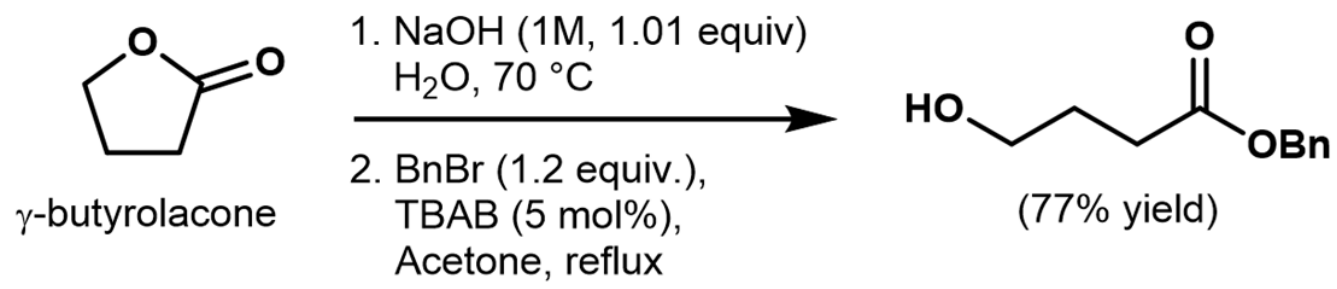

According to literature procedure:[29] γ-butyrolactone (510 mg, 5.93 mmol, 1.00 equiv.) was dissolved in 6.0 mL 1M NaOH (6.0 mmol, 1.01 equiv.), and the corresponding mixture was heated to 70 ºC and stirred for 24 h. After full consumption of starting material was observed by TLC (30% EtOAc in Heptane) the mixture was cooled to room temperature for 15 mL of toluene was added. The resulting biphasic mixture was concentrated under reduced pressure, and the corresponding residue was dissolved in acetone (6.0 mL). To this mixture was added tetrabutylammoniumbromide (96 mg, 0.296 mmol, 0.05 equiv.) and BnBr (0.851 mL, 7.11 mmol, 1.2 equiv.) and the resulting suspension was heated until acetone refluxed. After 24 h the mixture was concentrated *in vacuo*, dissolved in EtOAc, washed with 1M $NaHSO_4$, aq. sat. $NaHCO_3$, and brine, dried over Na_2SO_4 and finally concentrated. The crude residue was purified by AFCC (40g column, 20 CV, 0 to 30% EtOAc in heptane, eluting at 23% (15 CV)), to yield benzyl 4-hydroxybutanoate as a colorless oil (892 mg, 4.59 mmol, 77%).

^{1}H-NMR	(400 MHz, $CDCl_3$)
	δ 7.43 – 7.28 (m, 5H), 5.13 (s, 2H), 3.68 (td, J = 6.4, 2.7 Hz, 2H), 2.49 (t, J = 7.2 Hz, 2H), 1.90 (tt, J = 7.2, 6.1 Hz, 2H).
^{13}C-NMR	(101 MHz, $CDCl_3$)
	δ 173.9, 136.0, 128.7, 128.4, 128.3, 66.5, 62.1, 31.1, 27.8.
R_f	0.36 (1:1 EtOAc/Heptane; UV/$KMnO_4$).
HRMS(ESI)	Calc'd *m/z* for $C_{11}H_{14}NaO_3^+$ 217.0836 $[M+Na]^+$; found 217.0833.

For NMR spectra see Fig. S9 and S10.

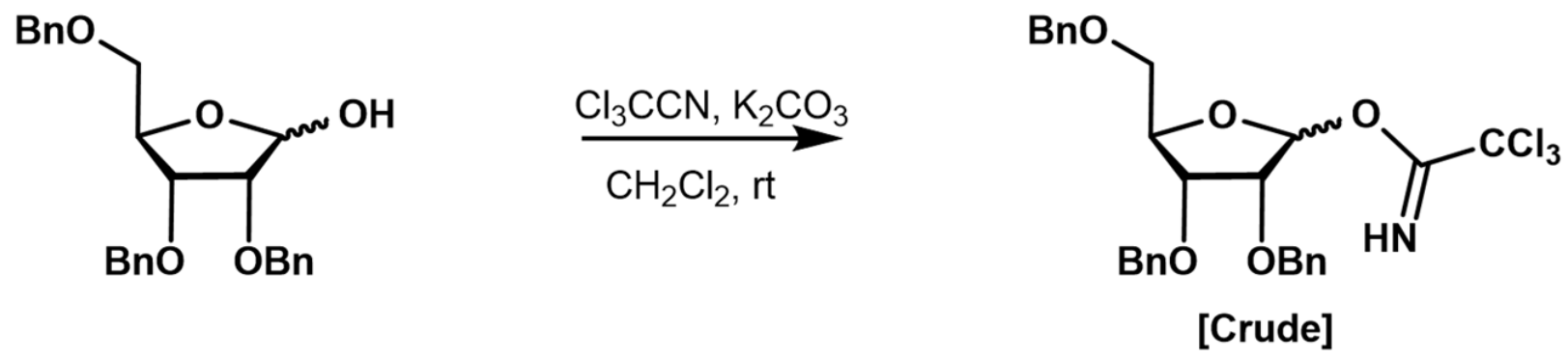

According to literature procedure:[30] 2,3,5-Tri-*O*-benzyl-D-ribofuranose (172 mg, 0.409 mmol) was dissolved in 1.2 mL anhydrous CH_2Cl_2. To this solution trichloroacetonitrile (0.41 mL, 4.1 mmol, 10 equiv.) and K_2CO_3 (68mg, 0.49 mmol, 1.2 equiv.) were added. The resulting suspension was allowed to stir at room temperature overnight after which the suspension was filtered over celite, and the filtrate was concentrated under reduced pressure to afford a yellow-orange oil. The resulting trichloroacetimidate was used in the subsequent step without further purification.

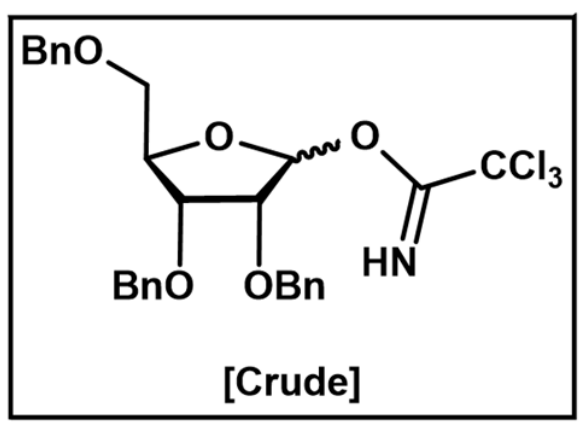

^{1}H NMR (400 MHz, $CDCl_3$)

δ 8.47 (s, 1H), 7.41 – 7.26 (m, 15H), 6.32 (s, 1H), 4.80 (d, *J* = 12.1 Hz, 1H), 4.67 (d, *J* = 12.1 Hz, 1H), 4.63 – 4.39 (m, 5H), 4.19 – 4.08 (m, 1H), 4.07 (d, *J* = 4.6 Hz, 1H), 3.71 (dd, *J* = 11.0, 3.6 Hz, 1H), 3.62 (dd, *J* = 11.0, 5.2 Hz, 1H).

R_f 0.58 (1:1 EtOAc/Heptane; UV, $KMnO_4$).

For NMR spectra see Fig. S11.

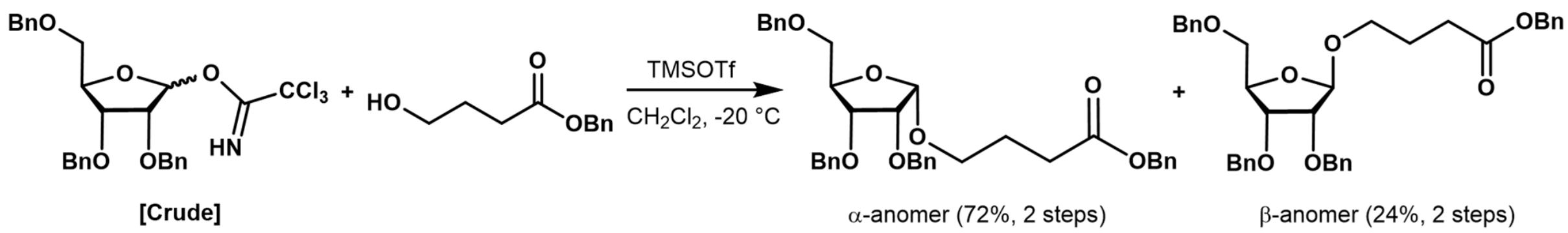

Adapted from literature procedure:[30] Trichloroacetimidate from the previous step (assuming quantitative yield from the previous step) was dissolved in 0.6 mL of anhydrous CH_2Cl_2 under an argon atmosphere. To the solution was added 25 mg of freshly activated molecular sieves followed by addition of a solution of benzyl 4-hydroxybutanoate in 0.4 mL anhydrous CH_2Cl_2. The resulting suspension was cooled to – 20 ºC and after stirring at this temperature for 5 minutes, trimethylsilyl triflate (11 µL, 61 µmol, 0.15 equiv.) was slowly added. After 1 hour, TLC (1:1 EtOAc/Heptane) indicated full conversion of trichloroacetimidate and the reaction was quenched using triethylamine. All volatiles were removed *in vacuo* and the crude material was purified by AFCC (40g column, 0-30% EtOAc over 17 CV, β-anomer elutes at 21.5-24% EtOAc, α-anomer elutes at 25.5-29% EtOAc) to afford the pure anomers as colorless liquids (α-anomeric product 177 mg, 0.297 mmol, 72%, β-anomeric product 59 mg, 0.0989 mmol, 24%). The anomeric configuration was assigned based on ^{1}H NMR coupling constants of the anomeric protons (α: d, J = 4.1 Hz, β: 1.1 Hz)[31,32] and ^{13}C NMR, gHMBC, gHSQC and NOESY of the debenzylated coupling products, all of which are consistent with literature.[33]

benzyl 4-(((2*S*,3*S*,4*S*,5*R*)-3,4-bis(benzyloxy)-5-((benzyloxy)methyl)tetrahydrofuran-2-yl)oxy)butanoate

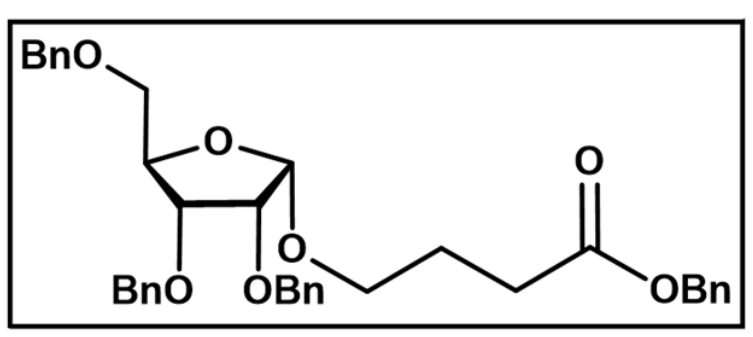

^{1}H-NMR (400 MHz, $CDCl_3$)

δ 7.37 – 7.24 (m, 18H), 7.23 – 7.18 (m, 2H), 5.11 (s, 2H), 4.96 (d, J = 4.1 Hz, 1H), 4.67 (d, J = 12.6 Hz, 1H), 4.62 (s, 2H), 4.53 (d, J = 12.6 Hz, 1H), 4.48 (d, J = 12.1 Hz, 1H), 4.41 (d, J = 12.1 Hz, 1H), 4.20 (q, J = 3.8 Hz, 1H), 3.81 (dd, J = 6.7, 3.3 Hz, 1H), 3.77 (td, J = 6.8, 4.4 Hz, 2H), 3.57 (dt, J = 10.0, 6.1 Hz, 1H), 3.40 (dd, J = 10.5, 3.9 Hz, 1H), 3.32 (dd, J = 10.5, 4.1 Hz, 1H), 2.53 (td, J = 7.5, 4.4 Hz, 2H), 2.01 (p, J = 7.0 Hz, 2H).

^{13}C-NMR (101 MHz, $CDCl_3$)

δ 173.5, 138.4, 138.1, 138.0, 136.2, 128.7, 128.5, 128.5, 128.4, 128.3, 128.3, 128.3, 128.1, 127.9, 127.8, 127.8, 127.7, 101.4, 81.9, 77.7, 75.4, 73.5, 72.6, 72.4, 70.1, 67.0, 66.3, 31.1, 25.2.

R_f 0.60 (1:1 EtOAc/Heptane; UV/$KMnO_4$).

HRMS(ESI) Calc'd m/z for $C_{37}H_{40}NaO_7^+$ 619.2667 [M+Na]$^+$; found 619.2670.

For NMR spectra see Fig. S12-S15.

benzyl 4-(((2*R*,3*R*,4*R*,5*R*)-3,4-bis(benzyloxy)-5-((benzyloxy)methyl)tetrahydrofuran-2-yl)oxy)butanoate

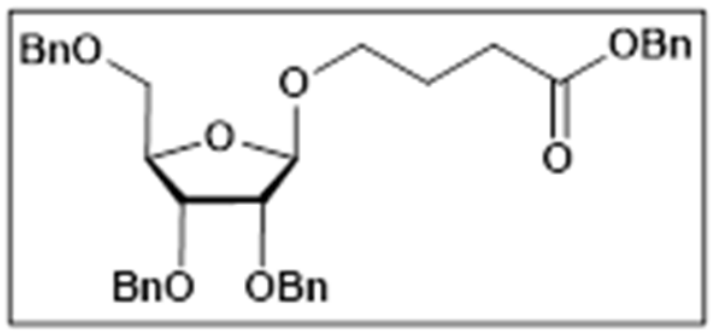

^{1}H-NMR	(400 MHz, $CDCl_3$)
	δ 7.39 – 7.27 (m, 20H), 5.09 (s, 2H), 4.97 (d, *J* = 1.1 Hz, 1H), 4.65 (d, *J* = 12.1 Hz, 1H), 4.60 (d, *J* = 12.1 Hz, 1H), 4.55 (dd, *J* = 12.1, 5.5 Hz, 2H), 4.51 (d, *J* = 12.3 Hz, 1H), 4.44 (d, *J* = 11.8 Hz, 1H), 4.32 (ddd, *J* = 7.1, 5.8, 3.7 Hz, 1H), 4.00 (dd, *J* = 7.1, 4.7 Hz, 1H), 3.82 (dd, *J* = 4.7, 1.1 Hz, 1H), 3.69 (dt, *J* = 9.7, 6.2 Hz, 1H), 3.59 (dd, *J* = 10.5, 3.7 Hz, 1H), 3.48 (dd, *J* = 10.6, 5.8 Hz, 1H), 3.36 (dt, *J* = 9.7, 6.1 Hz, 1H), 2.41 – 2.25 (m, 2H), 1.88 – 1.76 (m, 2H).
^{13}C-NMR	(101 MHz, $CDCl_3$)
	δ 173.3, 138.4, 138.0, 136.1, 128.7, 128.5, 128.5, 128.4, 128.4, 128.3, 128.1, 128.0, 127.9, 127.9, 127.8, 127.7, 105.3, 80.5, 79.8, 78.6, 73.2, 72.5, 72.4, 71.4, 66.7, 66.3, 31.2, 25.0.
R_f	0.66 (1:1 EtOAc/Heptane; UV/$KMnO_4$).
HRMS(ESI)	Calc'd *m/z* for $C_{37}H_{40}NaO_7^+$ 619.2667 $[M+Na]^+$; found 619.2671.

For NMR spectra see Fig. S16-S19.

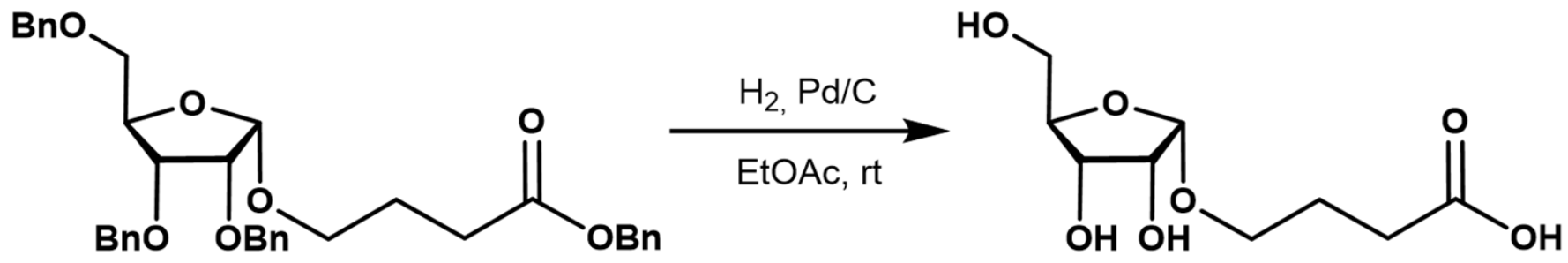

(94%)

α-Glycosylation product from the previous step (20 mg, 0.034 mmol, 1.0 equiv.) was dissolved in 2 mL of EtOAc, followed by addition of 5 mg of Palladium on carbon (10% wt. loading, Sigma) and the reaction was stirred under hydrogen atmosphere (using hydrogen balloon) for 9 hours. The suspension diluted with water and filtered through a PTFE hydrophilic syringe filter (22 μm membrane) and the solvent was removed under reduced pressure to afford the product as a sticky color oil (7.4 mg, 0.031 mmol, 94%).

4-(((2*S*,3*R*,4*S*,5*R*)-3,4-dihydroxy-5-(hydroxymethyl)tetrahydrofuran-2-yl)oxy)butanoic acid

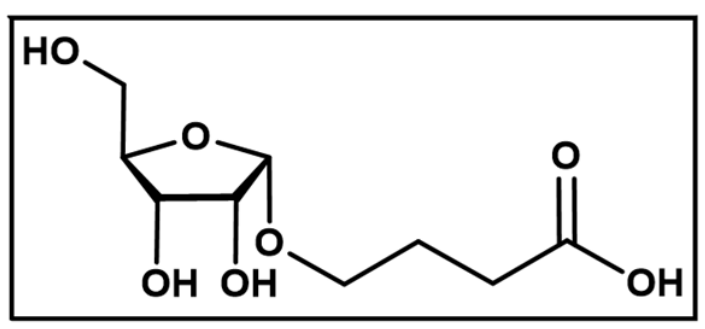

^{1}H-NMR (400 MHz, D_2O)

δ 5.07 (d, *J* = 4.3 Hz, 1H), 4.11 – 4.04 (m, 2H), 4.00 (dd, *J* = 6.3, 3.7 Hz, 1H), 3.76 (dt, *J* = 10.1, 6.4 Hz, 1H), 3.71 (dd, *J* = 12.6, 3.4 Hz, 1H), 3.63 (dd, *J* = 12.4, 4.5 Hz, 1H), 3.56 (dt, *J* = 10.1, 5.8 Hz, 1H), 2.45 (t, *J* = 7.3 Hz, 2H), 1.92 – 1.84 (m, 2H).

^{13}C-NMR (101 MHz, D_2O)

δ 178.5, 102.0, 84.2, 71.0, 69.5, 67.6, 61.3, 30.5, 24.3.

HRMS Calc'd *m/z* for $C_9H_{15}O_7^-$ 235.0823 $[M-H]^-$; found 235.0825

For NMR spectra see Fig. S20-S25.

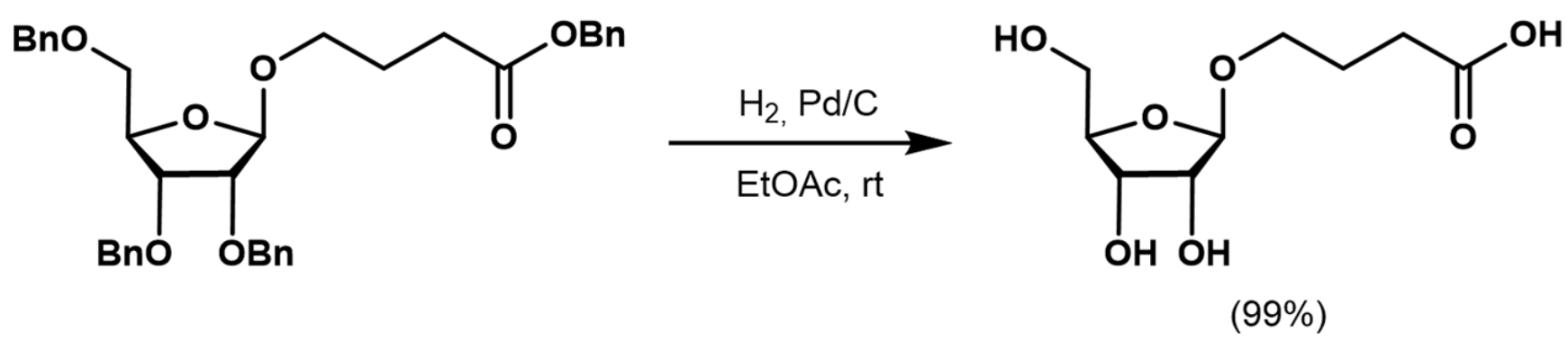

β-Glycosylation product from the coupling (20 mg, 0.034 mmol, 1.0 equiv.) was dissolved in 2 mL of EtOAc, followed by addition of 5 mg of Palladium on carbon (10% wt. loading, Sigma) and the reaction was stirred under hydrogen atmosphere (using hydrogen balloon) for 9 hours. The suspension diluted with water and filtered through a PTFE hydrophilic syringe filter (22 µm membrane) and the solvent was removed under reduced pressure to afford the product as a sticky color oil (7.8 mg, 0.033 mmol, 98%).

4-(((2*R*,3*R*,4*S*,5*R*)-3,4-dihydroxy-5-(hydroxymethyl)tetrahydrofuran-2-yl)oxy)butanoic acid

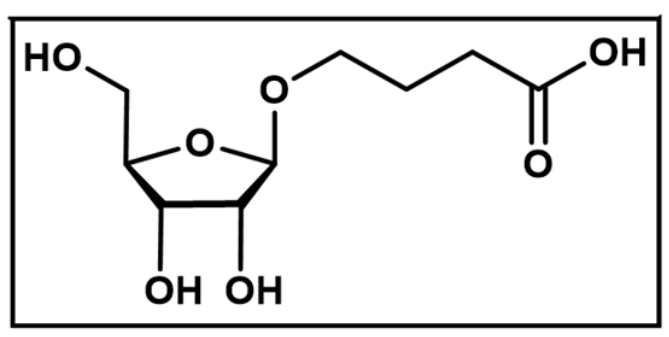

<u>^{1}H-NMR</u> (400 MHz, D_2O)

δ 4.94 (d, *J* = 1.1 Hz, 1H), 4.12 (dd, *J* = 7.1, 4.6 Hz, 1H), 4.00 – 3.91 (m, 1H), 3.78 – 3.74 (m, 1H), 3.74 – 3.69 (m, 1H), 3.57 (dd, *J* = 12.3, 6.7 Hz, 1H), 3.50 (dt, *J* = 9.9, 6.1 Hz, 1H), 2.40 (t, *J* = 7.2 Hz, 2H), 1.93 – 1.75 (m, 2H).

<u>^{13}C-NMR</u> (101 MHz, D_2O)

δ 178.4, 106.6, 82.5, 74.2, 70.8, 67.1, 62.9, 30.7, 24.1.

<u>HRMS</u> Calc'd *m/z* for $C_9H_{15}O_7^-$ 235.0823 [M-H]$^-$; found 235.0823

For NMR spectra see Fig. S26-S31.

LC-TIMS-TOF-MS analysis

Chromatographic separation was performed using the same ACQUITY I-Class UPLC system (Waters Corporation, Milford, MA, USA) equipped with an ACQUITY HSS T3 C18 column (100 mm × 2.1 mm, 1.8 µm, Waters) as in the untargeted metabolomics analysis. The chromatographic conditions were additionally similar.

Mass spectrometric detection was carried out on a Bruker timsMetabo (Bruker Daltonics, Bremen, Germany) operated in negative VIP-HESI (vacuum insulated probe heated ESI) mode with tims enabled. The acquisition was performed in PASEF scan mode with 2 PASEF ramps, a ramp time of 100 ms, and an accumulation time of 30 ms. The scan range was *m/z* 20-1400 and the ion mobility range was set to $1/k_0$ 0.30-1.5 V·s /cm^2. Collision energies were 10 eV (CE1) and 20 eV (CE2). The capillary voltage in the VIP-HESI source was set to 4500 V, the nebulizer pressure was 2 bar, with a drying gas flow of 10 L/min at 220°C, while the sheath gas (N_2) flow was 4 L/min at 400°C. For mass accuracy and ion mobility calibration, the instrument was calibrated using a combination of ESI Tune Mix and sodium formate, providing well-defined cluster ions across the *m/z* and $1/k_0$ ranges to ensure accurate alignment of both mass and mobility axes.

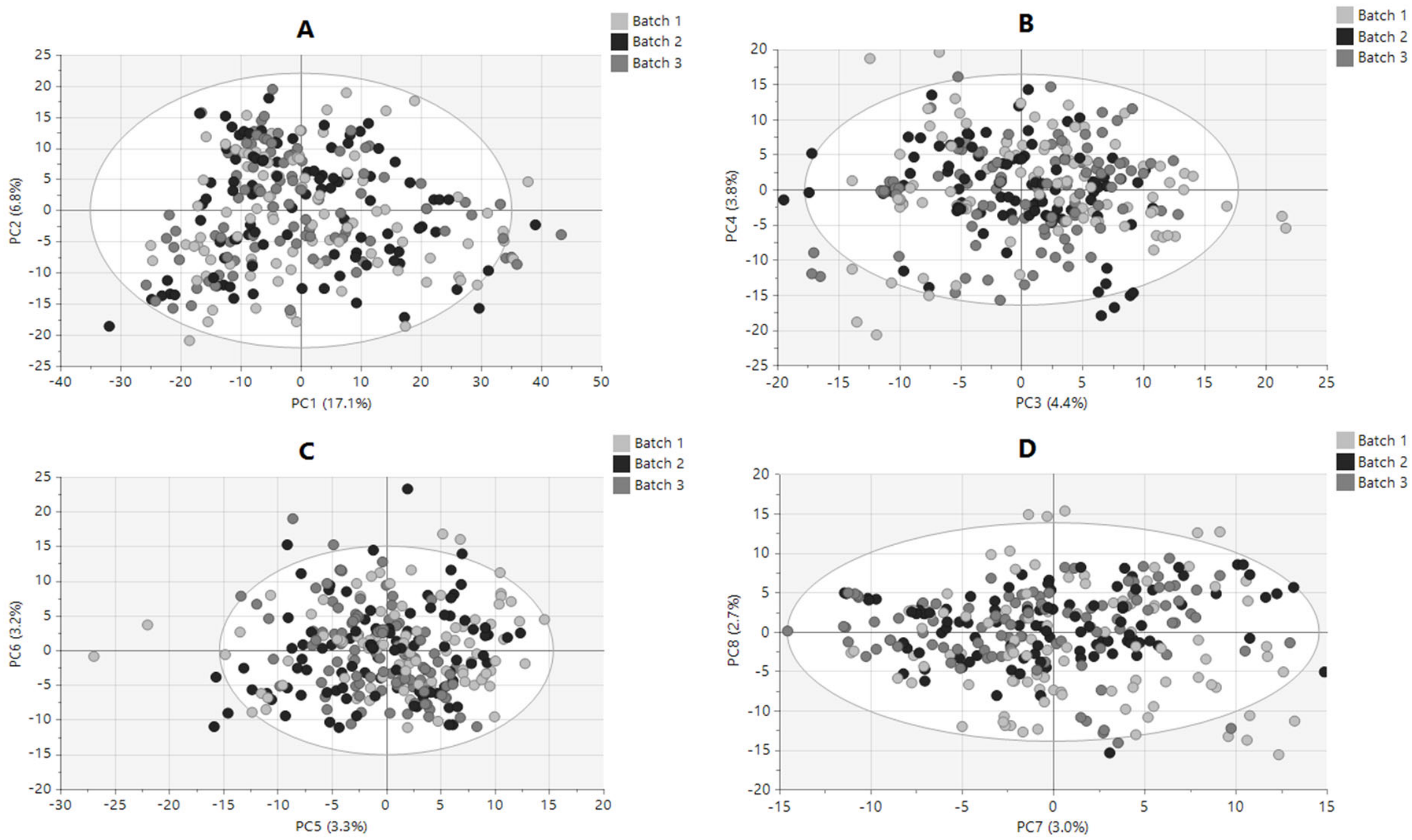

Fig. S1. PCA scores plots of urine samples acquired in positive electrospray ionisation (ESI) mode (normalized data). Samples are coloured by analytical batch to visualise potential batch effects. **A**–**D**) show different combinations of principal components: **A**) PC1 vs. PC2, **B**) PC3 vs. PC4, **C**) PC5 vs. PC6, and **D**) PC7 vs. PC8. The percentage of total variance explained by each component is indicated in parentheses on the axis labels.

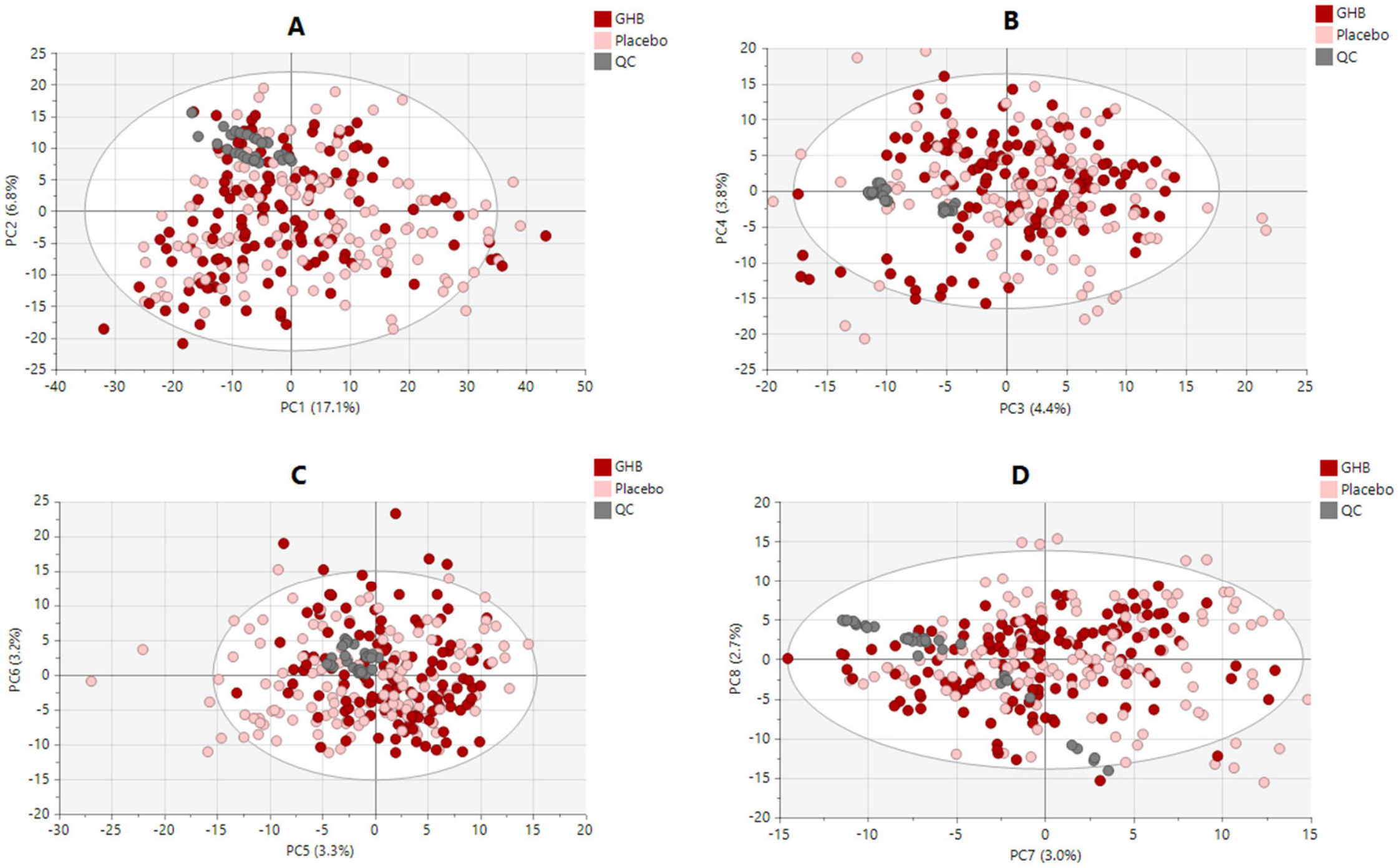

Fig. S2. PCA scores plots of all urine samples acquired in positive ESI mode (normalized data). Samples are coloured by treatment group or quality control (QC). Baseline and late post-administration samples are included in the GHB group, which may obscure potential differences between treatment conditions. **A**–**D**) show different combinations of principal components: **A**) PC1 vs. PC2, **B**) PC3 vs. PC4, **C**) PC5 vs. PC6, and **D**) PC7 vs. PC8. The percentage of total variance explained by each component is indicated in parentheses on the axis labels.

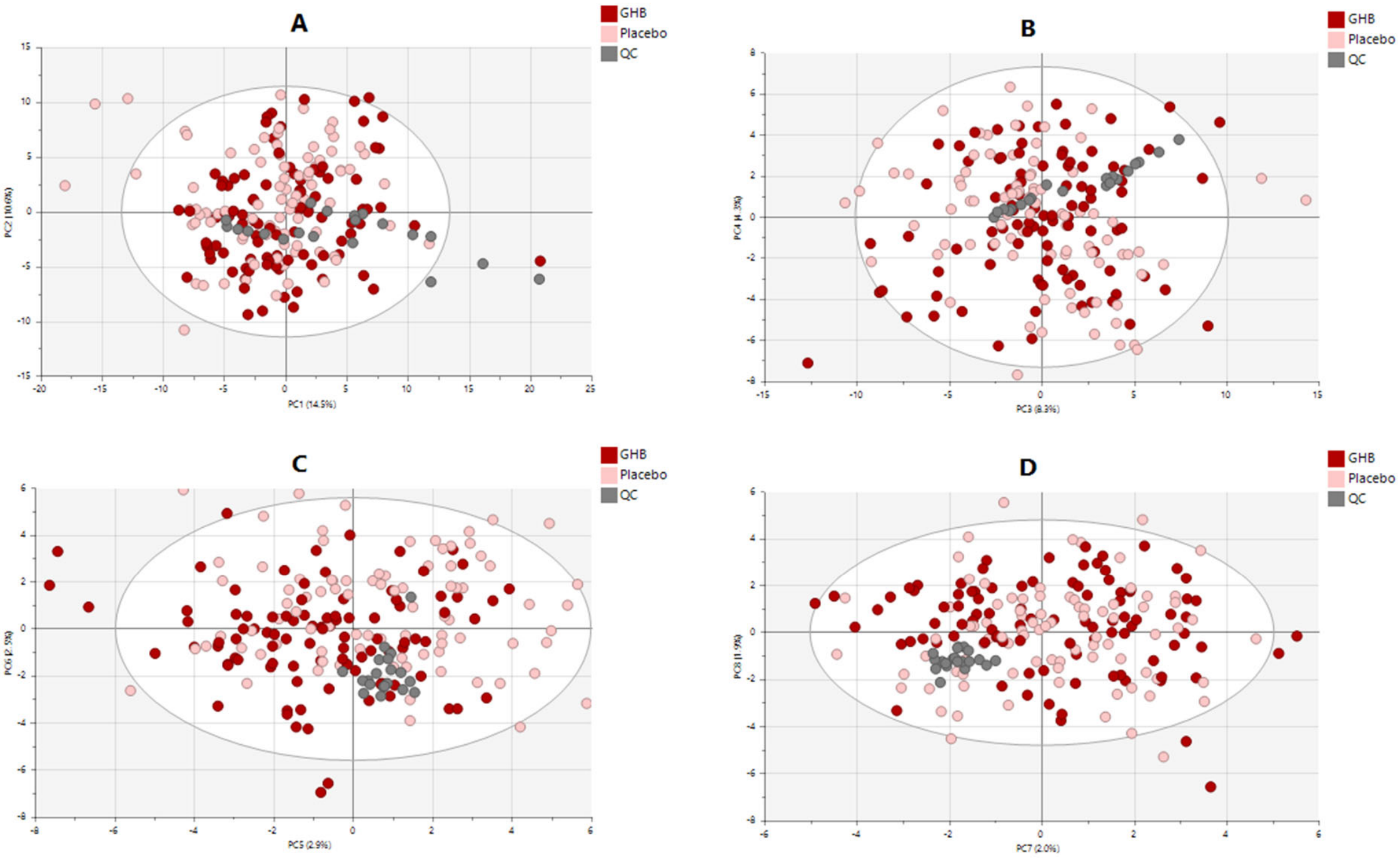

Fig. S3. PCA scores plots of all blood samples acquired in positive ESI mode (normalized data). Samples are coloured by treatment group or quality control (QC). Baseline and late post-administration samples are included in the GHB group, which may obscure potential differences between treatment conditions. **A**–**D**) show different combinations of principal components: **A**) PC1 vs. PC2, **B**) PC3 vs. PC4, **C**) PC5 vs. PC6, and **D**) PC7 vs. PC8. The percentage of total variance explained by each component is indicated in parentheses on the axis labels.

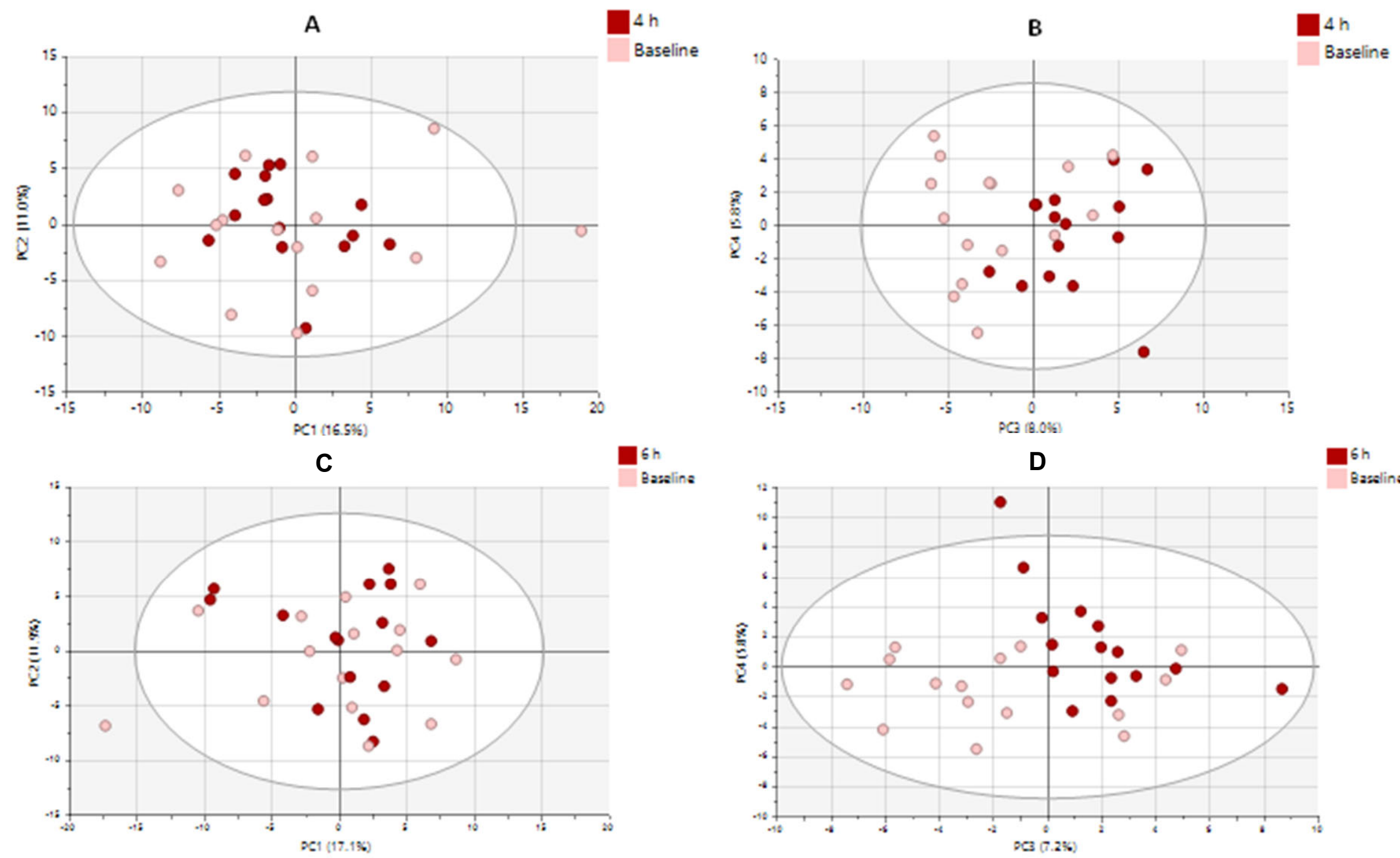

Fig. S4. PCA scores plots of blood samples from the GHB treatment group, acquired in positive electrospray ionisation (ESI) mode (normalized data). A) PCA of baseline and 4-hour samples showing PC1 vs. PC2 and **B**) PC3 vs. PC4. **C**) PCA of baseline and 6-hour samples showing PC1 vs. PC2 and **D**) PC3 vs. PC4. Samples are coloured by time point: baseline (light red) and post-administration (dark red). The percentage of total variance explained by each component is indicated in parentheses on the axis labels.

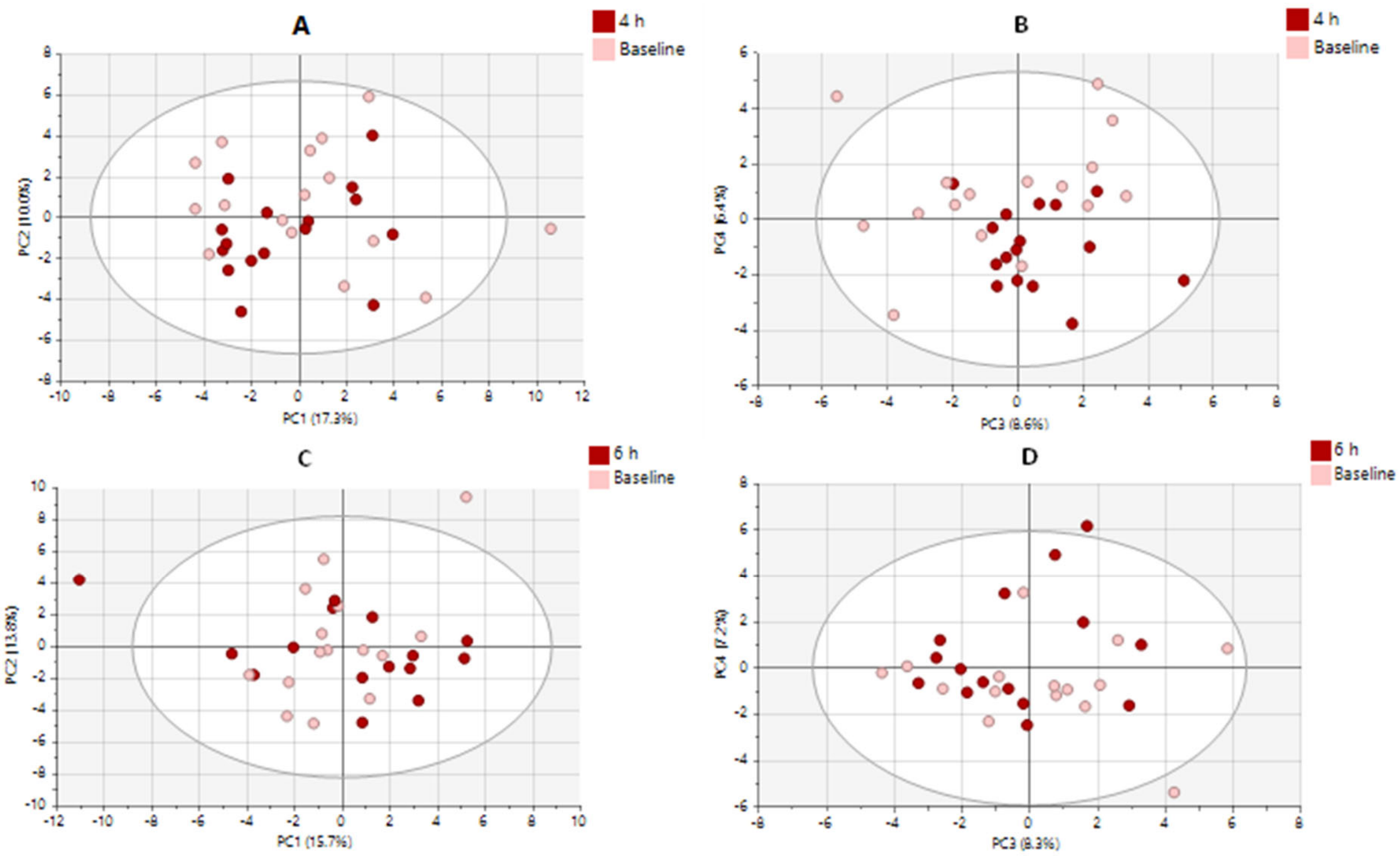

Fig. S5. PCA scores plots of blood samples from the GHB treatment group, acquired in negative electrospray ionisation (ESI) mode (normalized data). A) PCA of baseline and 4-hour samples showing PC1 vs. PC2 and **B**) PC3 vs. PC4. **C**) PCA of baseline and 6-hour samples showing PC1 vs. PC2 and **D**) PC3 vs. PC4. Samples are coloured by time point: baseline (light red) and post-administration (dark red). The percentage of total variance explained by each component is indicated in parentheses on the axis labels.

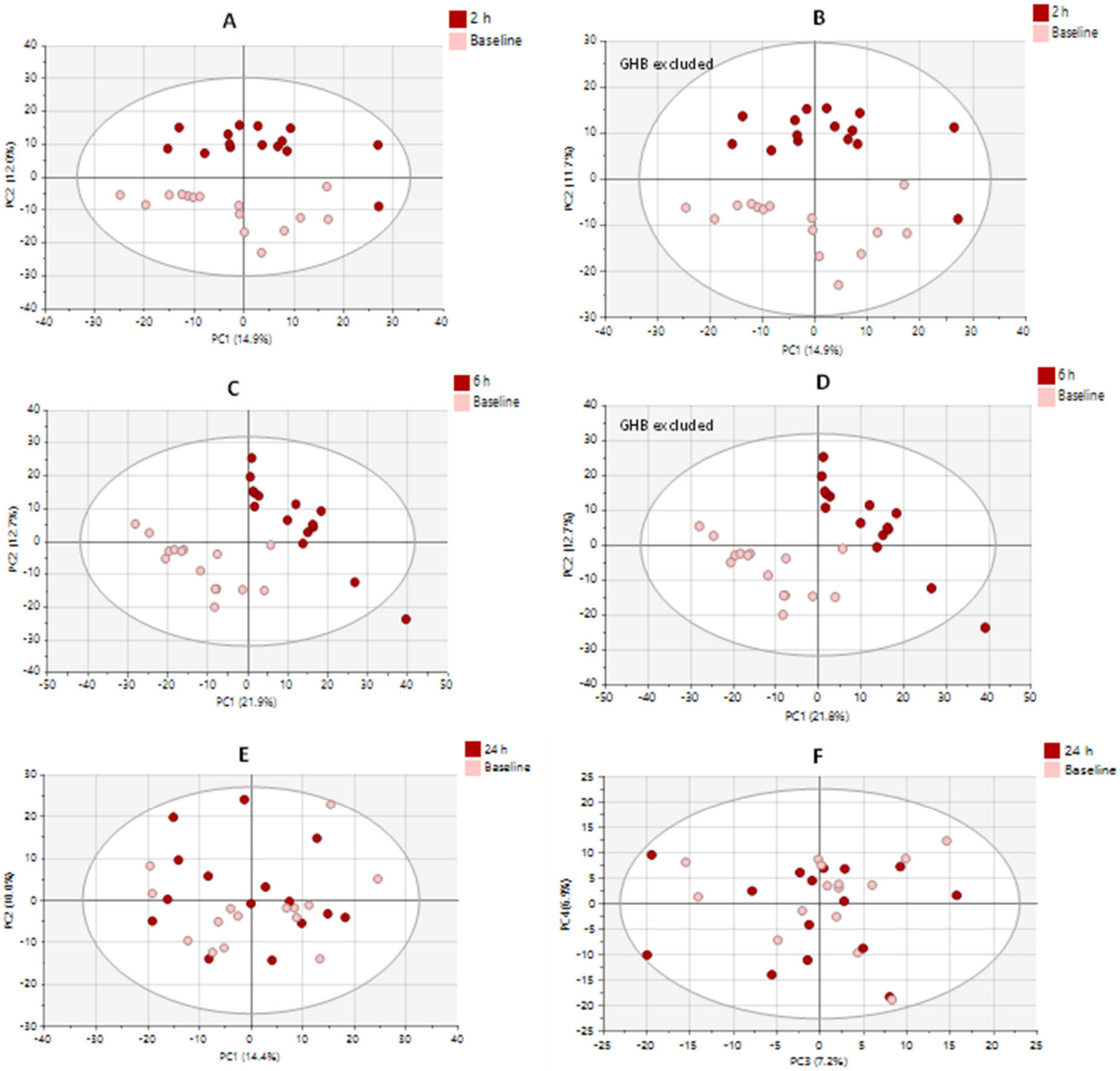

Fig. S6. PCA scores plots of urine samples from the GHB treatment group, acquired in positive electrospray ionisation (ESI) mode (normalized data). A) PCA of baseline and 2-hour samples showing PC1 vs. PC2 and **B**) is after GHB and GHB fragments are excluded from the data. **C**) PCA of baseline and 6-hour samples showing PC1 vs. PC2 and **D**) is after GHB and GHB fragments are excluded from the data. **E**) PCA of baseline and 24-hour samples showing PC1 vs. PC2 and **F**) PC3 vs. PC4. Samples are coloured by time point: baseline (light red) and post-administration (dark red). The percentage of total variance explained by each component is indicated in parentheses on the axis labels.

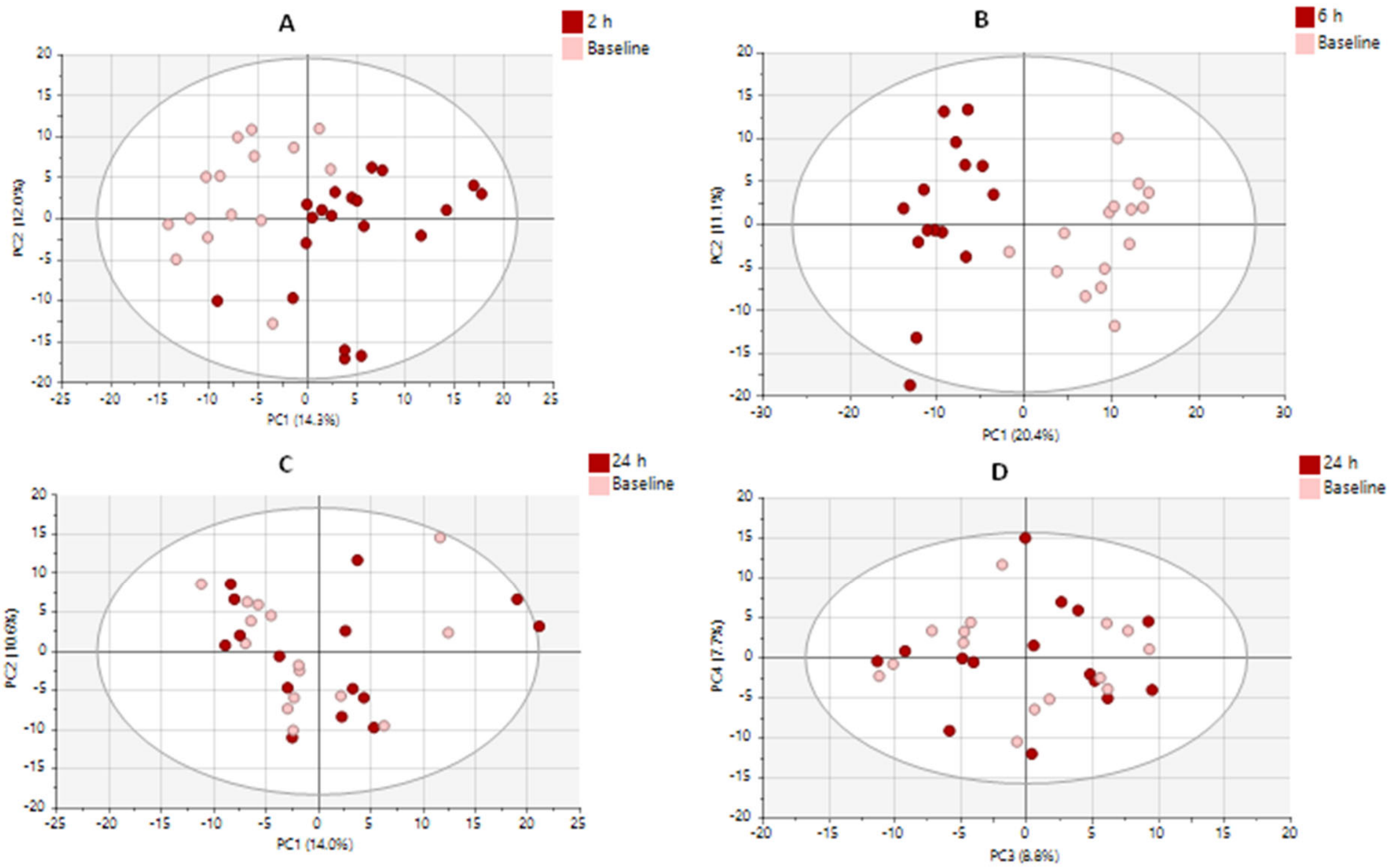

Fig. S7. PCA scores plots of urine samples from the GHB treatment group, acquired in negative electrospray ionisation (ESI) mode (normalized data). A) PCA of baseline and 2-hour samples showing PC1 vs. PC2. **B**) PCA of baseline and 6-hour samples showing PC1 vs. PC2. **C**) PCA of baseline and 24-hour samples showing PC1 vs. PC2 and **D**) PC3 vs. PC4. Samples are coloured by time point: baseline (light red) and post-administration (dark red). The percentage of total variance explained by each component is indicated in parentheses on the axis labels.

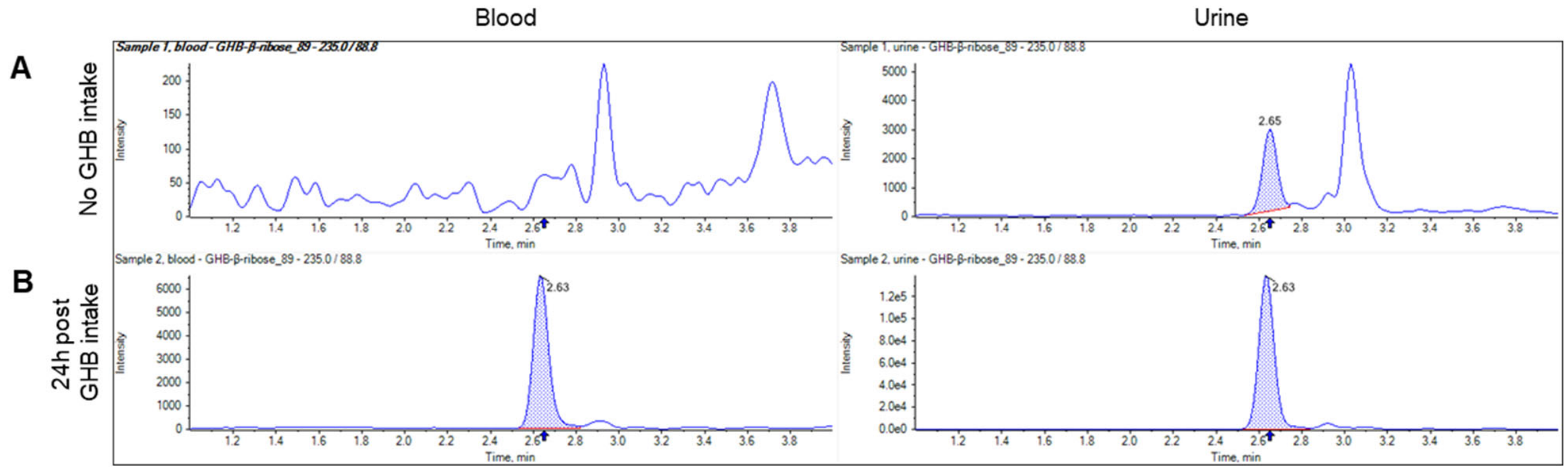

Fig. S8. LC-MS/MS chromatograms of GHB-β-ribose in two blood/urine pairs. A) An authentic "blank" blood/urine sample pair with no detectable concentration of GHB-β-ribose in blood and 0.005 µg/mL GHB-β-ribose in urine. **B**) A blood/urine sample pair 24h post GHB intake from the clinical study containing 0.012 µg/mL and 0.36 µg/mL GHB-β-ribose in blood and urine, respectively. The arrows indicate the expected retention time of GHB-β-ribose.

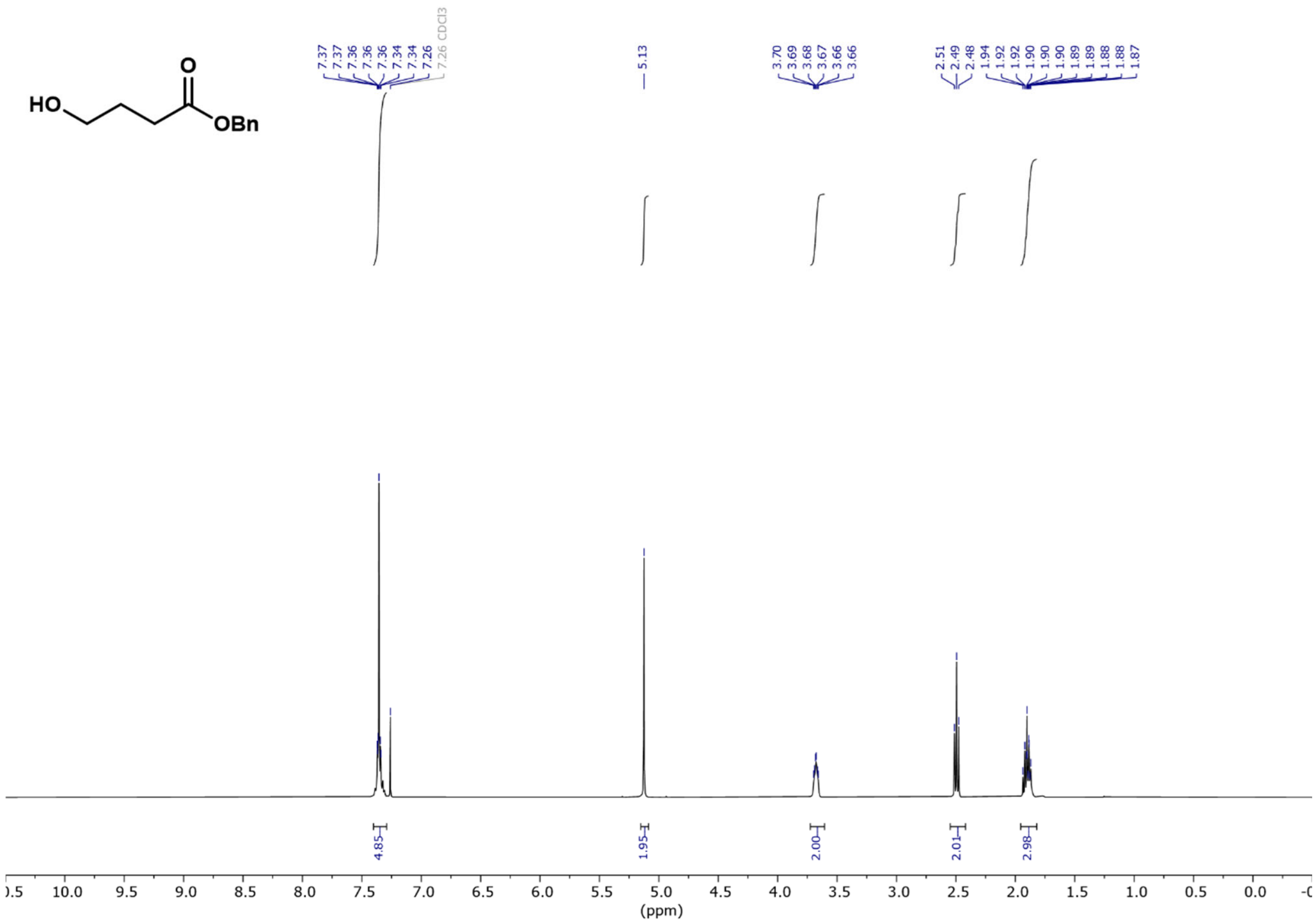

Fig. S9. ^{1}H-NMR (400 MHz, $CDCl_3$) of Benzyl-GHB.

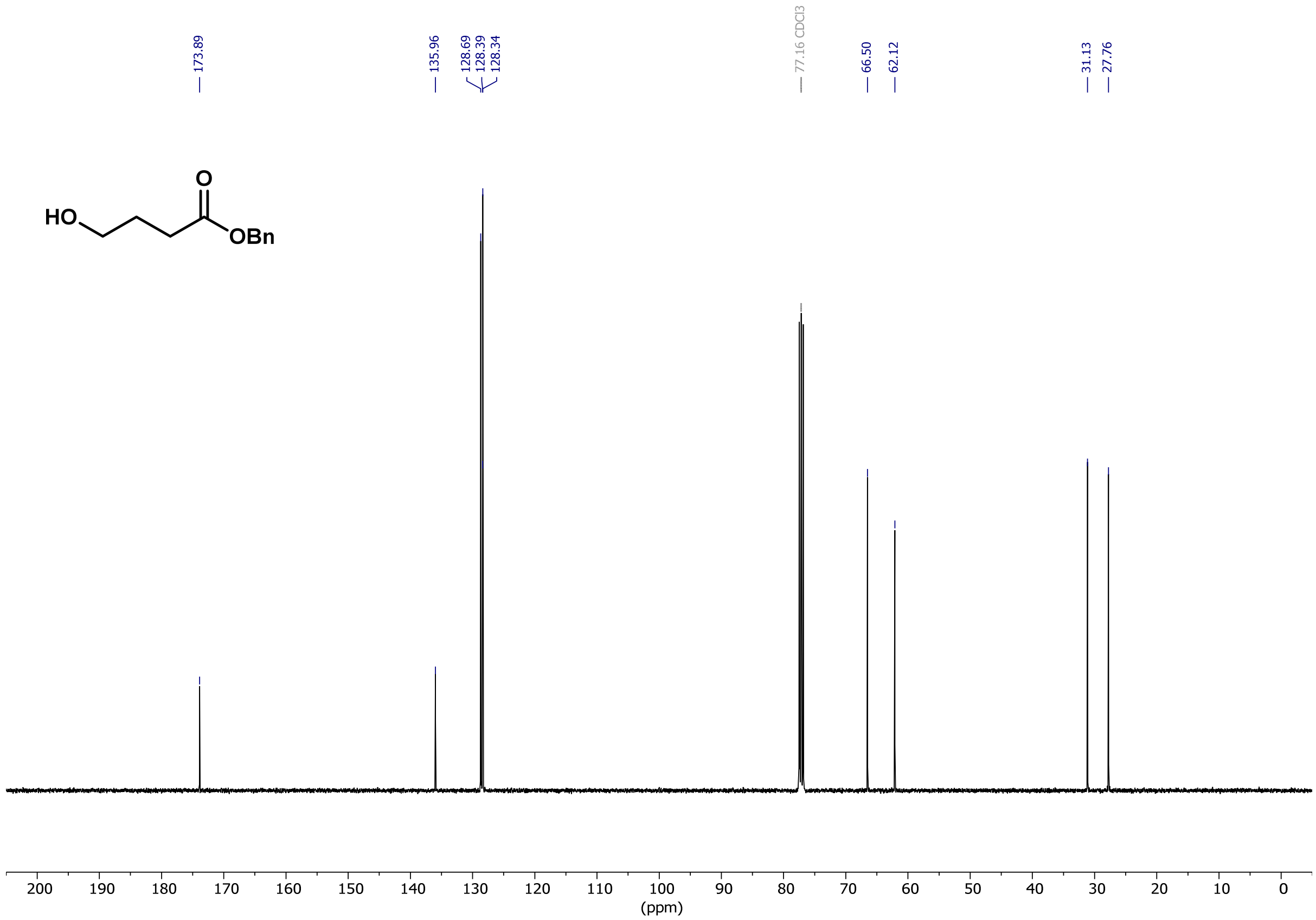

Fig. S10. ^{13}C-NMR (101 MHz, $CDCl_3$) of Benzyl-GHB.

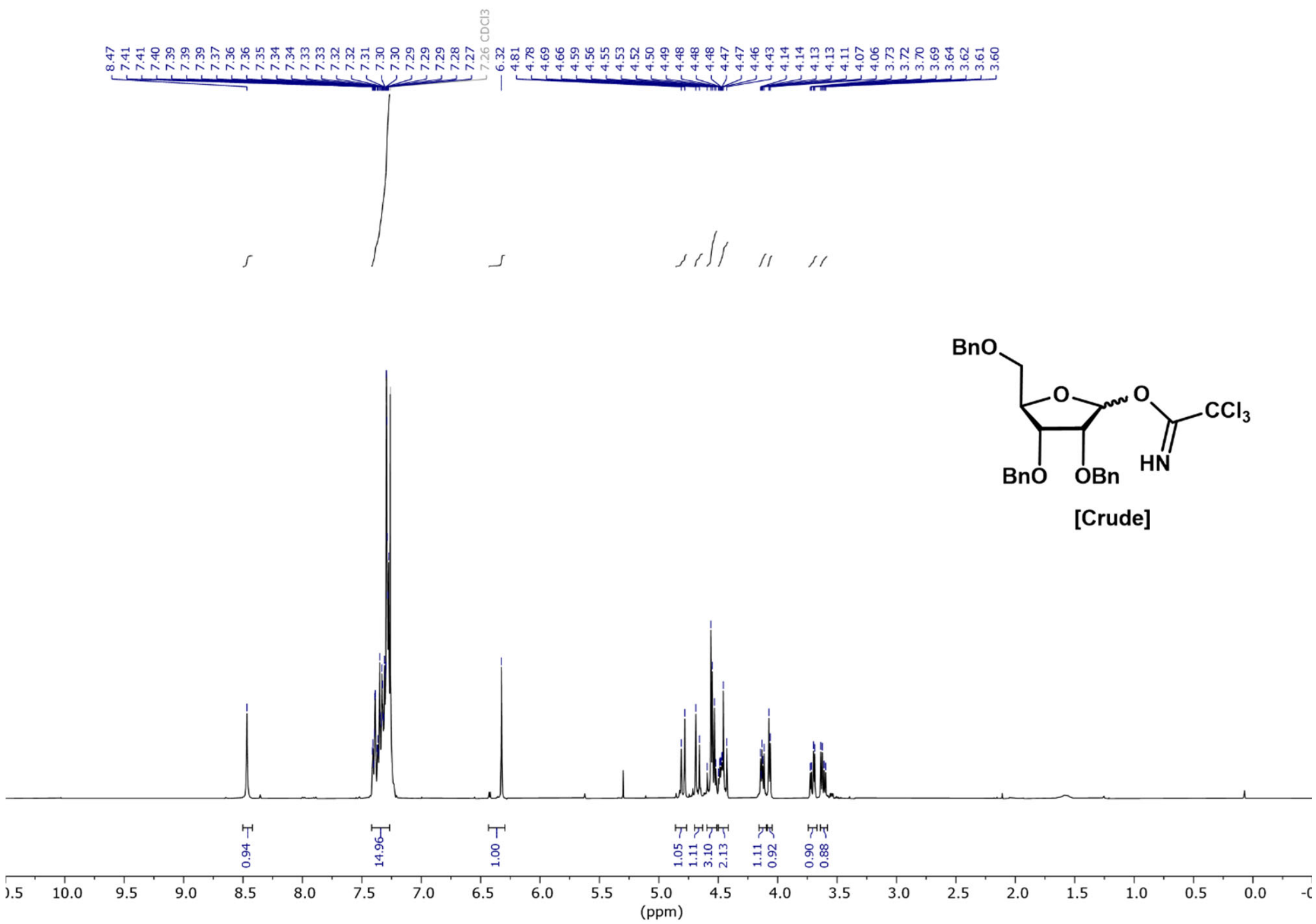

Fig. S11. ^{1}H-NMR (400 MHz, $CDCl_3$) of crude activated perbenzylated ribose.

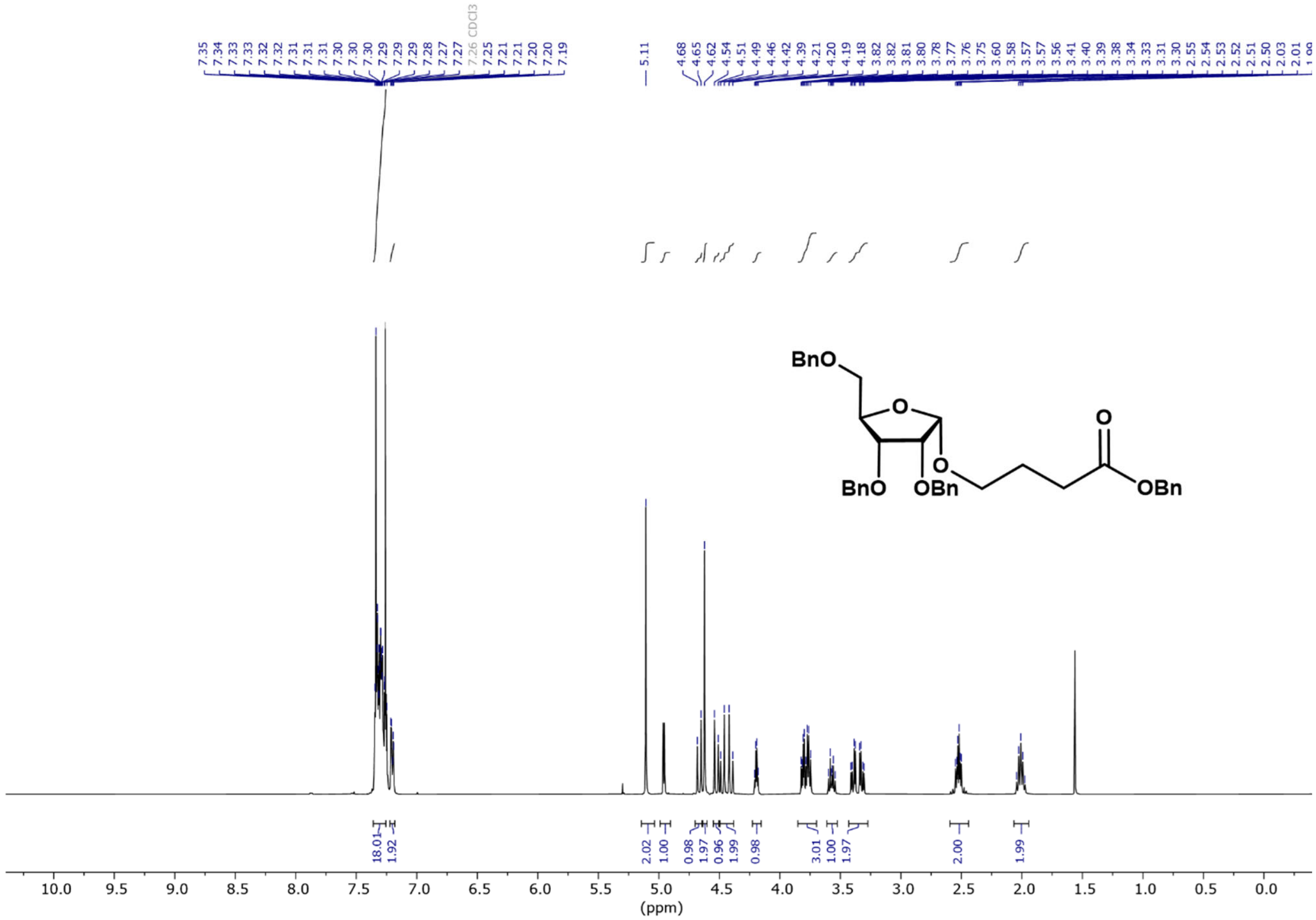

Fig. S12. ^{1}H-NMR (400 MHz, $CDCl_3$) of α-coupling product.

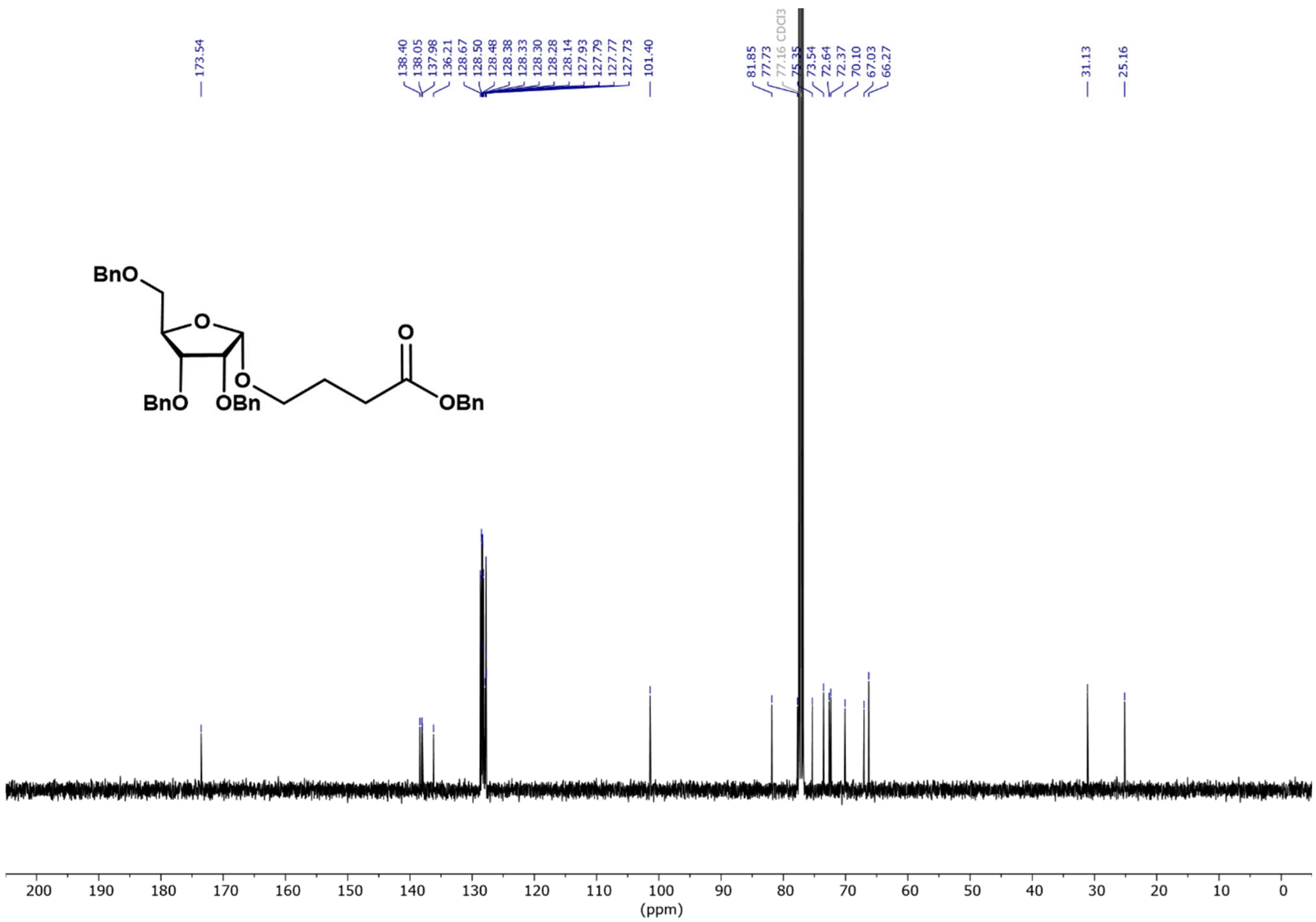

Fig. S13. ^{13}C-NMR (101 MHz, $CDCl_3$) of α-coupling product.

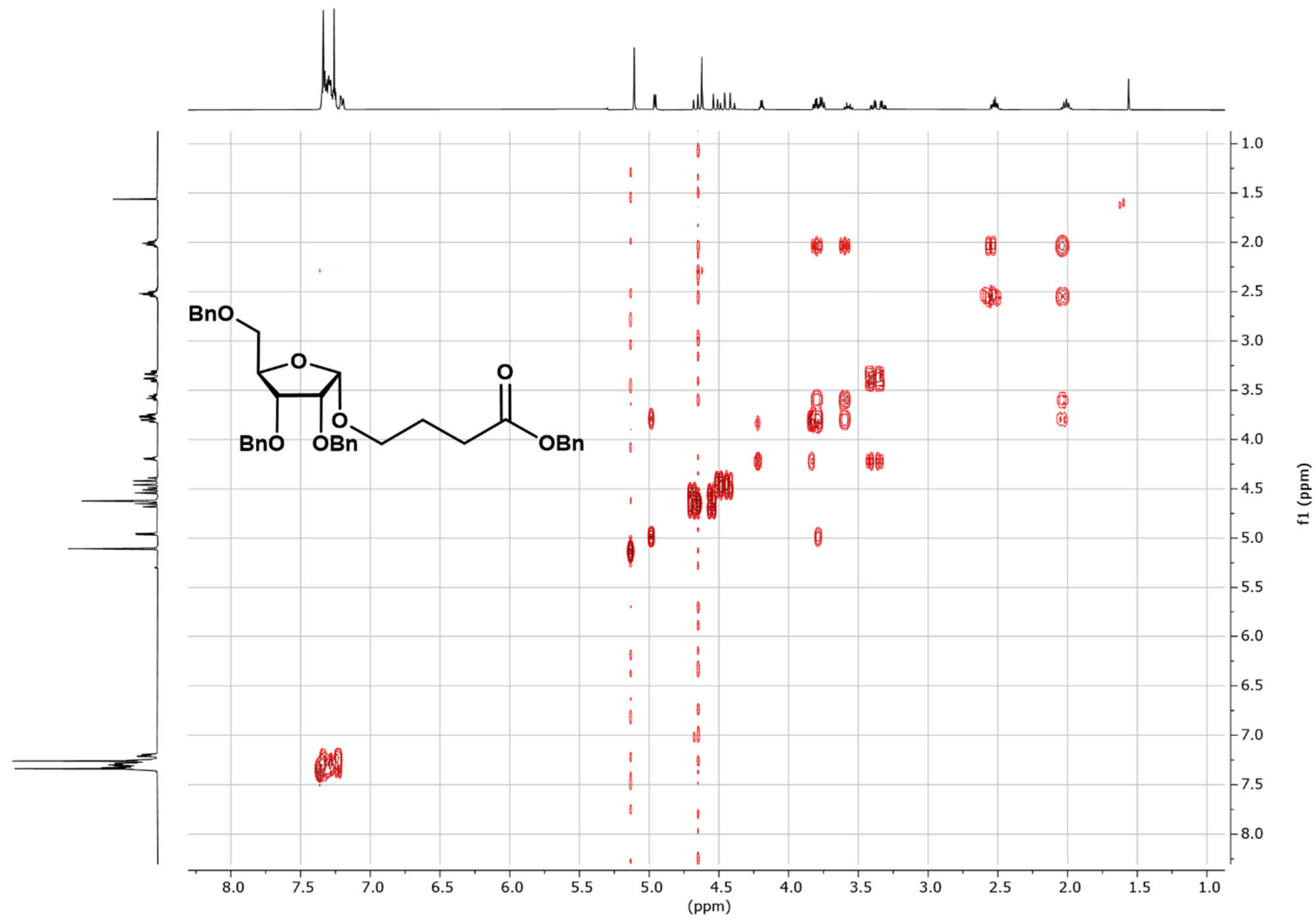

Fig. S14. ^{1}H-^{1}H COSY (400 MHz, $CDCl_3$) of α-coupling product.

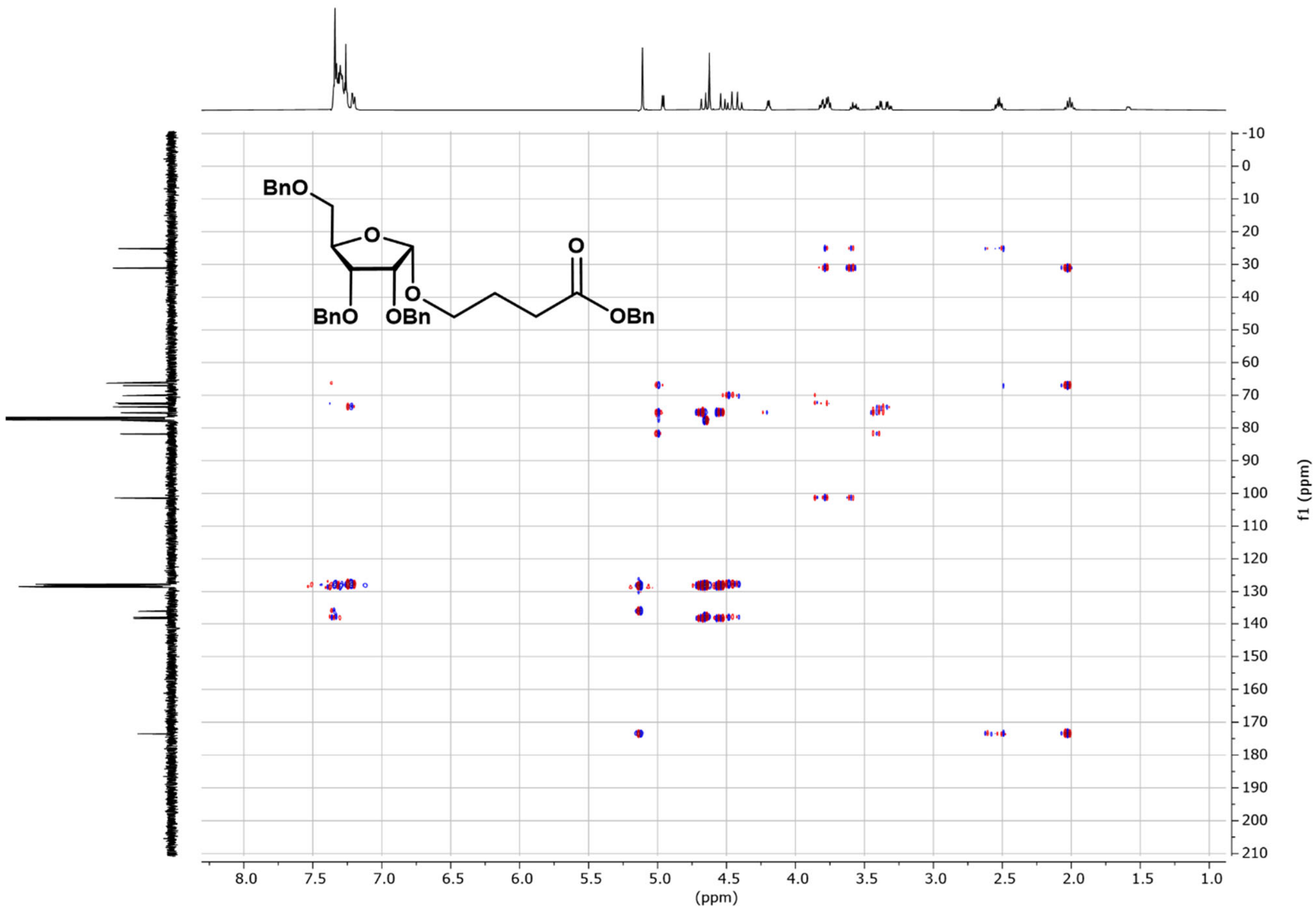

Fig. S15. **^{1}H-^{13}C HMBC (400 MHz, $CDCl_3$) of α-coupling product.**

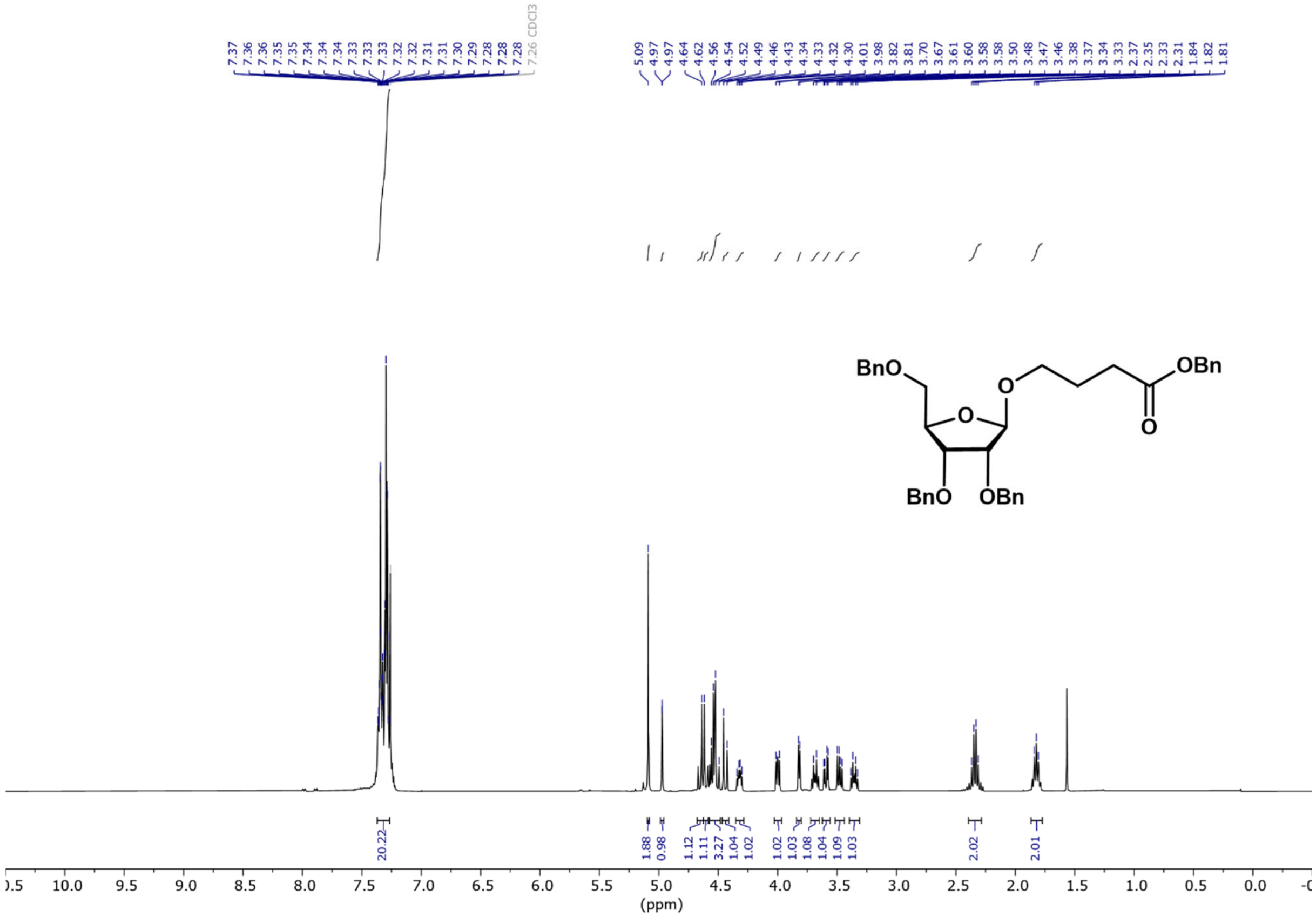

Fig. S16. ^{1}H-NMR (400 MHz, $CDCl_3$) of β-coupling product.

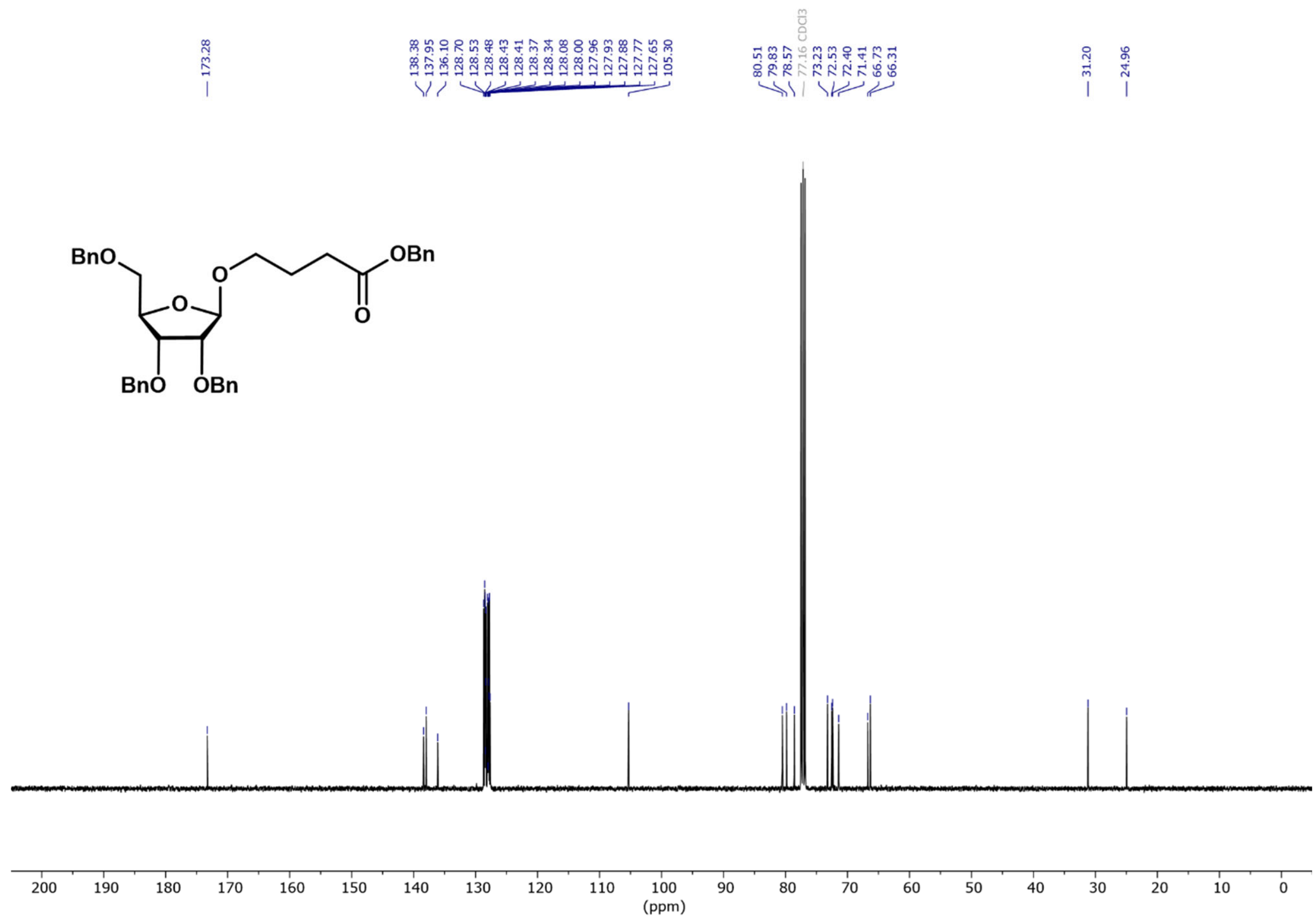

Fig. S17. **^{13}C-NMR (101 MHz, $CDCl_3$) of β-coupling product.**

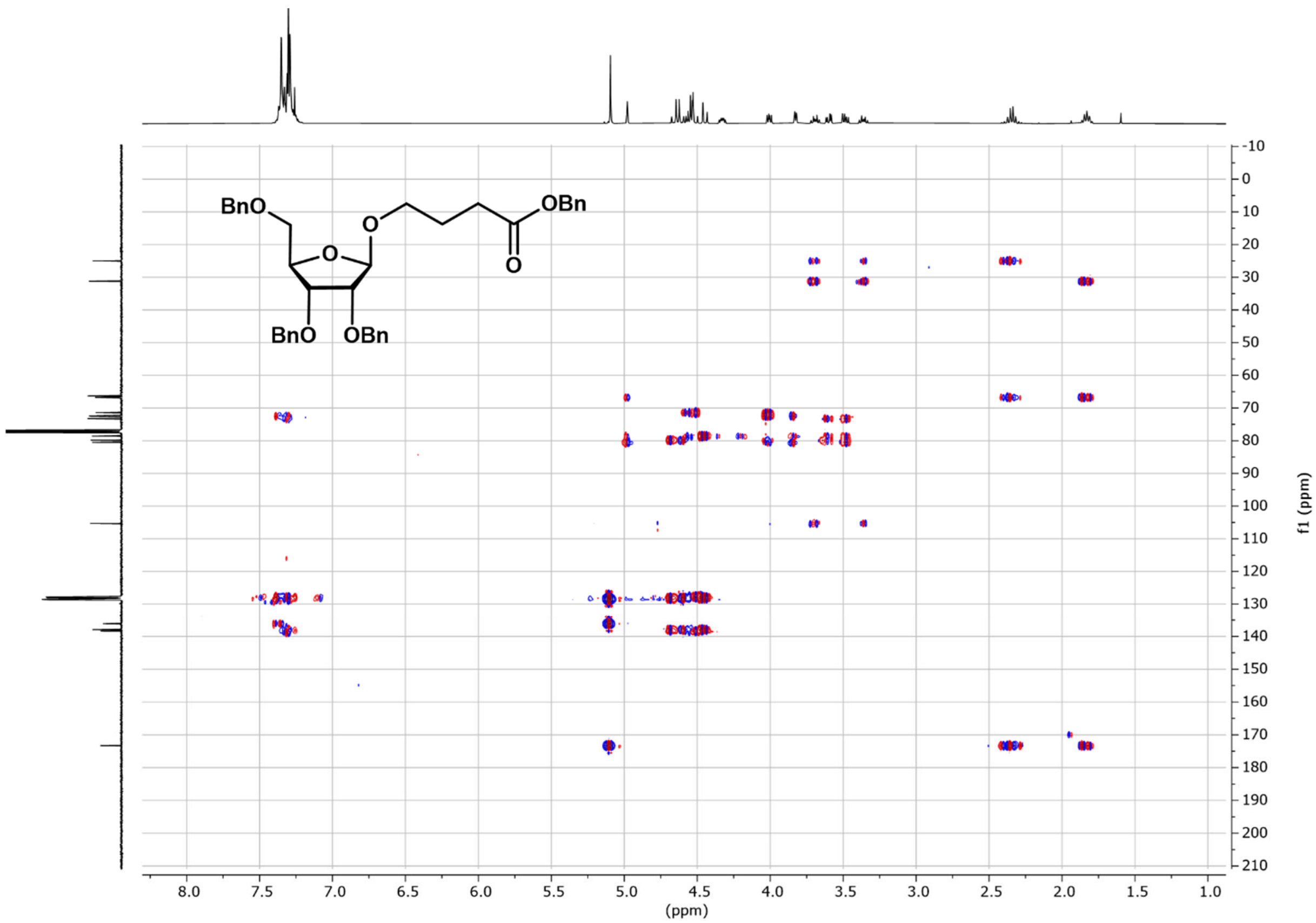

Fig. S18. ^{1}H-^{13}C HMBC (400 MHz, $CDCl_3$) of β-coupling product.

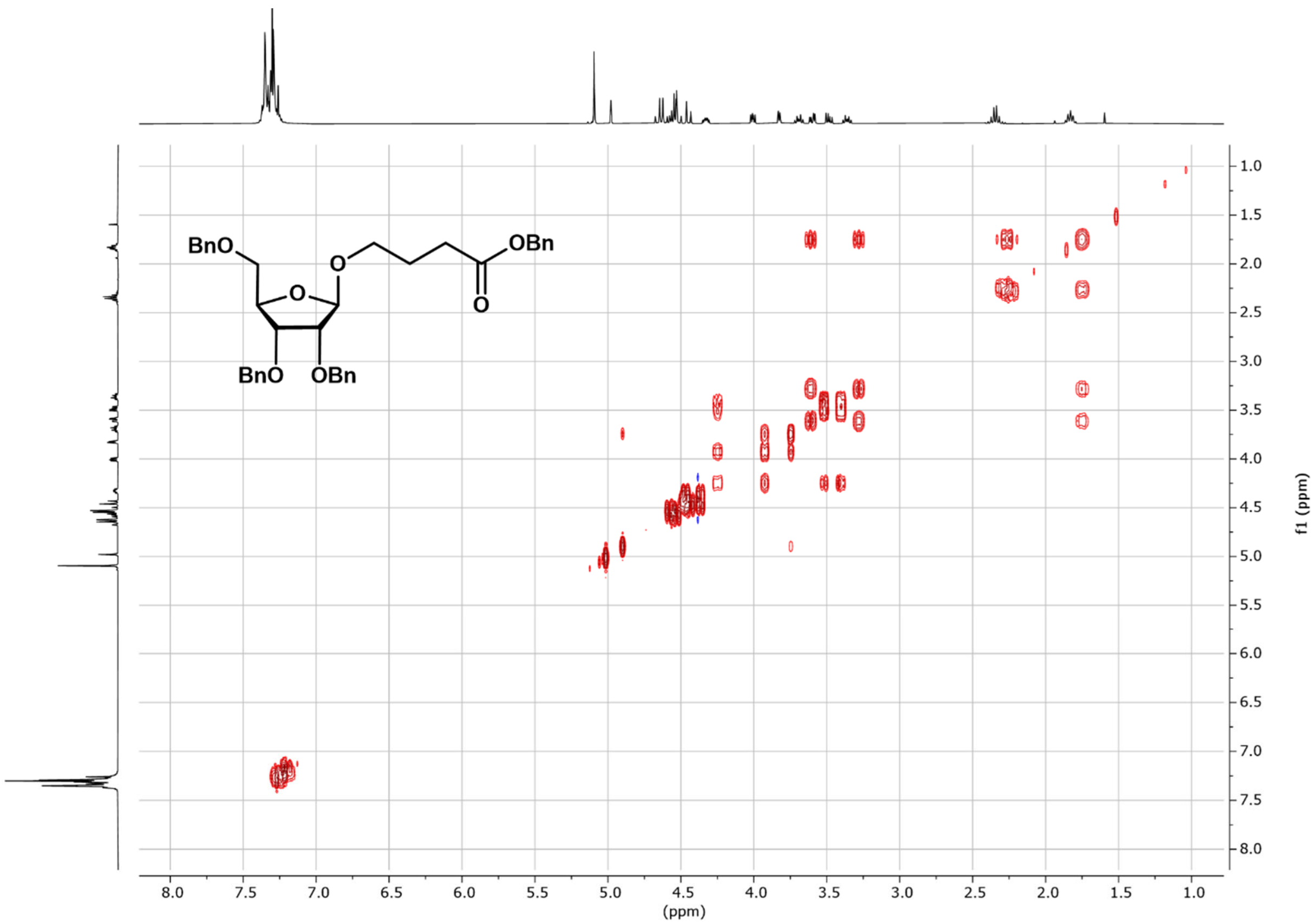

Fig. S19. ^{1}H-^{1}H COSY (400 MHz, $CDCl_3$) of β-coupling product.

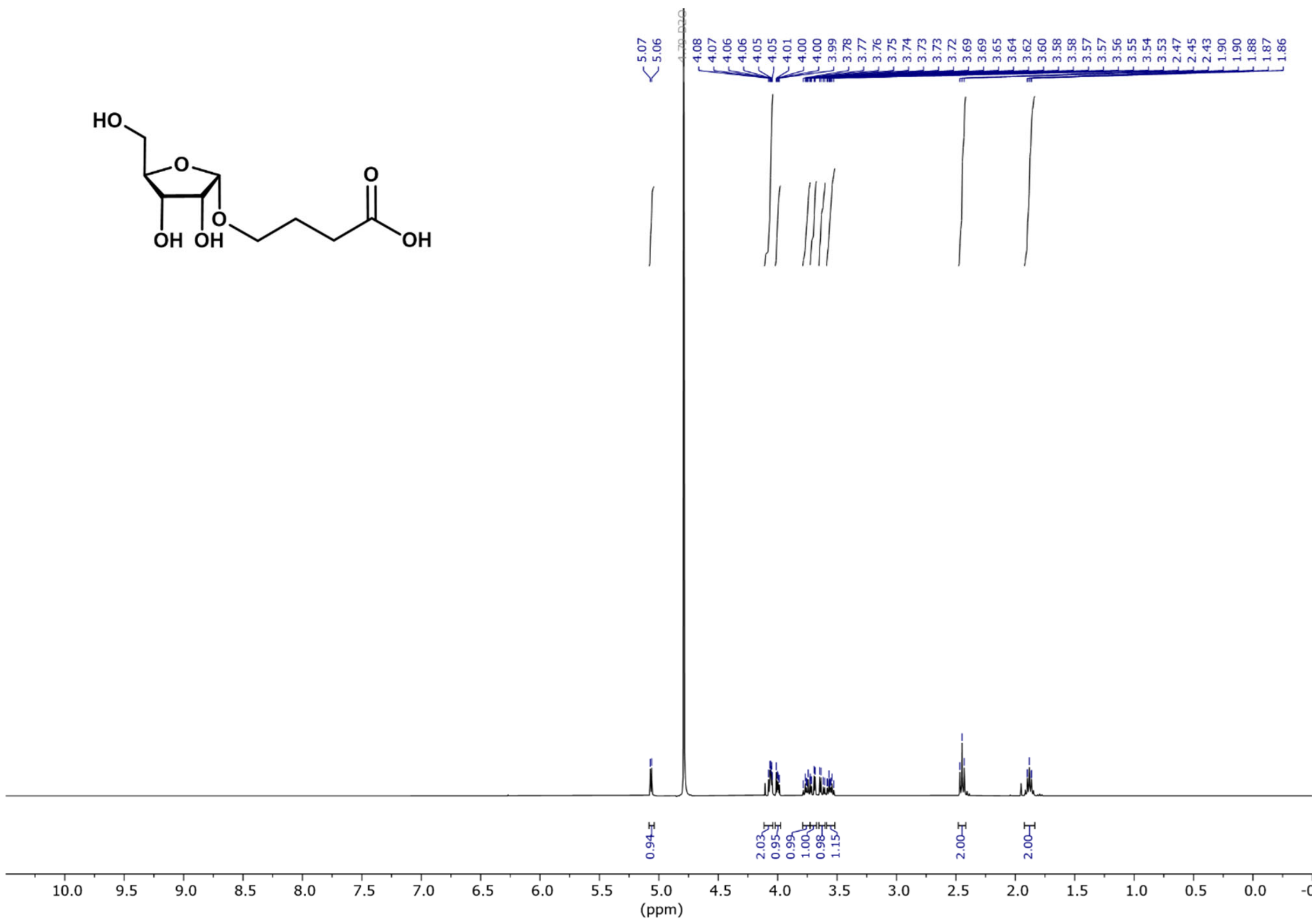

Fig. S20. ^{1}H-NMR (400 MHz, D_2O) of GHB-α-ribose.

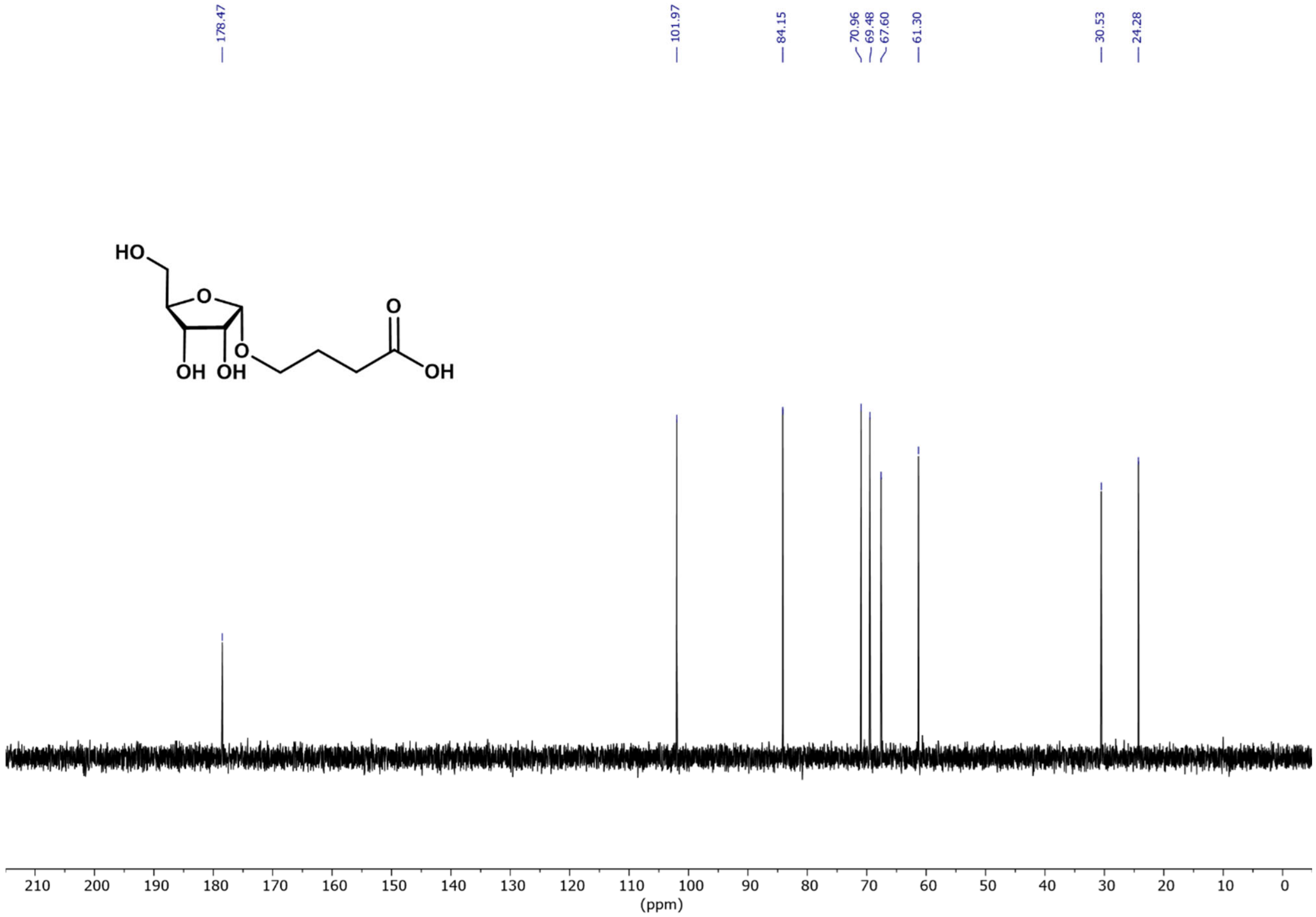

Fig. S21. ^{13}**C-NMR (101 MHz,** D_2O**) of GHB-α-ribose.**

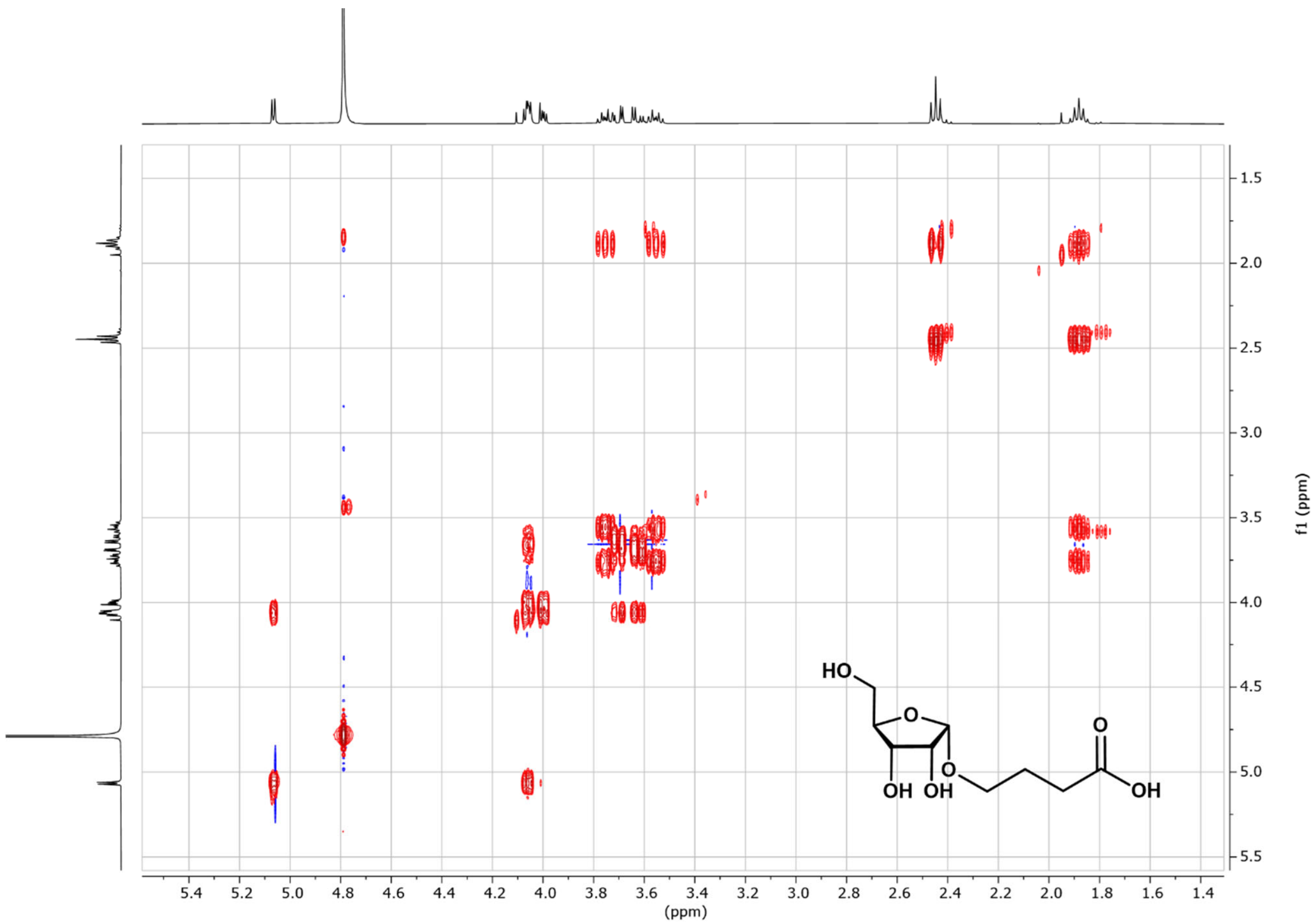

Fig. S22. ^{1}H-^{1}H COSY (400 MHz, D_2O) of GHB-α-ribose.

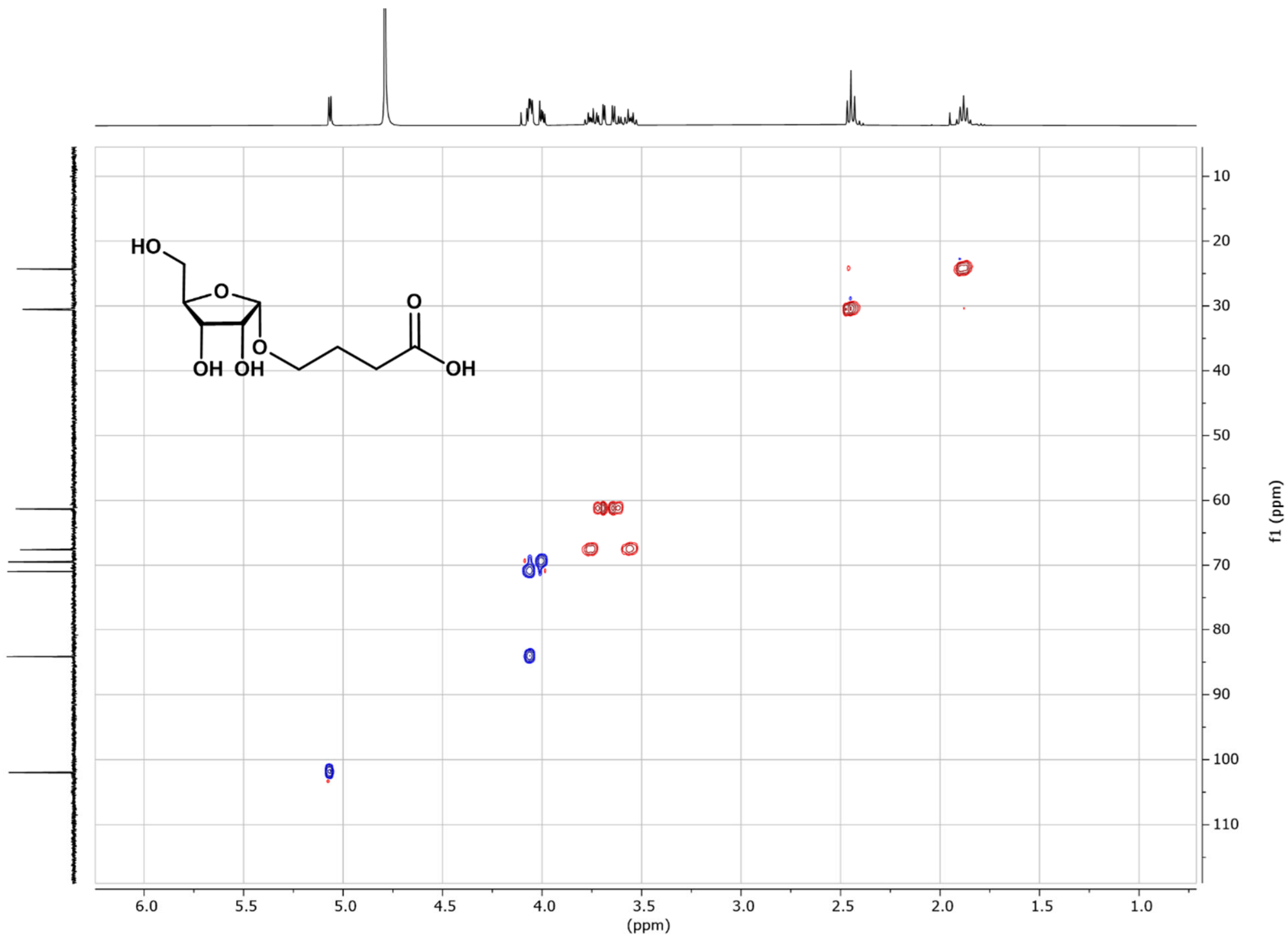

Fig. S23. ^{1}H-^{13}C HSQC (400 MHz, D_2O) of GHB-α-ribose.

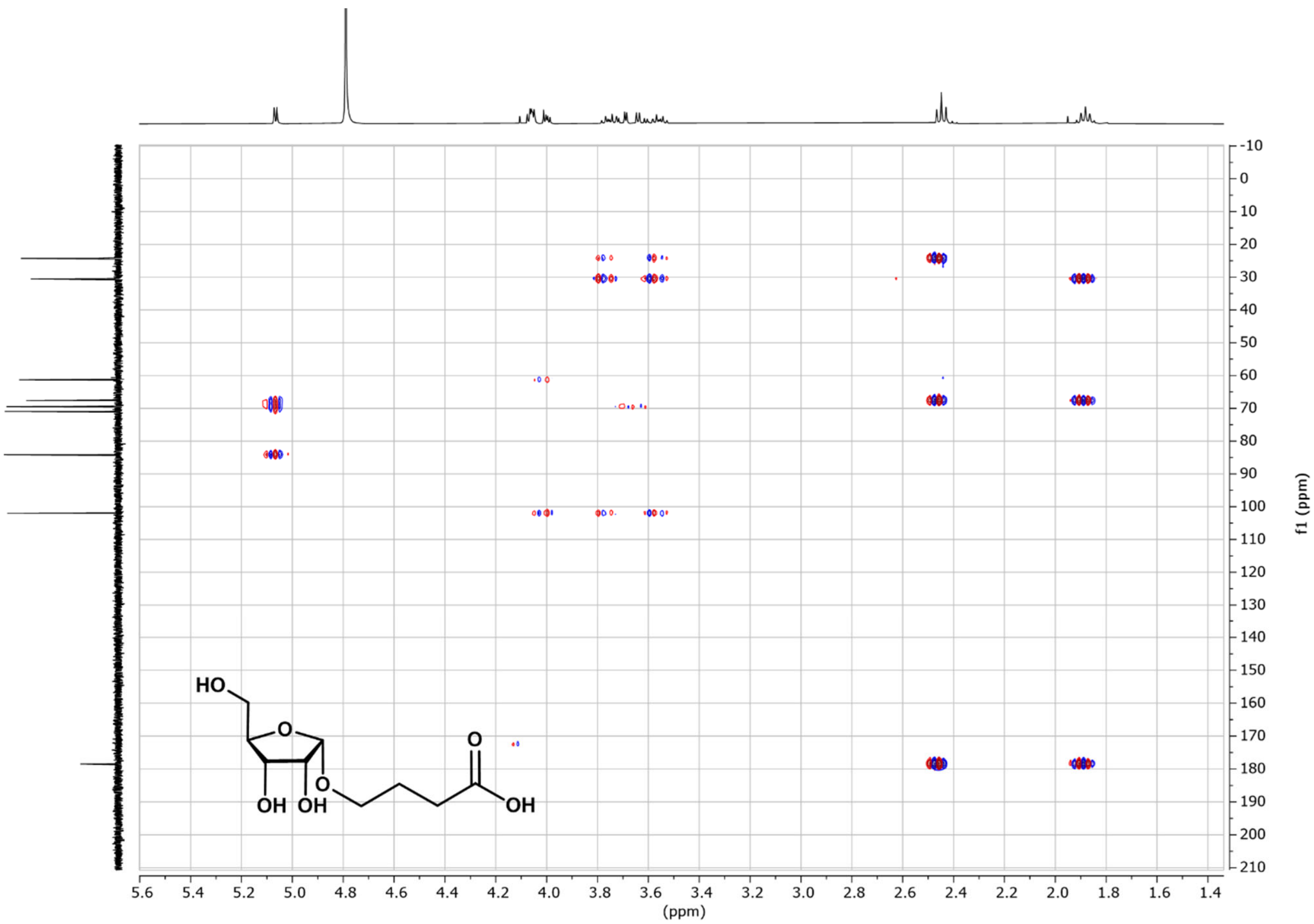

Fig. S24. ^{1}H-^{13}C **HMBC (400 MHz,** D_2O**) of GHB-α-ribose.**

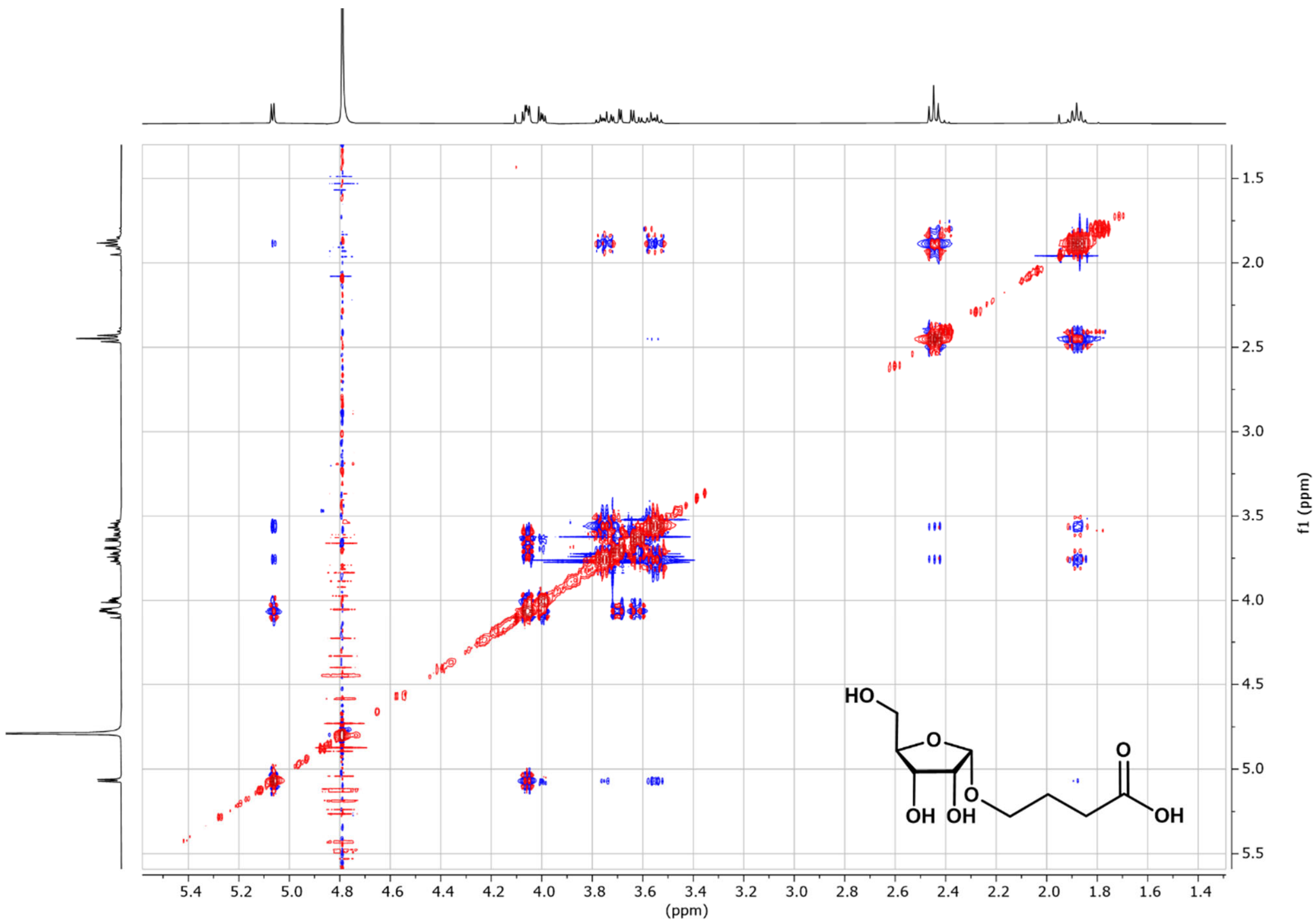

Fig. S25. ^{1}H-^{1}H NOESY (400 MHz, D_2O) of GHB-α-ribose.

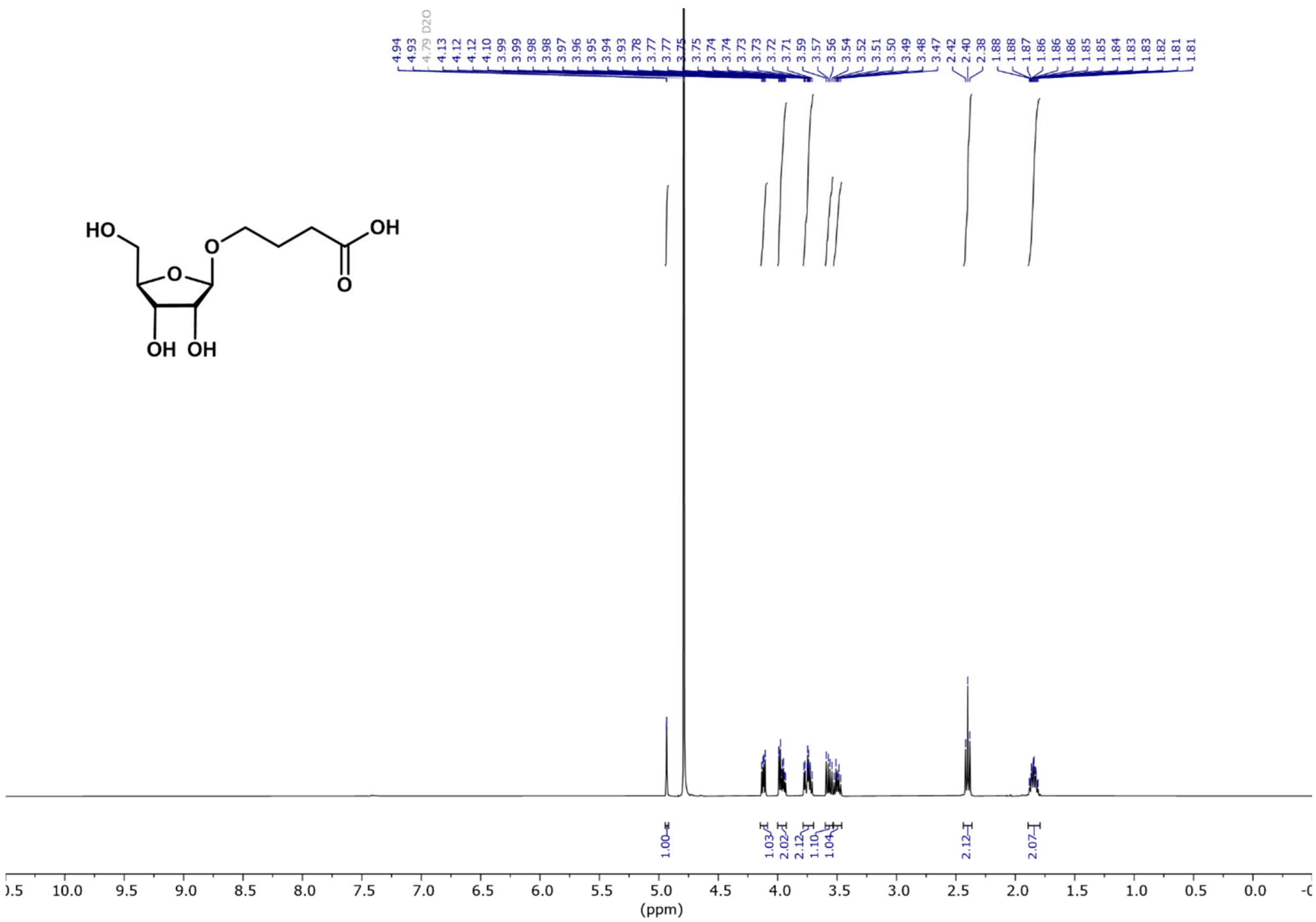

Fig. S26. ^{1}H-NMR (400 MHz, D_2O) of GHB-β-ribose.

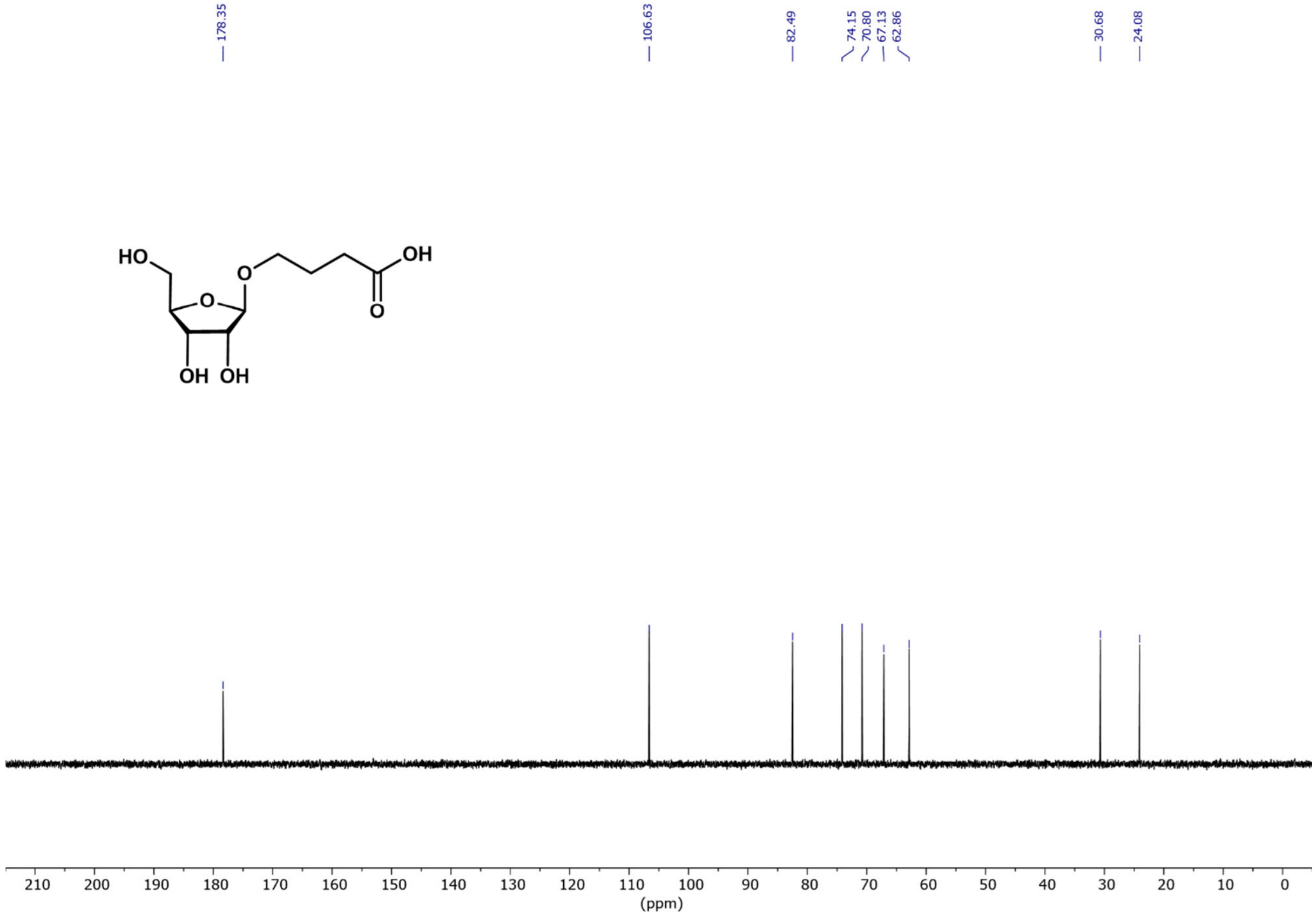

Fig. S27. ^{13}C-NMR (101 MHz, D_2O) of GHB-β-ribose.

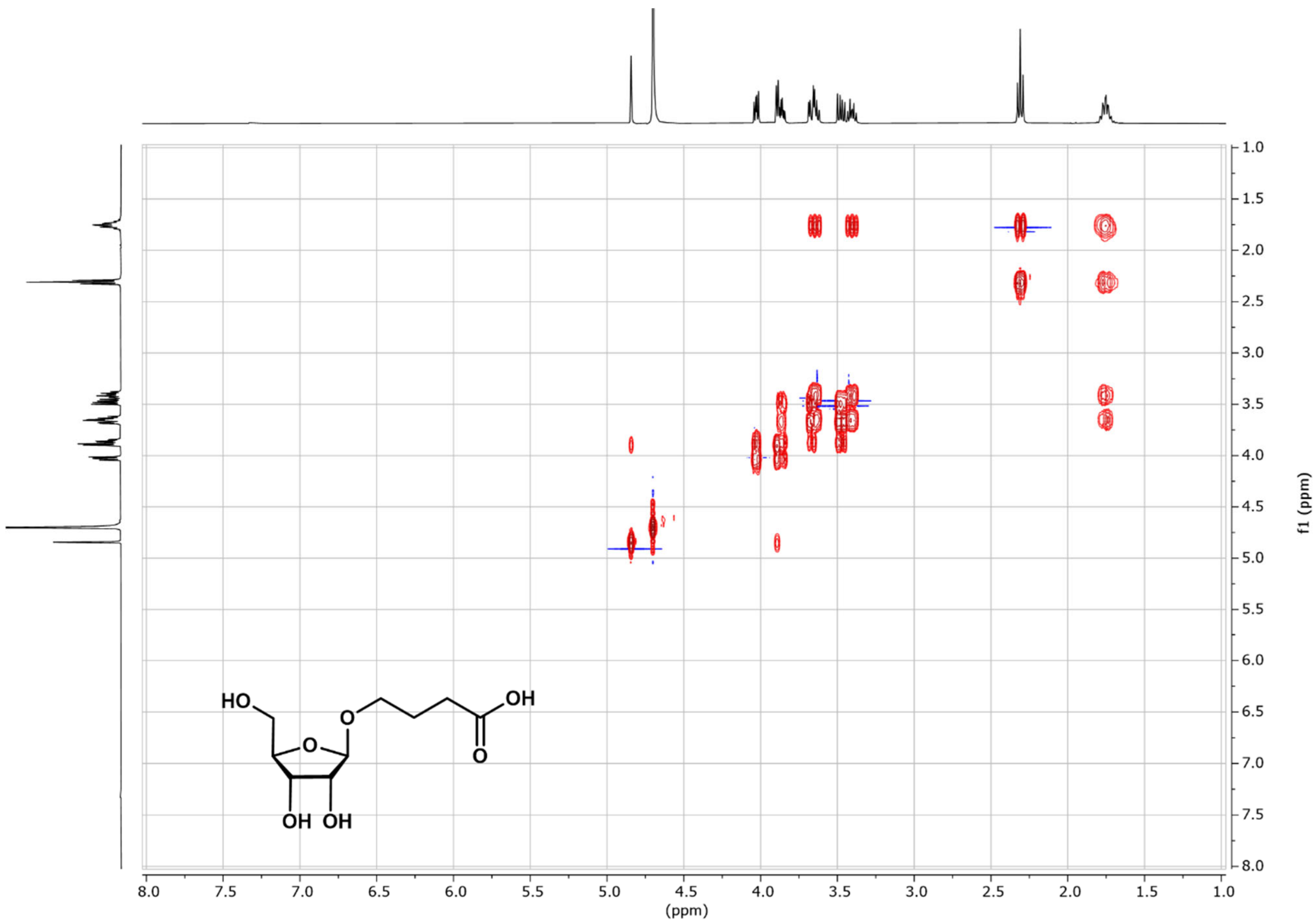

Fig. S28. ^{1}H-^{1}H **COSY (400 MHz,** D_2O**) of GHB-β-ribose.**

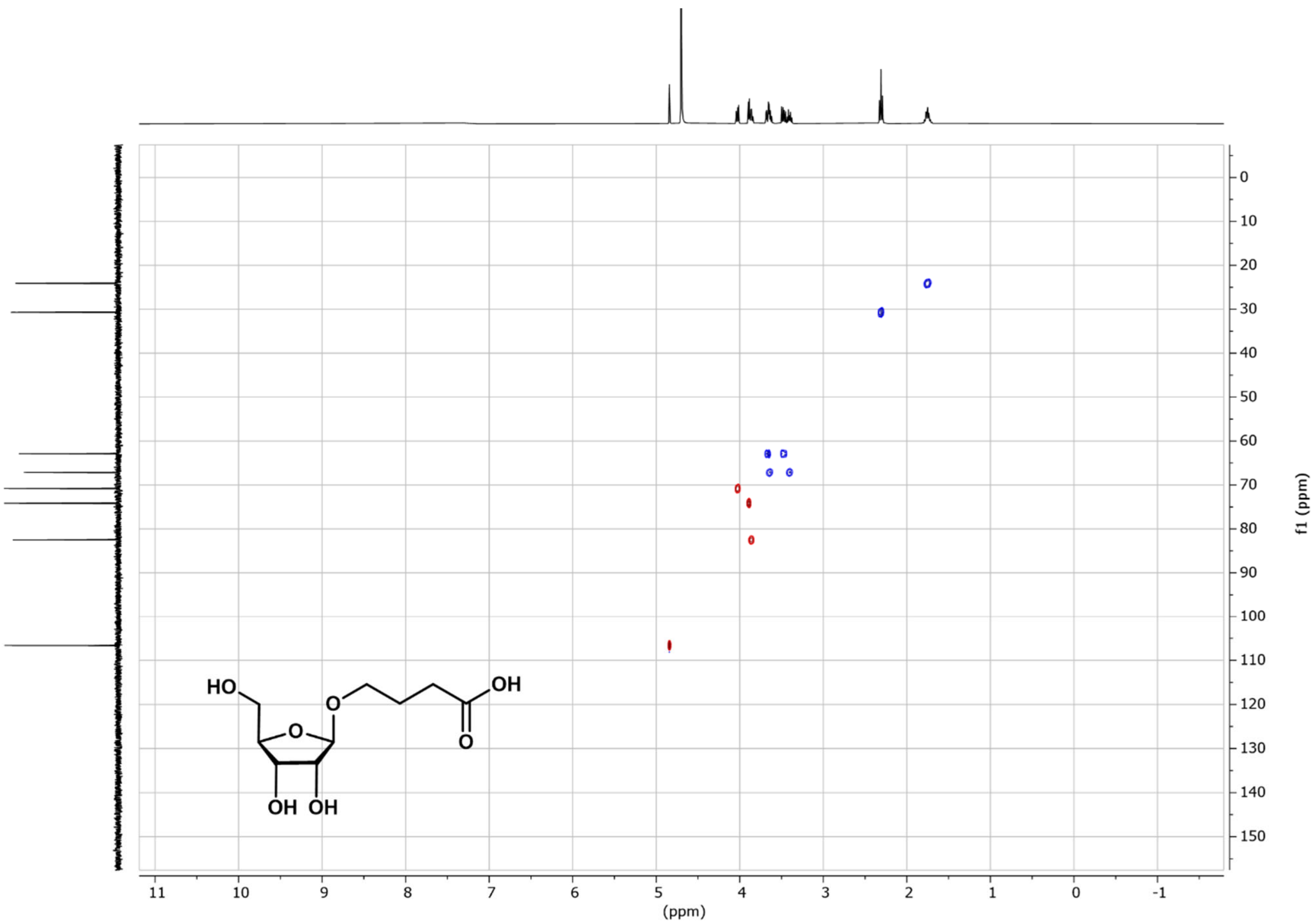

Fig. S29. ^{1}H-^{13}C **HSQC (400 MHz,** D_2O**) of GHB-β-ribose.**

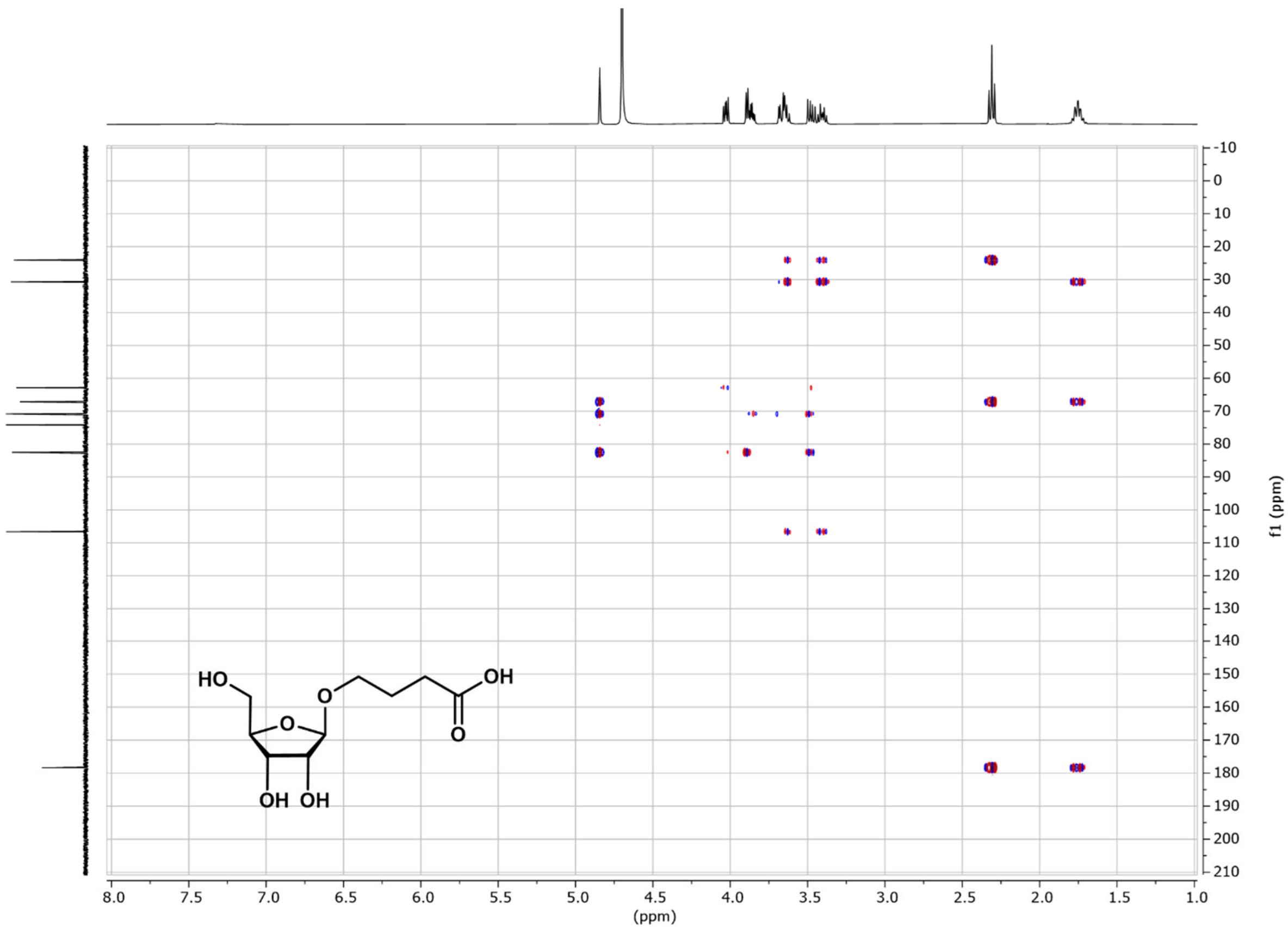

Fig. S30. **^{1}H-^{13}C HMBC (400 MHz, D_2O) of GHB-β-ribose.**

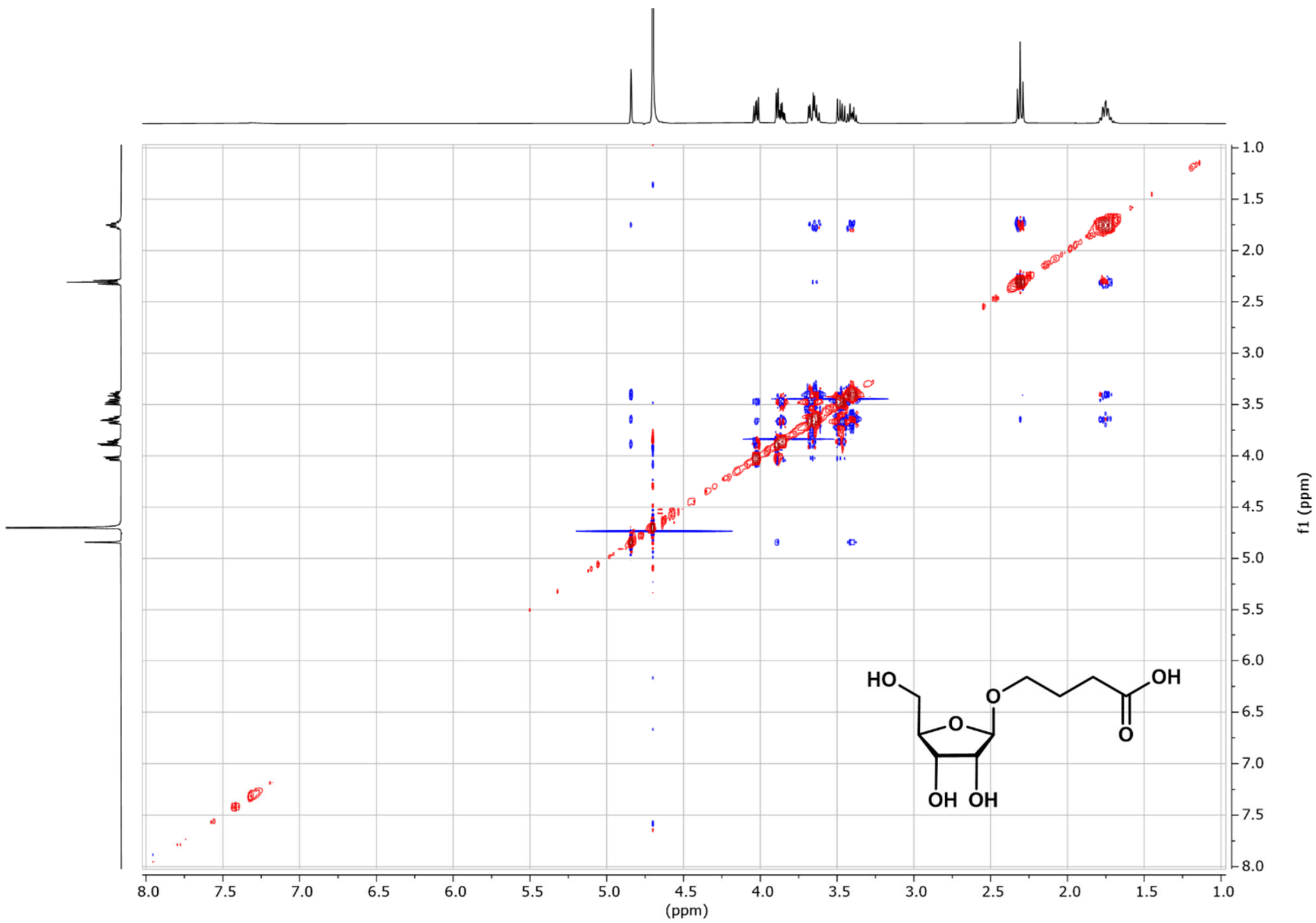

Fig. S31. ^{1}H-^{1}H NOESY (400 MHz, D_2O) of GHB-β-ribose.

Table S1. Features detected in urine and blood in positive or negative electrospray ionisation (ESI) mode that exhibited consistent and significantly increased or decreased fold change (fc) at 2 h, 4 h, and 6 h compared to baseline. Features are sorted by retention time (RT) and those presumed to originate from the same compound, based on identical RT, are grouped accordingly. Identification levels follow the Metabolomics Standards Initiative (MSI): Level 1 – confirmed by reference standard; Level 2 – putatively annotated (MS/MS and database); Level 3 – putatively characterised by class; Level 4 – unknown.

**Feature**	**Matrix**	**ESI**	**m/z**	**RT (s)**	**Identifi-cation**	**Level**	**Time**	**fc**	**p value**
M429T31	blood	positive	428.824	31	unknown	4	4 h	0.76	0.039
M429T31	blood	positive	428.824	31	unknown	4	6 h	0.80	0.019
M429T31	blood	positive	428.824	31	unknown	4	12 h	0.93	0.29
M597T45	blood	negative	597.095	45	unknown	4	4 h	1.38	0.0017
M597T45	blood	negative	597.095	45	unknown	4	6 h	1.22	0.016
M597T45	blood	negative	597.095	45	unknown	4	12 h	1.14	0.18
M599T45	blood	negative	599.093	45	unknown	4	4 h	1.29	0.0081
M599T45	blood	negative	599.093	45	unknown	4	6 h	1.26	0.011
M599T45	blood	negative	599.093	45	unknown	4	12 h	1.25	0.09
M207T46	urine	positive	207.031	46	unknown	4	2 h	1.48	0.0074
M207T46	urine	positive	207.031	46	unknown	4	4 h	1.66	<0.0001
M207T46	urine	positive	207.031	46	unknown	4	6 h	1.64	0.0018
M207T46	urine	positive	207.031	46	unknown	4	12 h	0.71	0.0052
M59T49	urine	negative	59.0133	49	DHB-fragment (C2H3O2)	2	2 h	5.19	<0.0001
M59T49	urine	negative	59.0133	49	DHB-fragment (C2H3O2)	2	4 h	5.46	<0.0001
M59T49	urine	negative	59.0133	49	DHB-fragment (C2H3O2)	2	6 h	3.17	<0.0001
M59T49	urine	negative	59.0133	49	DHB-fragment (C2H3O2)	2	12 h	1.30	0.037
M103T49	urine	positive	103.038	49	unknown	4	2 h	3.28	<0.0001
M103T49	urine	positive	103.038	49	unknown	4	4 h	3.40	<0.0001
M103T49	urine	positive	103.038	49	unknown	4	6 h	2.30	<0.0001
M103T49	urine	positive	103.038	49	unknown	4	12 h	0.82	0.0096

M243T71	urine	positive	243.169	71	unknown	4	2 h	7.94	<0.0001
M243T71	urine	positive	243.169	71	unknown	4	4 h	3.52	<0.0001
M243T71	urine	positive	243.169	71	unknown	4	6 h	2.64	<0.0001
M243T71	urine	positive	243.169	71	unknown	4	12 h	0.70	0.11
M249T77	urine	positive	249.152	77	unknown	4	2 h	117.36	<0.0001
M249T77	urine	positive	249.152	77	unknown	4	4 h	41.46	0.002
M249T77	urine	positive	249.152	77	unknown	4	6 h	7.16	0.041
M249T77	urine	positive	249.152	77	unknown	4	12 h	0.92	0.48
M76T81	urine	positive	76.039	81	unknown	4	2 h	11.73	<0.0001
M76T81	urine	positive	76.039	81	unknown	4	4 h	4.92	<0.0001
M76T81	urine	positive	76.039	81	unknown	4	6 h	2.29	<0.0001
M76T81	urine	positive	76.039	81	unknown	4	12 h	0.92	0.26
M85T81	urine	positive	85.028	81	unknown	4	2 h	3.44	0.025
M85T81	urine	positive	85.028	81	unknown	4	4 h	2.03	0.0058
M85T81	urine	positive	85.028	81	unknown	4	6 h	1.50	0.045
M85T81	urine	positive	85.028	81	unknown	4	12 h	0.86	0.16
M248T88	urine	positive	248.148	88	GHB-carnitine	1	2 h	3.81	<0.001
M248T88	urine	positive	248.148	88	GHB-carnitine	1	4 h	2.92	0.0012
M248T88	urine	positive	248.148	88	GHB-carnitine	1	6 h	1.93	0.018
M248T88	urine	positive	248.148	88	GHB-carnitine	1	12 h	0.82	0.50
M45T93	urine	positive	45.032	93	GHB fragment (C2H5O)	1	2 h	18.34	<0.0001
M45T93	urine	positive	45.032	93	GHB fragment (C2H5O)	1	4 h	5.15	<0.0001
M45T93	urine	positive	45.032	93	GHB fragment (C2H5O)	1	6 h	1.57	0.015
M45T93	urine	positive	45.032	93	GHB fragment (C2H5O)	1	12 h	1.00	1

M69T93	urine	positive	69.033	93	GHB fragment (C4H5O)	1	2 h	3.22	<0.0001
M69T93	urine	positive	69.033	93	GHB fragment (C4H5O)	1	4 h	1.59	<0.001
M69T93	urine	positive	69.033	93	GHB fragment (C4H5O)	1	6 h	1.38	0.049
M69T93	urine	positive	69.033	93	GHB fragment (C4H5O)	1	12 h	0.90	0.31
M87T93	urine	positive	87.044	93	GHB fragment (C4H7O2)	1	2 h	36.44	<0.0001
M87T93	urine	positive	87.044	93	GHB fragment (C4H7O2)	1	4 h	10.51	<0.0001
M87T93	urine	positive	87.044	93	GHB fragment (C4H7O2)	1	6 h	3.20	0.0045
M87T93	urine	positive	87.044	93	GHB fragment (C4H7O2)	1	12 h	0.86	0.53
M105T93	urine	positive	105.054	93	GHB	1	2 h	43.22	<0.0001
M105T93	urine	positive	105.054	93	GHB	1	4 h	6.91	<0.001
M105T93	urine	positive	105.054	93	GHB	1	6 h	1.41	0.19
M105T93	urine	positive	105.054	93	GHB	1	12 h	0.95	0.64
M72T96	urine	positive	72.044	96	unknown	4	2 h	1.73	<0.001
M72T96	urine	positive	72.044	96	unknown	4	4 h	1.48	0.035
M72T96	urine	positive	72.044	96	unknown	4	6 h	1.38	0.035
M72T96	urine	positive	72.044	96	unknown	4	12 h	0.87	0.39
M90T96	urine	positive	90.0542	96	unknown	4	2 h	1.74	<0.001
M90T96	urine	positive	90.0542	96	unknown	4	4 h	1.41	0.018
M90T96	urine	positive	90.0542	96	unknown	4	6 h	1.36	0.023
M90T96	urine	positive	90.0542	96	unknown	4	12 h	0.87	0.26
M228T105	urine	positive	228.086	105	unknown	4	2 h	9.12	<0.0001
M228T105	urine	positive	228.086	105	unknown	4	4 h	6.74	<0.0001
M228T105	urine	positive	228.086	105	unknown	4	6 h	4.16	<0.001

M228T105	urine	positive	228.086	105	unknown	4	12 h	0.60	0.056
M183T119	urine	negative	183.03	119	unknown	4	2 h	9.19	<0.0001
M183T119	urine	negative	183.03	119	unknown	4	4 h	8.51	<0.001
M183T119	urine	negative	183.03	119	unknown	4	6 h	5.72	<0.0001
M183T119	urine	negative	183.03	119	unknown	4	12 h	2.18	0.0047
M103T136	urine	negative	103.04	136	unknown	4	2 h	1.50	<0.001
M103T136	urine	negative	103.04	136	unknown	4	4 h	1.54	0.0064
M103T136	urine	negative	103.04	136	unknown	4	6 h	1.89	0.015
M103T136	urine	negative	103.04	136	unknown	4	12 h	1.17	0.23
M97T137	urine	negative	97.029	137	unknown	4	2 h	0.39	<0.0001
M97T137	urine	negative	97.029	137	unknown	4	4 h	0.46	0.0004
M97T137	urine	negative	97.029	137	unknown	4	6 h	0.55	0.0046
M97T137	urine	negative	97.029	137	unknown	4	12 h	1.06	0.36
M174T137	urine	positive	174.123	137	unknown	4	2 h	2.22	0.0038
M174T137	urine	positive	174.123	137	unknown	4	4 h	2.01	0.049
M174T137	urine	positive	174.123	137	unknown	4	6 h	2.38	0.0065
M174T137	urine	positive	174.123	137	unknown	4	12 h	1.11	0.68
M232T146	urine	negative	232.082	146	GHB-glutamate	1	2 h	15.82	<0.0001
M232T146	urine	negative	232.082	146	GHB-glutamate	1	4 h	4.87	<0.0001
M232T146	urine	negative	232.082	146	GHB-glutamate	1	6 h	2.34	<0.0001
M232T146	urine	negative	232.082	146	GHB-glutamate	1	12 h	1.05	0.73
M148T149	urine	positive	148.059	149	unknown	4	2 h	5.99	<0.0001
M148T149	urine	positive	148.059	149	unknown	4	4 h	2.35	<0.0001
M148T149	urine	positive	148.059	149	unknown	4	6 h	1.42	<0.0001
M148T149	urine	positive	148.059	149	unknown	4	12 h	0.97	0.61
M143T150	urine	negative	143.035	150	unknown	4	2 h	7.41	<0.0001
M143T150	urine	negative	143.035	150	unknown	4	4 h	4.90	<0.0001
M143T150	urine	negative	143.035	150	unknown	4	6 h	2.70	<0.0001
M143T150	urine	negative	143.035	150	unknown	4	12 h	1.17	0.22
M174T160	urine	positive	174.123	160	unknown	4	2 h	2.42	0.0023

M174T160	urine	positive	174.123	160	unknown	4	4 h	2.38	0.014
M174T160	urine	positive	174.123	160	unknown	4	6 h	2.81	0.0012
M174T160	urine	positive	174.123	160	unknown	4	12 h	1.17	0.53
M265T163	urine	negative	265.092	163	unknown	4	2 h	4.32	<0.0001
M265T163	urine	negative	265.092	163	unknown	4	4 h	2.89	<0.0001
M265T163	urine	negative	265.092	163	unknown	4	6 h	2.61	<0.0001
M265T163	urine	negative	265.092	163	unknown	4	12 h	1.04	0.77
M259T206	urine	positive	259.074	206	Na-GHB-pentose	2	2 h	2.11	<0.001
M259T206	urine	positive	259.074	206	Na-GHB-pentose	2	4 h	3.54	<0.0001
M259T206	urine	positive	259.074	206	Na-GHB-pentose	2	6 h	1.83	<0.001
M259T206	urine	positive	259.074	206	Na-GHB-pentose	2	12 h	0.94	0.57
M235T207	urine	negative	235.082	207	GHB-pen-tose	2	2 h	43.60	<0.0001
M235T207	urine	negative	235.082	207	GHB-pen-tose	2	4 h	37.62	<0.0001
M235T207	urine	negative	235.082	207	GHB-pen-tose	2	6 h	29.37	<0.0001
M235T207	urine	negative	235.082	207	GHB-pen-tose	2	12 h	8.91	<0.0001
M269T215	urine	positive	269.149	215	unknown	4	2 h	15.38	<0.0001
M269T215	urine	positive	269.149	215	unknown	4	4 h	7.67	<0.0001
M269T215	urine	positive	269.149	215	unknown	4	6 h	4.83	<0.001
M269T215	urine	positive	269.149	215	unknown	4	12 h	0.41	0.0027
M270T215	urine	positive	270.15	215	unknown	4	2 h	2.67	<0.0001
M270T215	urine	positive	270.15	215	unknown	4	4 h	1.63	0.043
M270T215	urine	positive	270.15	215	unknown	4	6 h	1.59	0.0019
M270T215	urine	positive	270.15	215	unknown	4	12 h	0.99	0.88
M334T339	urine	positive	334.091	339	unknown	4	2 h	2.93	0.0031
M334T339	urine	positive	334.091	339	unknown	4	4 h	2.00	0.0087
M334T339	urine	positive	334.091	339	unknown	4	6 h	1.97	0.033
M334T339	urine	positive	334.091	339	unknown	4	12 h	0.93	0.77
M267T518	urine	negative	267.123	518	unknown	4	2 h	2.88	<0.001

M267T518	urine	negative	267.123	518	unknown	4	4 h	1.98	0.043
M267T518	urine	negative	267.123	518	unknown	4	6 h	2.14	0.038
M267T518	urine	negative	267.123	518	unknown	4	12 h	1.50	0.11

Table S2. Mass spectrometric conditions for the analysis of GHB-β-ribose in negative ESI mode. The bold ions were used as quantifiers, and the underlined ions were used as primary qualifiers. Entrance potential (EP) and collision cell exit potential (CXP) were set at 14 V and 10 V, respectively.

Substance	Transition		DP, V	CE, eV	Relative Abundance [a]	Rt, min
	Q1 *m/z*	Q3 *m/z*				
GHB-β-ribose	235	149/131/119/113/ 103/**89**/71/59	25/22/27/25/ 17/22/22/22	15/15/15/19/ 20/19/24/38	15/8.3/4.5/5.4/ 6.4/100/21/68 [b]	2.65
GHB-$^{13}C_4$	107	**89**	14	14		2.79

Q1: precursor ion. Q3: product ion. DP: declustering potential. CE: collision energy. Rt: retention time. [a]: abundance of the Q3 ions relative to *m/z* 89. [b]: relative abundance of the same GHB-α-ribose Q3 ions: 46/14/4.5/6.0/0.4/100/20/61.

REFERENCES

1. F. P. Busardò, A. W. Jones, Interpreting γ-hydroxybutyrate concentrations for clinical and forensic purposes. *Clin. Toxicol. (Phila.)* **57**, 149–163 (2019).

2. L. Gautam, M. D. Cole, A. Grela, A natural neurotransmitter with negative consequences. *Nat. Chem.* **18**, 606 (2026).

3. K. Skov, S. S. Johansen, K. Linnet, M. K. K. Nielsen, A review on the forensic toxicology of global drug-facilitated sexual assaults. *Eur. Rev. Med. Pharmacol. Sci.* **26**, 183–197 (2022).

4. L. Sardinha, M. Maheu-Giroux, H. Stöckl, S. R. Meyer, C. García-Moreno, Global, regional, and national prevalence estimates of physical or sexual, or both, intimate partner violence against women in 2018. *Lancet* **399**, 803–813 (2022).

5. L. Sardinha, H. Stöckl, M. Maheu-Giroux, S. R. Meyer, C. García-Moreno, Global prevalence of non-partner sexual violence against women. *Bull. World Health Organ.* **102**, 582–587 (2024).

6. L. K. Sørensen, K. B. Faldborg, C. U. Andersen, J. B. Hasselstrøm, Determination of endogenous GHB in ante-mortem whole blood, urine, and oral fluid by LC–MS/MS: The effect of different additives and storage conditions on the stability of GHB in blood. *Forensic Sci. Int.* **365**, 112286 (2024).

7. K. Skov, S. S. Johansen, K. Linnet, M. K. Klose Nielsen, Uncovering forensic evidence of drug-facilitated sexual assault: Toxicological findings from Eastern Denmark from 2015-2022. *Leg. Med. (Tokyo)* **65**, 102325 (2023).

8. M. Palomino-Schätzlein, Y. Wang, A. D. Brailsford, T. Parella, D. A. Cowan, C. Legido-Quigley, M. Pérez-Trujillo, Direct monitoring of exogenous γ-hydroxybutyric acid in body fluids by NMR spectroscopy. *Anal. Chem.* **89**, 8343–8350 (2017).

9. T. Küting, B. Schneider, A. Heidbreder, M. Kramer, P. Jarsiah, B. Madea, C. Hess, Detection of γ-hydroxybutyric acid-related acids in blood plasma and urine: Extending the detection window of an exogenous gamma-hydroxybutyric acid intake? *Drug Test. Anal.* **13**, 1635–1649 (2021).

10. P. Jarsiah, J. Roehrich, T. Kueting, W. Martz, C. Hess, GHB related acids are useful in routine casework of suspected GHB intoxication cases. *Forensic Sci. Int.* **324**, 110833 (2021).

11. P. Jarsiah, J. Roehrich, M. Wyczynski, C. Hess, Phase I metabolites (organic acids) of γ-hydroxybutyric acid-validated quantification using GC-MS and description of endogenous concentration ranges. *Drug Test. Anal.* **12**, 1135–1143 (2020).

12. I. N. Petersen, C. Tortzen, J. L. Kristensen, D. S. Pedersen, T. Breindahl, Identification of a new metabolite of GHB: γ-Hydroxybutyric acid glucuronide. *J. Anal. Toxicol.* **37**, 291–297 (2013).

13. S. Hanisch, N. Stachel, G. Skopp, A potential new metabolite of γ-hydroxybutyrate: Sulfonated γ-hydroxybutyric acid. *Int. J. Leg. Med.* **130**, 411–414 (2016).

14. L.-M. Mehling, T. Piper, A. Spottke, A. Heidbreder, P. Young, B. Madea, M. Thevis, C. Hess, GHB-O-β-glucuronide in blood and urine is not a suitable tool for the extension of the detection window after GHB intake. *Forensic Toxicol.* **35**, 263–274 (2017).

15. T. Piper, L. M. Mehling, A. Spottke, A. Heidbreder, P. Young, B. Madea, C. Hess, W. Schänzer, M. Thevis, Potential of GHB phase-II-metabolites to complement current approaches in GHB post administration detection. *Forensic Sci. Int.* **279**, 157–164 (2017).

16. A. E. Steuer, J. Raeber, F. Simbuerger, D. A. Dornbierer, O. G. Bosch, B. B. Quednow, E. Seifritz, T. Kraemer, Towards extending the detection window of gamma-hydroxybutyric acid—An untargeted metabolomics study in serum and urine following controlled administration in healthy men. *Metabolites* **11**, 166 (2021).

17. A. E. Steuer, F. Bavato, L. K. Schnider, D. A. Dornbierer, O. G. Bosch, B. B. Quednow, E. Seifritz, C. Steuer, T. Kraemer, Urinary concentrations of GHB and its novel amino acid and carnitine conjugates following controlled GHB administration to humans. *Sci. Rep.* **13**, 8983 (2023).

18. K. B. Faldborg, L. K. Sørensen, J. B. Hasselstrøm, C. U. Andersen, Endogenous levels, detection time, and symptoms of gamma-hydroxybutyric acid: Results from a placebo-controlled clinical trial. *Basic Clin. Pharmacol. Toxicol.* **137**, e70133 (2025).

19. K. B. Faldborg, J. B. Hasselstrøm, T. Kraemer, C. U. Andersen, A. E. Steuer, Prolonged detection of GHB intake in urine: Are we finally there? *Drug Test Anal.* **17**, 2429–2446 (2025).

20. L. W. Sumner, A. Amberg, D. Barrett, M. H. Beale, R. Beger, C. A. Daykin, T. W. Fan, O. Fiehn, R. Goodacre, J. L. Griffin, T. Hankemeier, N. Hardy, J. Harnly, R. Higashi, J. Kopka, A. N. Lane, J. C. Lindon, P. Marriott, A. W. Nicholls, M. D. Reily, J. J. Thaden, M. R. Viant, Proposed minimum reporting standards for chemical analysis Chemical Analysis Working Group (CAWG) Metabolomics Standards Initiative (MSI). *Metabolomics* **3**, 211–221 (2007).

21. T. Wang, K. L. Nielsen, K. Frisch, J. K. Lassen, C. B. Nielsen, C. U. Andersen, P. Villesen, M. F. Andreasen, J. B. Hasselstrøm, M. Johannsen, A retrospective metabolomics analysis of gamma-hydroxybutyrate in humans: New potential markers and changes in metabolism related to GHB consumption. *Front. Pharmacol.* **13**, 816376 (2022).

22. B. Lüscher, I. Ahel, M. Altmeyer, A. Ashworth, P. Bai, P. Chang, M. Cohen, D. Corda, F. Dantzer, M. D. Daugherty, T. M. Dawson, V. L. Dawson, S. Deindl, A. R. Fehr, K. L. H. Feijs, D. V. Filippov, J. P. Gagné, G. Grimaldi, S. Guettler, N. C. Hoch, M. O. Hottiger, P. Korn, W. L. Kraus, A. Ladurner, L. Lehtiö, A. K. L. Leung, C. J. Lord, A. Mangerich, I. Matic, J. Matthews, G. L. Moldovan, J. Moss, G. Natoli, M. L. Nielsen, M. Niepel, F. Nolte, J. Pascal, B. M. Paschal, K. Pawłowski, G. G. Poirier, S. Smith, G. Timinszky, Z. Q. Wang, J. Yélamos, X. Yu, R. Zaja, M. Ziegler, ADP-ribosyltransferases, an update on function and nomenclature. *FEBS J.* **289**, 7399–7410 (2022).

23. M. J. Suskiewicz, E. Prokhorova, J. G. M. Rack, I. Ahel, ADP-ribosylation from molecular mechanisms to therapeutic implications. *Cell* **186**, 4475–4495 (2023).

24. S. Jung, S. Kim, Y. Seo, S. Lee, Metabolic alterations associated with γ-hydroxybutyric acid and the potential of metabolites as biomarkers of its exposure. *Metabolites* **11**, 101 (2021).

25. A. E. Steuer, L. Sutter, C. Steuer, T. Kraemer, New gamma-hydroxybutyric acid (GHB) biomarkers: Development and validation of a liquid chromatography-tandem mass spectrometry method for the determination of GHB amino acid, carnitine, and fatty acid conjugates in urine. *Drug Test. Anal.* **15**, 426–443 (2022).

26. C. A. Smith, E. J. Want, G. O'Maille, R. Abagyan, G. Siuzdak, XCMS: Processing mass spectrometry data for metabolite profiling using nonlinear peak alignment, matching, and identification. *Anal. Chem.* **78**, 779–787 (2006).

27. R. Tautenhahn, C. Böttcher, S. Neumann, Highly sensitive feature detection for high resolution LC/MS. *BMC Bioinformatics* **9**, 504 (2008).

28. C. Kuhl, R. Tautenhahn, C. Böttcher, T. R. Larson, S. Neumann, CAMERA: An integrated strategy for compound spectra extraction and annotation of liquid chromatography/mass spectrometry data sets. *Anal. Chem.* **84**, 283–289 (2012).

29. N. Hada, Y. Shida, N. Negishi, F. Schweizer, T. Takeda, Syntheses of glycoclusters containing a phosphocholine residue related to a glycosphingolipid from the earthworm *Pheretima hilgendorfi*. *Chem. Pharm. Bull.* **57**, 1081–1088 (2009).

30. N. Hananya, S. K. Daley, J. D. Bagert, T. W. Muir, Synthesis of ADP-ribosylated histones reveals site-specific impacts on chromatin structure and function. *J. Am. Chem. Soc.* **143**, 10847–10852 (2021).

31. T. Suzuki, S. Watanabe, T. Yamada, K. Hiroi, Dehydrative glycosylation of tri-O-benzylated 1-hydroxyribofuranose catalyzed by a copper(II) complex. *Tetrahedron Lett.* **44**, 2561–2563 (2003).

32. A. B. Mayfield, J. B. Metternich, A. H. Trotta, E. N. Jacobsen, Stereospecific furanosylations catalyzed by bis-thiourea hydrogen-bond donors. *J. Am. Chem. Soc.* **142**, 4061–4069 (2020).

33. P. A. J. Gorin, M. Mazurek, Carbon-13 and proton nuclear magnetic resonance studies on methyl aldofuranosides and their O-alkyl derivatives. *Carbohydr. Res.* **48**, 171–186 (1976).
